# Efficient conversion of primary azides to aldehydes catalyzed by active site variants of myoglobin†
†Electronic supplementary information (ESI) available: Experimental details, synthetic procedures, characterization data for reaction products and additional figures. See DOI: 10.1039/c5sc02857d


**DOI:** 10.1039/c5sc02857d

**Published:** 2015-09-28

**Authors:** Simone Giovani, Ritesh Singh, Rudi Fasan

**Affiliations:** a Department of Chemistry , University of Rochester , 120 Trustee Rd , Rochester , New York 14627 , USA . Email: fasan@chem.rochester.edu

## Abstract

The oxidation of primary azides to aldehydes constitutes a convenient but underdeveloped transformation for which no efficient methods are available. Here, we demonstrate that engineered variants of the hemoprotein myoglobin can catalyze this transformation with high efficiency (up to 8500 turnovers) and selectivity across a range of structurally diverse aryl-substituted primary azides. Mutagenesis of the ‘distal’ histidine residue was particularly effective in enhancing the azide oxidation reactivity of myoglobin, enabling these reactions to proceed in good to excellent yields (37–89%) and to be carried out at a synthetically useful scale. Kinetic isotope effect, isotope labeling, and substrate binding experiments support a mechanism involving heme-catalyzed decomposition of the organic azide followed by alpha hydrogen deprotonation to generate an aldimine which, upon hydrolysis, releases the aldehyde product. This work provides the first example of a biocatalytic azide-to-aldehyde conversion and expands the range of non-native chemical transformations accessible through hemoprotein-mediated catalysis.

## Abstract 2

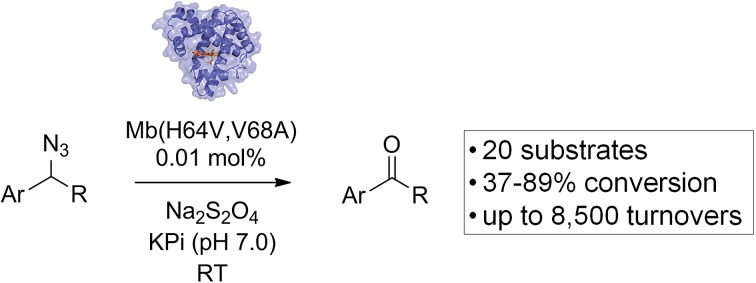
Engineered variants of myoglobin can efficiently catalyze the conversion of primary azides to aldehydes in water and at room temperature

## Introduction

Organic azides constitute one of the most versatile functional groups in organic chemistry, being stable in the presence of moisture and air but unveiling peculiar reactivity upon exposure to transition metals, light or heat.^1^ Important and well established applications of azides in chemical synthesis include their use in Staudinger reductions,^2^ Schmidt reactions,^3^ Curtius rearrangements,^4^ and metal-catalyzed dipolar cycloadditions with alkynes^5^ to give triazoles.^1^ More recently, azides have provided attractive substrates for the synthesis of nitriles^6^ and C–H amination reactions *via* nitrene insertion.^7^ A synthetically valuable but largely underexplored application of primary alkyl azides involves their oxidative transformation to aldehydes. The latter are classically prepared *via* the oxidation of primary alcohols with stoichiometric chromium- and manganese-based oxidizing agents (*e.g.*, pyridinium chlorochromate, MnO_2_) or *via* Swern oxidation, or, alternatively, *via* partial reduction of esters, acid chlorides, or nitriles with metal hydrides. In this context, catalytic strategies to afford aldehydes from azido groups, which can be readily introduced into organic molecules *via* S_N_2 displacement of readily available halides,^8^ would be attractive. In particular, this transformation can provide a convenient, alternative route to the installation of aldehydes from a non-oxygenated precursory functional group. Unfortunately, the very few methods currently available to promote this transformation^9^ require high catalyst loadings (*e.g.*, 10 mol% MoO_2_(S_2_CNEt_2_)_2_)9a,9b and harsh reaction conditions (reflux in toluene/water mixture),9*a* or they lack of selectivity for formation of the aldehyde product.6*c*

Our group has recently reported the ability of heme-dependent metalloproteins such as cytochrome P450s and myoglobin to activate azide substrates such as sulfonyl azides and carbonazidates in the context of intramolecular C(sp^3^)–H amination reactions.7h,7i,^10^ Myoglobin and engineered variants thereof have also proven useful toward promoting carbene transfer reactions starting from diazo-containing substrates.^11^ The reactivity of myoglobin on these classes of substrates prompted us to explore its ability to promote the oxidation of primary azides to the corresponding aldehydes. Here, we report that engineered variants of sperm whale myoglobin can catalyze this transformation with high efficiency and selectivity and across a broad range of substrates, thus providing a first example of a mild, biocatalytic route to the conversion of primary azides to aldehydes.

## Results and discussion

Our studies began with testing the ability of wild-type sperm whale myoglobin to promote the conversion of benzyl azide **1** to benzaldehyde **2a** under anaerobic conditions and in the presence of sodium dithionite (Na_2_S_2_O_4_) as a reductant (Table 1). Gratifyingly, we observed formation of the desired product **2a** albeit only in moderate yield (18%, Entry 3, Table 1). The reaction also produced small amounts of benzylamine **2b** (3%) and *N*-benzyl-benzylimine (**2c**, <2%). **2c** likely arises from condensation of the benzaldehyde product with benzylamine, whereas a possible route for formation of the latter is discussed further below.

**Table 1:** Catalytic activity of wild-type myoglobin (Mb) and its variants in the oxidation of benzyl azide to benzaldehyde^*a*^

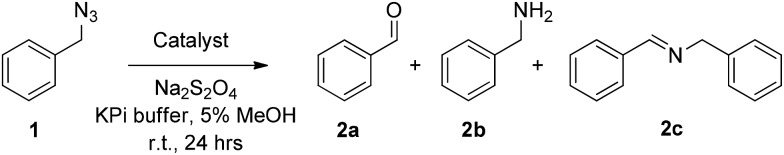					
Entry	Catalyst	[Catalyst]/mM	pH	Conv.^*b*^	TON^*c*^
1	Hemin	0.02	8.0	19%	95
2	Hemin + imidazole (1 mM)	0.02	8.0	22%	110
3	WT Mb	0.001	8.0	18%	1650
4	Mb(L29A)	0.001	8.0	19%	1630
5	Mb(F43W)	0.001	8.0	23%	2190
6	Mb(F43V)	0.001	8.0	32%	2980
7	Mb(H64V)	0.001	8.0	41%	3500
8	Mb(V68A)	0.001	8.0	15%	1420
9	Mb(V68F)	0.001	8.0	6%	580
10	Mb(L29A,H64V)	0.001	8.0	36%	3110
11	Mb(F43V,V68A)	0.001	8.0	42%	3380
13	Mb(H64V,V68A)	0.001	8.0	49%	3740
14	Mb(H64V,V68A)	0.001	7.0	77%	6340

^*a*^Reactions (400 μL) were conducted under anaerobic conditions with 10 mM BnN_3_ and 10 mM Na_2_S_2_O_4_ for 24 hours at room temperature.

^*b*^Product yield based on conversion of initial **1a** to **2a** as determined by gas chromatography.

^*c*^= nmol aldehyde/nmol catalyst. Errors in reported values are within ± 10%.

At a catalyst loading of 0.01 mol%, wild-type myoglobin was found to support about 1650 catalytic turnovers (TON) for the conversion of **1** to **2a**. Under identical conditions but at higher catalyst loading (0.2 mol%), free hemin gave only 95 TON (Entry 1, Table 1). Given the presence of a histidine-coordinated heme in Mb^12^ and the impact of axial ligands in affecting the reactivity of metalloporphyrin systems,^13^ the same experiment was repeated in the presence of imidazole (Entry 2, Table 1). The low catalytic activity observed also in this case further highlighted the critical role of the protein scaffold in endowing the hemoprotein with high azide oxidation reactivity. Additional control experiments showed a nearly complete loss of this activity in the presence of air, or in the presence of carbon monoxide (which forms a stable complex with ferrous Mb), or in the absence of the reductant (Na_2_S_2_O_4_), indicating the direct involvement of Mb heme center, in its ferrous state, in the catalytic transformation.

**Fig. 1:**
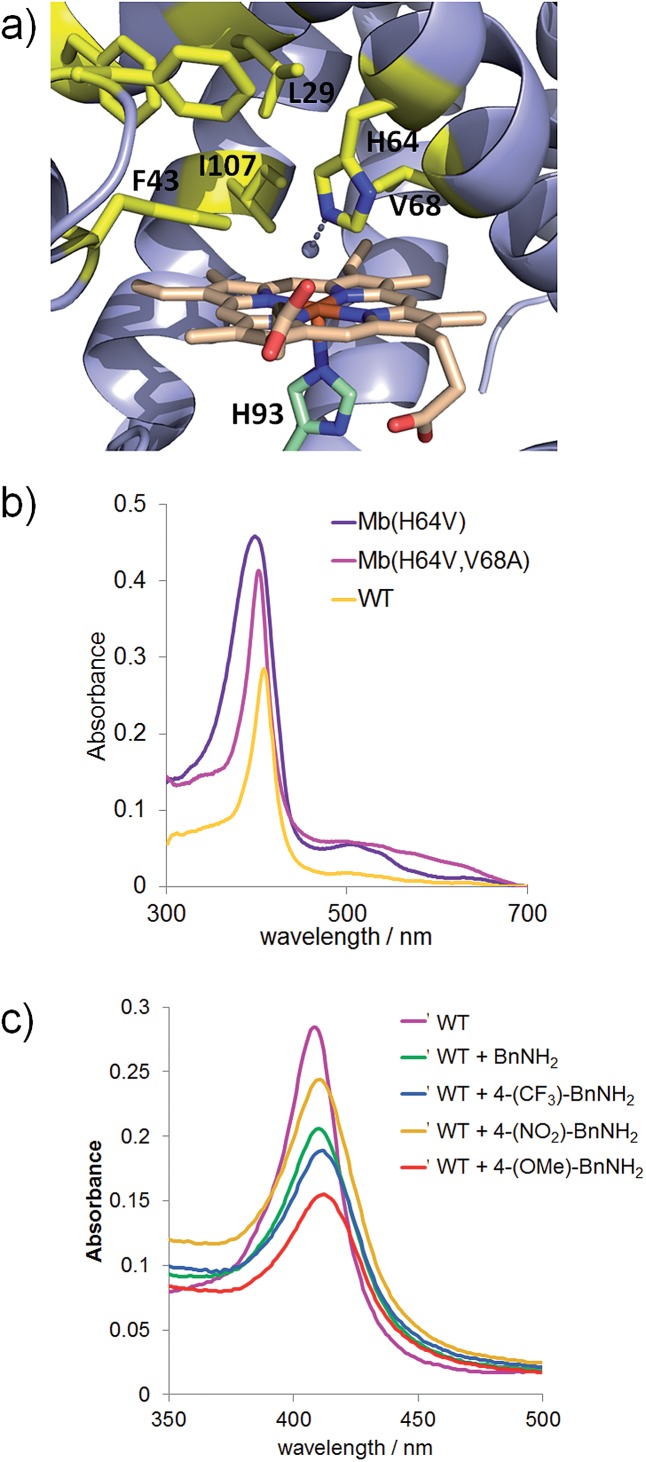
(a) Distal pocket of sperm whale myoglobin (pdb 1A6K 12). Active site residues (yellow), heme (wheat), ‘proximal His’ (green), and heme-bound water molecule (grey sphere) are highlighted. (b) Electronic absorption spectra for wild-type Mb and Mb variants Mb(H64V) and Mb(H64V,V68A) in phosphate buffer at pH 7.0. The Soret bands have maxima at 408, 398, and 403 nm, respectively. (c) Ligand-induced decrease of Soret band intensity upon incubation of Mb in phosphate buffer at pH 7 with 20 mM benzyl amine (BnNH_2_) and *para*-substituted BnNH_2_ derivatives (*λ*_max_ = 408 nm (WT); 409 nm (WT + BnNH_2_); 410 nm (WT + 4-(CF_3_)–BnNH_2_); 409 nm (WT + 4-(NO_2_)–BnNH_2_); 412 nm (WT + 4-(OMe)–BnNH_2_).

In our previous studies on myoglobin-catalyzed carbene transfer reactions,^11^ we observed a distinctive effect of active site mutations on the efficiency and selectivity of these transformations. As illustrated in Fig. 1a, five amino acid residues (L29, F43, H64, V68, I107) line up the distal pocket of myoglobin. To identify improved Mb-based catalysts for the reaction investigated here, a panel of Mb variants carrying either a single or a double amino acid substitution at the level of these residues were tested in the reaction with benzyl azide (Table 1). These mutations were designed to vary the geometry of the active site through decreasing (*e.g.* L29A) or increasing (*e.g.* F43W) the size of the side-chain group. Among the single-site Mb variants, both Mb(F43W) and Mb(H64V) display higher catalytic activity than the wild type protein (Entries 5 and 7, Table 1). Notably, Mb(H64V) was found to support about 3500 TON for the conversion of **1** to **2a**. A further improvement in activity (∼20%) was then achieved by combining the H64V and V68A mutations, thus defining Mb(H64V,V68A) variant as the most promising biocatalyst for further investigation (3740 TON, Table 1). Using this variant, about half of the azide substrate (49%) could be converted to benzaldehyde, this corresponding to a three-fold higher conversion than that obtained with the native hemoprotein. Interestingly, further optimization experiments showed a marked pH dependence of the relative amount and distribution of the products **2a**, **2b** and **2c**, with the plot of TON for benzaldehyde (**2a**) *versus* pH yielding a bell-shaped curve with a maximal value around neutral conditions (ESI Fig. S1†). At pH 7, the Mb(H64V,V68A)-catalyzed oxidation of benzyl azide to benzaldehyde proceeds with an improved GC yield of 77% and correspondingly higher turnovers (6340 TON, Table 1), while the amount of the byproduct **2b** remains as low as or lower than that formed at higher pH (4% *vs.* 4–11%). Time-dependent studies showed that the benzyl azide substrate is oxidized at a rate of 250 turnovers per min over the first 10 minutes and completely consumed within 1.5 hours (ESI Fig. S2†). Notably, the product conversion with Mb(H64V,V68A) under these optimized conditions matches those reported for the same reaction in the presence of the Mo-based complex9*a* at high temperature (100 °C) and employing 1000-fold higher catalyst loadings, thus highlighting the efficiency and mildness of the biocatalytic process in the context of this transformation.

**Scheme 1:**
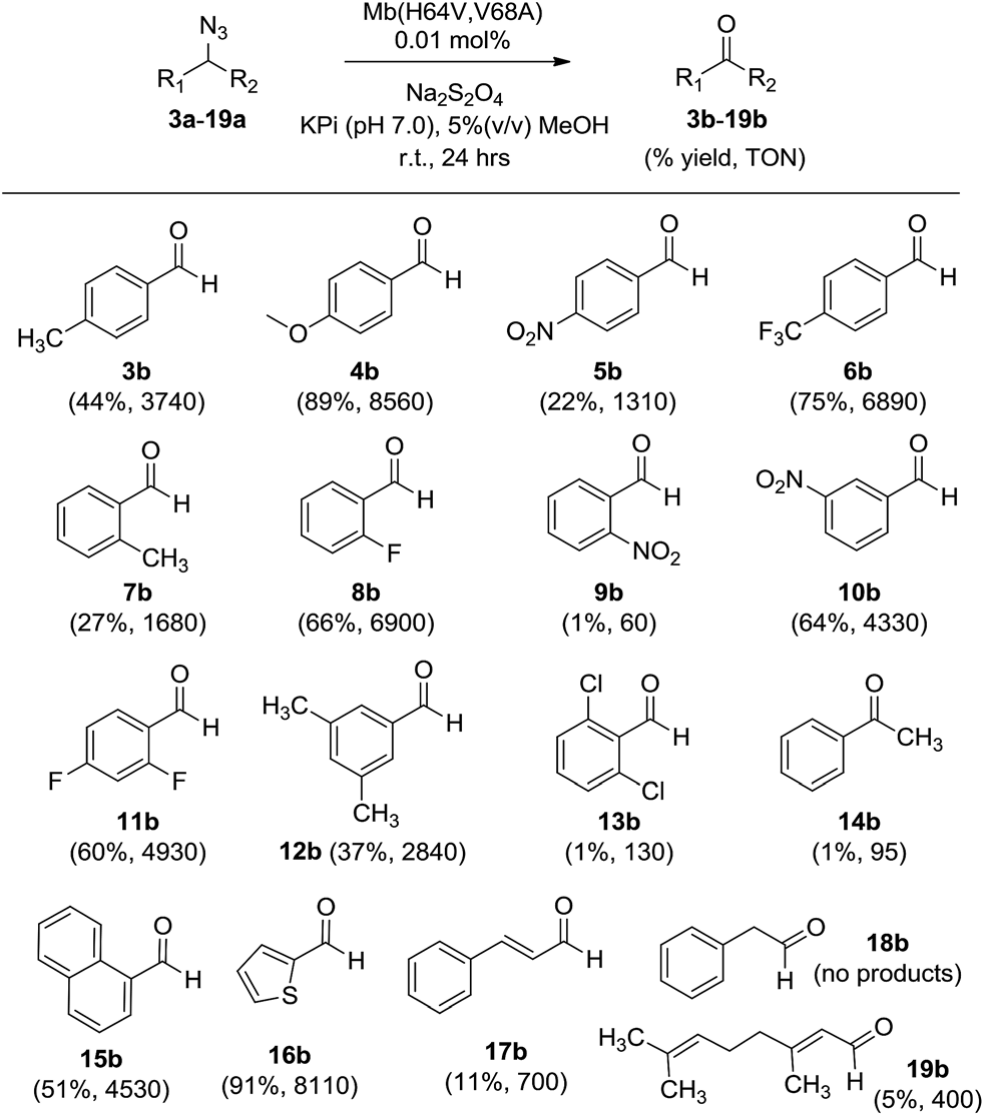
Substrate scope for Mb(H64V,V68A)-catalyzed azide oxidation. Reaction conditions: 10 mM azide, 10 mM Na_2_S_2_O_4_, 1 μM Mb(H64V,V68A).

To examine the scope of the Mb-mediated transformation in more detail, various aryl substituted azidomethyl substrates (**3a–19a**) were prepared and subjected to Mb(H64V,V68A)-catalyzed oxidation. As summarized in Scheme 1, the majority of these substrates could be converted to the corresponding aldehyde in good to excellent yields (44–89%), with the Mb catalyst supporting from 3740 (**3b**) to over 8500 turnovers (**4b**). These experiments also revealed a significant impact of both electronic and steric factors on the efficiency of the reaction. The synthesis of **3b**, **4b**, **6b**, and **10b** in 44–89% yields indicated that substitutions at the *para* and *meta* positions of the phenyl ring, including double *meta* substitutions (**12b**), are generally well tolerated by the Mb catalyst. Significantly less so are *ortho* substitutions, these resulting in generally lower conversions as illustrated by the results with **7b** (27%) and in particular with **9b** and **13b**, for which nearly no product formation was observed. Notable exceptions to this trend are substrates containing *ortho* modifications with small fluorine substituents (**8a**, **11a**) or fused ring, such as 1-(azidomethyl)naphthalene **15a**, all of which could be efficiently oxidized (51–66%) to the corresponding aldehydes (**8b**, **11b**, and **15b**, respectively).

In addition to steric factors, the influence of electronic effects also became apparent from these studies. Interestingly, high conversions and catalytic turnovers (4440–8560) were observed in the presence of benzyl azide substrates with electron-rich aromatic rings by virtue of electron donating substituents (**4b**), heteroatoms (**16b**), or fused rings (**15b***vs.***7b**), whereas reduced TTN as compared to benzyl azide (**1**) were observed in the presence of substrates with electron poor aromatic rings (**5b**). Notably deviating from this trend are the fluorinated substrates, 4-trifluoromethyl- (**6a**), 2-fluoro- (**8a**), and 2,4-difluoro-benzyl azide (**11a**), which in spite of the electron withdrawing effect of the fluorine substituents are effectively oxidized by the Mb catalyst (4930–6890 TON, Scheme 1).

Next, we tested substrates such as cinnamyl azide (**17a**), (2-azidoethyl)benzene (**18a**), and geranyl azide (**19a**) to investigate the scope of the Mb-catalyzed reaction across compounds lacking an aryl substituent in alpha to the azido group. Although in moderate yields (∼10%), formation of cinnamaldehyde (**17b**) and citral (**19b**) was observed, whereas no reaction occurred in the presence of **18a**. Of note, the Mb-mediated oxidation of cinnamyl azide (**17a**) gave only *E*-citral, while a 2 : 1 mixture of *E*- and *Z*-citral was obtained by PCC oxidation of geraniol as reported previously.^14^ As indicated by the catalytic turnovers for the corresponding aldehyde products (700 (**17b**) > 400 (**19b**) > 0 TON (**18b**)), the order of reactivity of the substrates above is consistent with the higher reactivity of electron rich azides observed across the substituted benzyl azide derivatives. Altogether, these results suggest a clear requirement for the (hetero)aromatic ring linked to the azidomethyl group for efficient transformation by the Mb catalyst. Finally, the poor conversion of (1-azidoethyl)benzene (**14a**) to acetophenone (**14b**) showed that oxidation of secondary azides by Mb(H64V,V68A) is considerably less efficient than that of primary azides (6340 *vs.* 95 TON for **2b***vs.***14b**).

To assess the scalability of these transformations, a large scale reaction with benzyl azide **1** (30 mg) was carried out in the presence of 0.05 mol% Mb(H64V,V68A) in phosphate buffer at room temperature. After a simple extraction and purification step, the desired benzaldehyde product **2a** could be obtained in 80% isolated yield (19 mg), thus providing a proof-of-principle demonstration of the synthetic utility of this biocatalytic transformation.

**Scheme 2:**
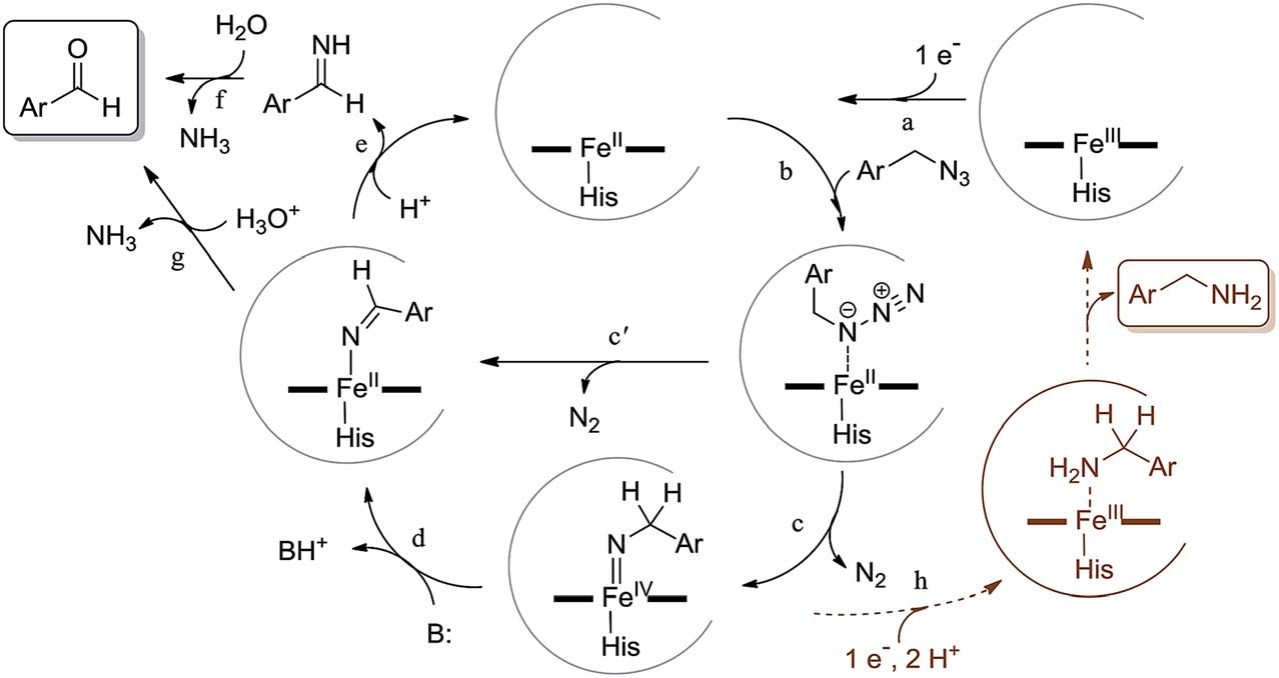
Proposed mechanism and catalytic steps for the myoglobin-catalyzed oxidation of alkyl azides to aldehydes (black). The pathway leading to the minor alkyl amine byproduct is also shown (brown).

Scheme 2 outlines our proposed mechanism for the Mb-catalyzed azide-to-aldehyde oxidation reaction reported here. After reduction of ferric Mb to the catalytically active ferrous form (step ‘a’), we envision formation of an initial alkyl azide–heme complex following binding of the substrate to the distal pocket of the protein (step ‘b’). This species could then undergo loss of N_2_ to yield a nitrene-heme species, possibly in the form of an imido-iron(iv) intermediate (step ‘c’). These steps are analogous to those envisioned to precede the nitrene C–H insertion step in the context of our previously investigated Mb- and P450-catalyzed intramolecular C–H amination reactions with arylsulfonyl azides and carbonazidates.7h,7i,^10^ Unlike the latter, however, the presence of an α-hydrogen in the azide substrates employed here could allow for the tautomerization of the imido-iron(iv) species to an imine-iron(ii) complex (step ‘d’), whose dissociation releases an aldimine and regenerates ferrous Mb (step ‘e’). Hydrolysis of the aldimine either in free form (step ‘f’), or while still bound to the heme (step ‘g’), then yields the aldehyde product. An alternative path for the formation of the imine directly from the alkyl azide–heme complex (step ‘c′’) is through a 1,2 hydrogen shift analogous to that observed for free alkyl nitrenes obtained by azide photolysis.^15^ This type of rearrangement has been predicted to be energetically barrierless for singlet nitrene species, but it is associated with an energy barrier for triplet nitrenes.^16^ Since the putative imido-iron(iv) intermediate in Scheme 2 is likely to have a non-singlet electronic state (*i.e.*, *S* = 2) according to Density Functional Theory (DFT) calculations^17^ on related species,^18^ a path proceeding through this intermediate appears to be more plausible. In addition, a barrierless nitrene-to-imine conversion would not be consistent with the kinetic isotope effect (KIE) observed upon deuteration of the α-position (*vide infra*).

Several experimental data were found to be supportive of the mechanism presented in Scheme 2. Reasonably, formation of the initial azide–heme complex (step ‘b’) is favoured for electron-rich alkyl azides, which is consistent with the generally higher TON supported by Mb(H64V,V68A) for the oxidation of azide substrates containing electron-rich aryl substituents (*e.g.*, **4a**, **15a**, **16a**). To further examine this aspect, binding studies were carried out with Mb and different *para*-substituted benzyl amines as unreactive mimics of the corresponding azides (Fig. 1c). Binding of the amine ligands to the heme cofactor in Mb resulted in a red shift (Δ*λ*_max_ = 2–3 nm) and decrease in intensity of the Soret band (410 nm) in the electronic absorption spectrum of the hemoprotein, in line with previous observations.^19^ Furthermore, incubation of the amine-treated protein with dithionite and CO yielded a Mb–CO complex spectroscopically superimposable with that of untreated Mb (ESI Fig. S3†), confirming that the amine complex is reversible and that no protein denaturation had occurred. Importantly, the amine-induced effect was found to increase in the order *p*-nitro-benzyl amine > benzyl amine > *p*-methoxy-benzyl amine (Fig. 1c), which is in excellent agreement with the activity trend observed for the oxidation of the corresponding azides (**5a** < **1** < **4a**). In this regard, the large activity enhancement associated with the H64V mutation is also interesting (Table 1). In deoxy Mb, the ‘distal’ His64 residue contributes to stabilize a water molecule ligand occupying the sixth coordination site of the heme group *via* hydrogen bonding (Fig. 1a).^20^ Substitution of His64 with valine removes this interaction, leading to a five coordinate heme center as determined by X-ray crystallography.^21^ Spectroscopically, this feature is evidenced by the blue shift of the Soret band from 408 nm for ferric wild-type Mb to 398–402 nm for Mb(H64V) and Mb(H64V,V68A), as shown in Fig. 1b. Thus, the H64V mutation could promote catalysis by favouring binding of the alkyl azide substrate to the axial coordination site of the heme group. Furthermore, removal of the heme-bound water molecule as a result of the H64V mutation makes the distal pocket of the hemoprotein largely hydrophobic.^21^ This phenomenon can account for the efficient oxidation of the fluorine-containing azide substrates (**6a**, **8a**, **11a**), whose increased lipophilicity (compared to benzyl azide) is thus expected to favour binding to the hydrophobic active site of the H64V-containing Mb variants. This conclusion is consistent with the larger heme spin shift for binding of 4-(trifluoromethyl)benzyl amine to Mb as compared to the benzyl amine (Fig. 1c).

**Fig. 2:**
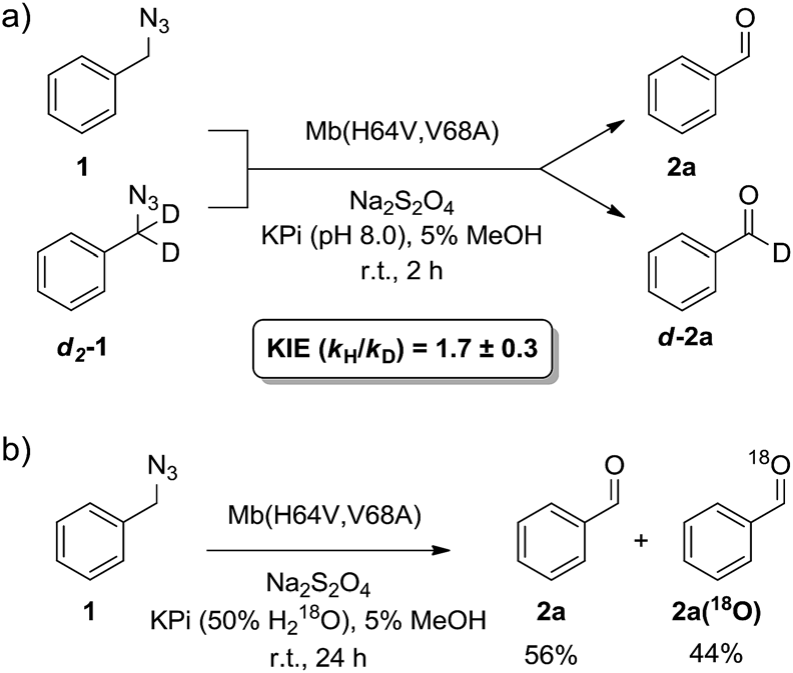
(a) Measurement of kinetic isotope effect (KIE) for H/D substitution of αC–H bond on Mb(H64V,V68A)-catalyzed oxidation of benzyl azide. See also ESI Fig. S4.† (b) Isotope labelling experiment demonstrating ^18^O incorporation in the aldehyde product in the presence of H_2_^18^O-containing buffer. See also ESI Fig. S5.†

**Scheme 3:**
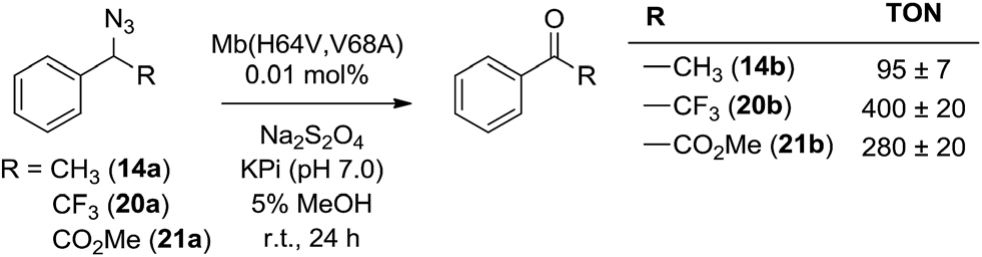
Mb(H64V,V68A)-catalyzed oxidation of secondary alkyl azides.

α-Hydrogen deprotonation constitutes a rate-limiting step in the enolization of ketones and aldehydes, as demonstrated by kinetic isotopic effect (KIE) experiments (*k*_H_/*k*_D_ = 4–6).^22^ By analogy, the mechanism outlined in Scheme 2 would predict a decrease in rate upon H → D substitution of the α-hydrogen in the azide substrate. To examine this aspect, a competition experiment was performed using a mixture of benzyl azide **1** and its deuterated analog **d_2_-1** in the presence of Mb(H64V,V68A) as the catalyst (Fig. 2a). LC-MS analysis of this reaction yielded a KIE value (*k*_H_/*k*_D_) of 1.7 ± 0.3 at 22 °C (ESI Fig. S4†), thus demonstrating that deprotonation of the αC–H bond is part of the rate-determining step of the reaction. Incidentally, this KIE value is comparable to that measured for the imine-to-aldimine tautomerization step implicated in the l-Ala ↔ d-Ala isomerization reaction catalyzed by the PLP-dependent alanine racemase (*k*_H_/*k*_D_ = 1.3–1.9).^23^ The mechanistic insights gathered from the KIE studies suggested that increasing the acidity of the α-hydrogen would favour Mb-catalyzed azide oxidation. To test this hypothesis, the oxidation activity of Mb(H64V,V68A) on (1-azidoethyl)benzene (**14a**) was compared with that observed in the presence of two related substrates, **20a** and **21a** (Scheme 3), in which the benzylic C–H site is rendered more acidic because of the adjacent trifluoromethyl and ester group, respectively. In line with our expectations, three to four-fold higher TON were measured for the formation of **20b** and **21b** as compared to **14b** (Scheme 3), thus providing further support to the importance of the α-H deprotonation step in the Mb-catalyzed alkyl azide oxidation reaction.

The step involving the release of the aldehyde product from the aldimine intermediate (step ‘f’ or ‘g’ in Scheme 2) was then probed by repeating the reaction with Mb(H64V,V68A) and benzyl azide in the presence of ^18^O-labeled water (Fig. 2b). GC-MS analysis showed a degree of ^18^O incorporation into the benzaldehyde product (44%) that closely matched the ratio of H_2_^18^O in the reaction mixture (50%) (ESI Fig. S5†), thus providing direct evidence for the occurrence of the hydrolysis step as part of the mechanism of the reaction. Combined together, the KIE, isotopic labelling, and Mb binding experiments support the reaction pathway described in Scheme 2. Interestingly, this reaction sequence is fundamentally distinct from that proposed for azide oxidation mediated by a molybdenum-based catalyst, which requires oxygen and purportedly involves an oxoaziridine and oxime intermediates.9*a*

As noted earlier, the reaction with benzyl azide also results in the formation of **2b** and **2c** (Table 1) as minor byproducts (<5%). Small amounts (0.1–10%) of related amine and imine species were observed also in the presence of the most reactive substrates described in Scheme 1. Whereas **2c** can simply arise from the condensation of **2a** with **2b**, the benzylamine byproduct is akin to the sulfonamide and carbamate products observed during P450-catalyzed C–H amination with sulfonyl azides and carbonazidates.7h,7i,^10^ In analogy to those reactions, we envision this species originates from overreduction and protonation of the putative imido-iron intermediate (step ‘h’, Scheme 2). It is interesting to note, however, how this side reaction is largely disfavored in the context of azide oxidation as compared to these C–H amination processes. This difference can be attributed at least in part to the ease by which the alkyl azide can undergo α-proton elimination as compared to nitrene C–H insertion, as suggested by our KIE experiments (*i.e. k*_H_/*k*_D_ of 1.7 for azide oxidation as compared to 2.5–5 for C–H amination7i,^10^).

## Conclusions

In conclusion, we report the first example of a mild, biocatalytic method for the oxidative conversion of primary azides to aldehydes. Using an engineered active site variant of myoglobin as the catalyst, this valuable yet underdeveloped transformation could be afforded with high efficiency and selectivity across a broad range of aryl-substituted primary azide substrates. Importantly, the catalytic turnovers supported by the Mb catalyst at room temperature and in buffer exceed by orders of magnitudes those previously obtained with synthetic catalysts in organic solvents and at high temperature. Kinetic isotope effect, isotope labeling, and substrate binding experiments support a mechanism that involves heme-catalyzed decomposition of the alkyl azide followed by α-hydrogen deprotonation to generate an aldimine which, upon hydrolysis, gives the aldehyde product. As suggested by the data presented in Scheme 3, these mechanistic insights can prove valuable toward expanding the scope of these Mb-based catalysts and guiding their further optimization in the context of this and related reactions. Finally, the Mb-dependent azide oxidase activity reported here expands the range of synthetically valuable transformations accessible through engineered and artificial metalloprotein catalysts.7h,7j,^11^,^24^

## Supplementary Material

Supplementary information

## References

[cit1] Brase S., Gil C., Knepper K., Zimmermann V. (2005). Angew. Chem., Int. Ed..

[cit2] Staudinger H., Meyer J. (1919). Helv. Chim. Acta.

[cit3] Schmidt K. F. (1924). Ber. Dtsch. Chem. Ges..

[cit4] Curtius T. (1890). Ber. Dtsch. Chem. Ges..

[cit5] Kolb H. C., Finn M. G., Sharpless K. B. (2001). Angew. Chem., Int. Ed..

[cit6] Lamani M., Prabhu K. R. (2010). Angew. Chem., Int. Ed..

[cit7] Cenini S., Tollari S., Penoni A., Cereda C. (1999). J. Mol. Catal. A: Chem..

[cit8] Scriven E. F. V., Turnbull K. (1988). Chem. Rev..

[cit9] Maddani M., Prabhu K. R. (2008). Tetrahedron Lett..

[cit10] Bordeaux M., Singh R., Fasan R. (2014). Bioorg. Med. Chem..

[cit11] Bordeaux M., Tyagi V., Fasan R. (2015). Angew. Chem., Int. Ed..

[cit12] Vojtechovsky J., Chu K., Berendzen J., Sweet R. M., Schlichting I. (1999). Biophys. J..

[cit13] Higuchi T., Shimada K., Maruyama N., Hirobe M. (1993). J. Am. Chem. Soc..

[cit14] Nonaka T., Kanemoto S., Oshima K., Nozaki H. (1984). Bull. Chem. Soc. Jpn..

[cit15] Barton D. H. R., Morgan L. R. (1962). J. Chem. Soc..

[cit16] Pople J. A., Raghavachari K., Frisch M. J., Binkley J. S., Schleyer P. V. (1983). J. Am. Chem. Soc..

[cit17] Conradie J., Ghosh A. (2010). Inorg. Chem..

[cit18] Mansuy D., Battioni P., Mahy J. P. (1982). J. Am. Chem. Soc..

[cit19] Kato S., Yang H. J., Ueno T., Ozaki S., Phillips Jr G. N., Fukuzumi S., Watanabe Y. (2002). J. Am. Chem. Soc..

[cit20] Yang F., Phillips G. N. (1996). J. Mol. Biol..

[cit21] Quillin M. L., Arduini R. M., Olson J. S., Phillips Jr G. N. (1993). J. Mol. Biol..

[cit22] Emmons W. D., Hawthorne M. F. (1956). J. Am. Chem. Soc..

[cit23] Spies M. A., Toney M. D. (2003). Biochemistry.

[cit24] Wilson M. E., Whitesides G. M. (1978). J. Am. Chem. Soc..

