# Supplementary material for: Efficient conversion of primary azides to aldehydes catalyzed by active site variants of myoglobin
Source: Chem Sci. 2015 Sep 28;7(1):234–9. doi: 10.1039/c5sc02857d (PMC4754990; doi:10.1039/c5sc02857d)
Supplement: Supplementary file 1 [file SC-007-C5SC02857D-s001.pdf]

Electronic Supplementary Material (ESI) for Chemical Science.
This journal is © The Royal Society of Chemistry 2015

Supporting Information for

Efficient conversion of primary azides to aldehydes catalyzed by active site variants of myoglobin

Simone Giovani[#], Ritesh Singh[#], and Rudi Fasan*

Department of Chemistry, University of Rochester, 14627 Rochester, New York, USA

Correspondence should be addressed to R.F. (fasan@chem.rochester.edu)

[#] *These authors contributed equally to this work.*

Table of contents:

Supplementary Figure S1-S7	Pages S2-S13
Experimental Procedures	Pages S14-S16
Synthetic Procedures and Characterization Data	Pages S17-S22
References	Page S23
Spectra	Pages S24-S49

Figure S1. pH dependence of Mb(H64V,V68A)-catalyzed benzyl azide (**1**) oxidation. a) Plot of catalytic turnovers (TON) for formation of benzaldehyde (**2a**) at varying pH values ranging from 6.0 to 8.0. b) Plot describing the relative distribution of benzaldehyde (**2a**), benzylamine (**2b**), and N-benzyl-benzylimine (**2c**) products for reactions carried out at the varying pH. Reaction conditions: 400 µL-scale reaction containing 1 µM Mb(H64V,V68A), 10 mM **1**, 10 mM $Na_2S_2O_4$ in phosphate buffer at the specified pH value, room temperature, 24 hours.

pH	TON	$\%_{CHO}$	$\%_{Imine}$	$\%_{Amine}$
**6.0**	3360	36	0	0
**6.5**	5570	68	1.3	0.5
**7.0**	6340	77	9.2	4.4
**7.5**	5080	66	15	11
**8.0**	3650	52	8.5	3.8

a)

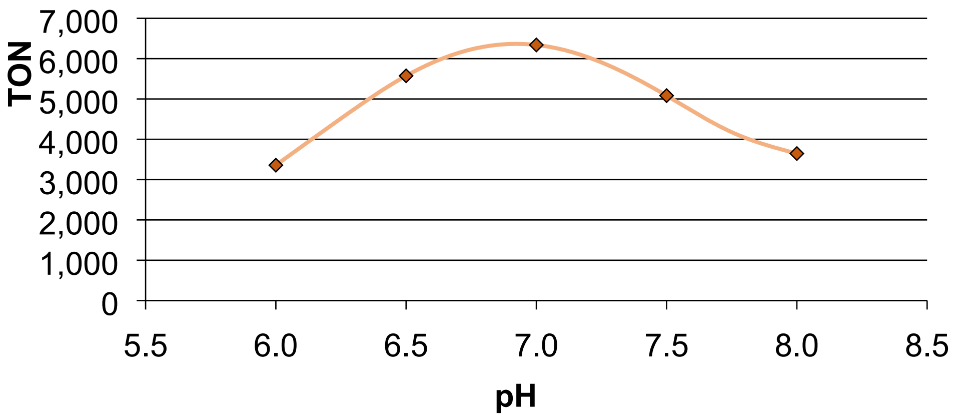

b)

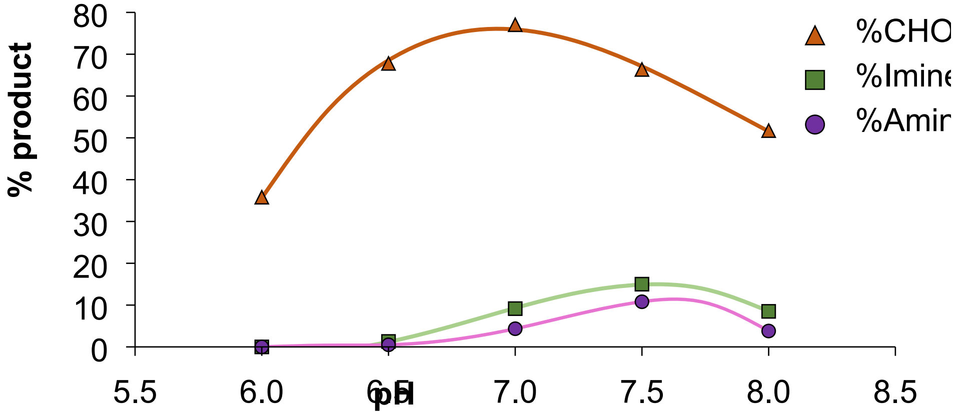

Figure S2. Time-dependent consumption of benzyl azide (**1**) substrate in the Mb(H64V,V68A)-catalyzed reaction as determined by gas chromatography. Reaction conditions: 1 μM Mb(H64V,V68A), 10 mM BnN_3, 10 mM $Na_2S_2O_4$ in KPi buffer (pH = 7.0).

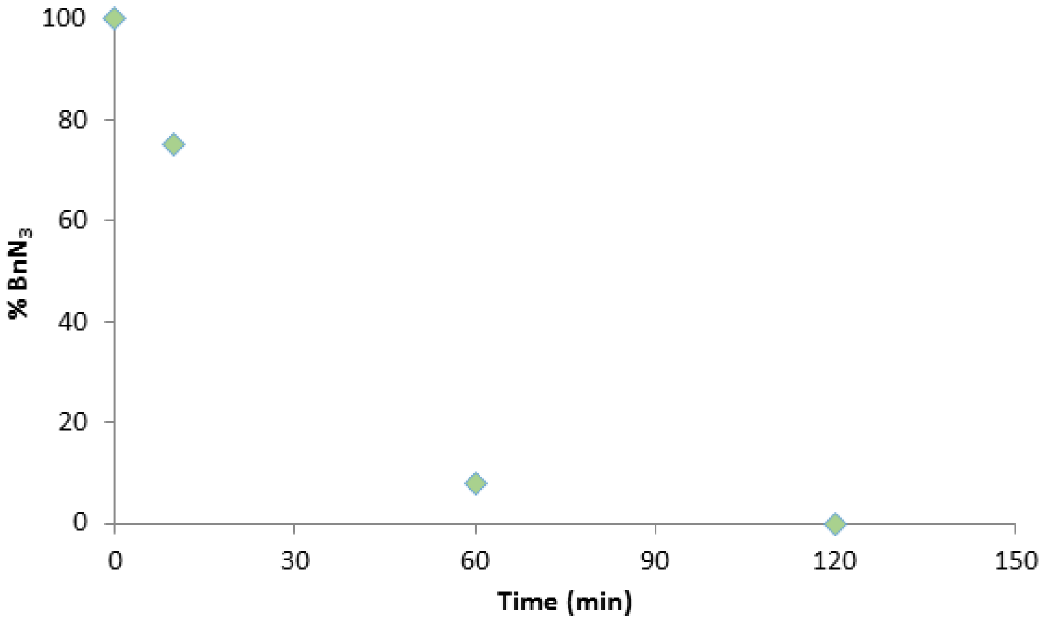

Figure S3. UV-vis spectra corresponding to wild-type myoglobin (WT Mb) before (dark red line) and after (light red line) addition of 20 mM benzylamine ($BnNH_2$) and after reduction and incubation with CO (pink line). The UV-vis spectra of untreated Mb in ferric form (dark green line) and as ferrous CO-complex (light green line) are included for comparison.

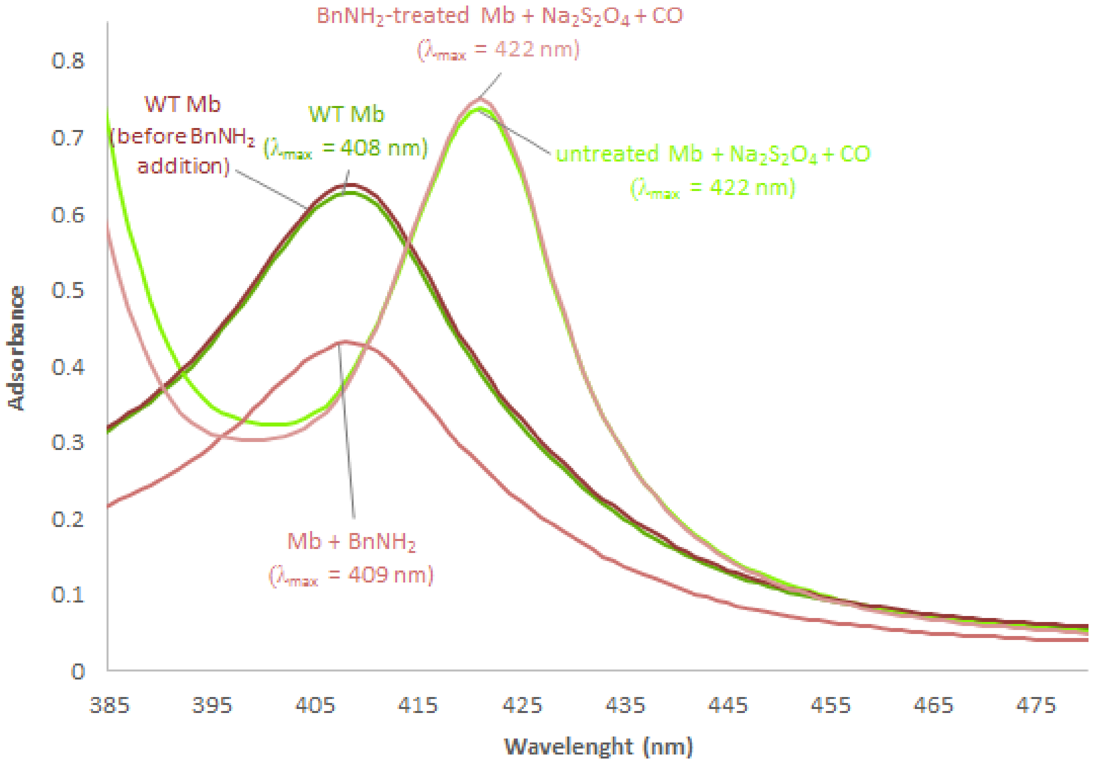

Figure S4. H/D competition experiments. A) GC-MS spectrum of the mixture of benzyl azide (**1**; *m/z* 133.2) and deuterated benzyl azide (***d₂*-1**; *m/z* 135.2) prior to the reaction with Mb(H64V,V68A) (**1** : ***d₂*-1** ratio of 1 : 0.8). B) GC-MS spectrum of the benzaldehyde (**2a**) and deuterated benzaldehyde (***d*-2a**) products after the reaction with Mb(H64V,V68A) and the **1** / ***d₂*-1** mixture. The KIE value were obtained from integration of the MS signals corresponding to the molecular ions of **2a** (*m/z* 106.1) and ***d*-2a** (*m/z* 107.1). Reaction conditions: 400 μL-scale reaction containing 5 μM Mb(H64V,V68A), 10 mM **1** + ***d₂*-1**, 10 mM $Na_2S_2O_4$ in phosphate buffer (pH 8.0) at room temperature for 2 hours.

A)

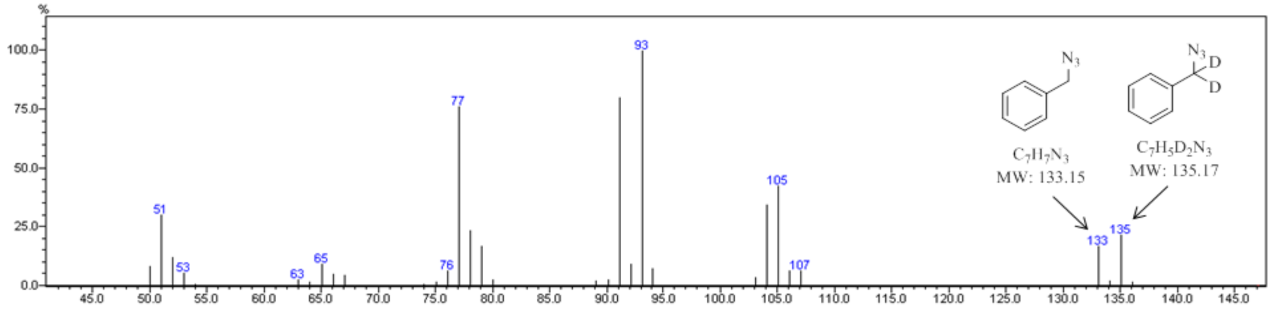

B)

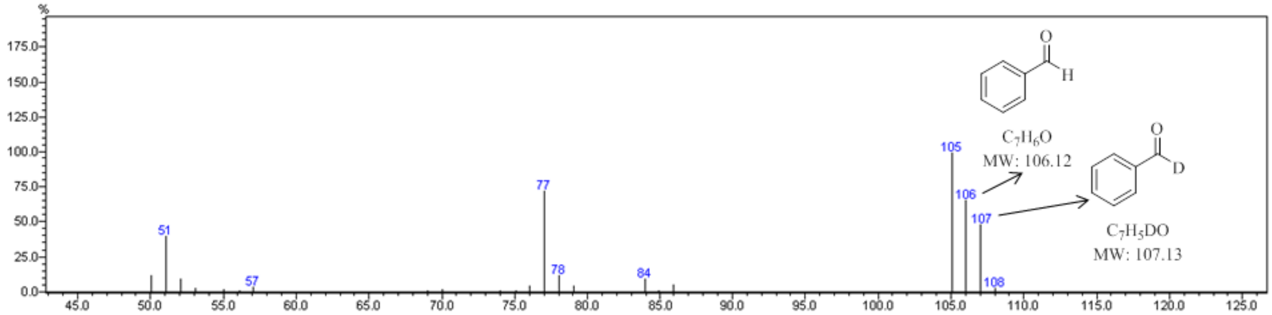

Figure S5. ^{18}O labeling experiments. GC-MS spectrum of benzaldehyde (**2a**) and ^{18}O-containing benzaldehyde- (**2a(^{18}O)**) formed upon the reaction of Mb(H64V,V68A) with benzyl azide (**1**) in the presence of 50% $H_2{}^{18}O$ under standard reaction conditions (1 μM protein, 10 mM benzyl azide, 10 mM sodium dithionite in KPi (50% $H_2{}^{18}O$) at pH 7.0 (not adjusted), room temperature, 24 hours). The isotopic distribution (56 % ^{16}O, 44% ^{18}O) was calculated from integration of the MS signals corresponding to the molecular ions of **2a** (*m/z* 106.1) and **2a(^{18}O)** (*m/z* 108.1).

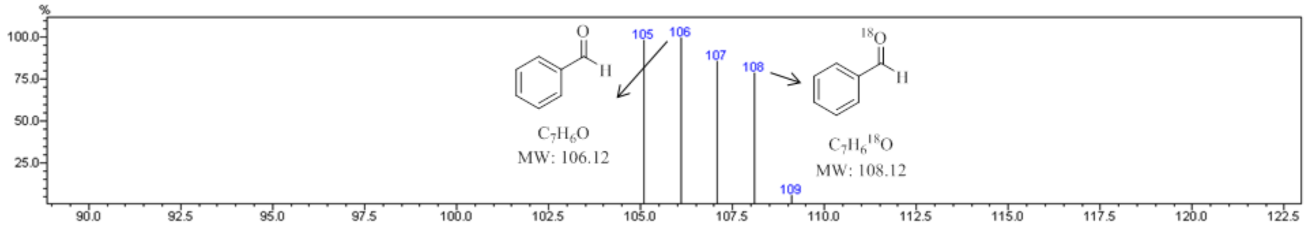

Figure S6. Calibration curve for the aldehyde products. All the calibration curves were realized adding a variable concentration of the corresponding aldehyde in methanol (8, 4, 2, 1 and 0.5 mM, respectively) to 400 μL of a Kpi solution at pH 7. The mixture was added with the internal standard (benzodioxole, 20 μL from a 5 mM stock solution), extracted with dichloromethane (400 μL), and analyzed by GC.

a) Calibration curve for Benzyl azide

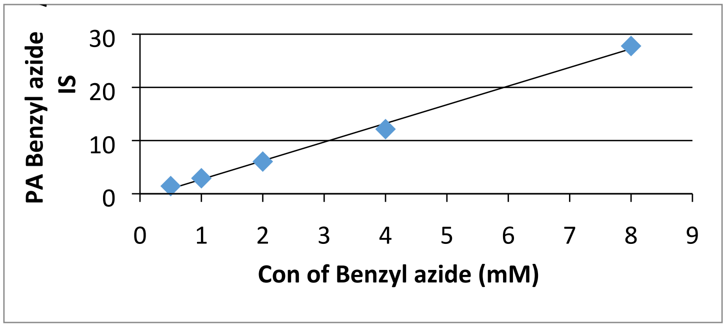

b) Calibration curve for Benzaldehyde

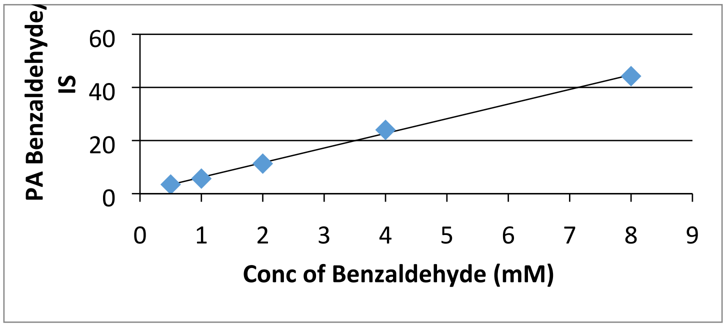

c) Calibration curve for Benzylamine

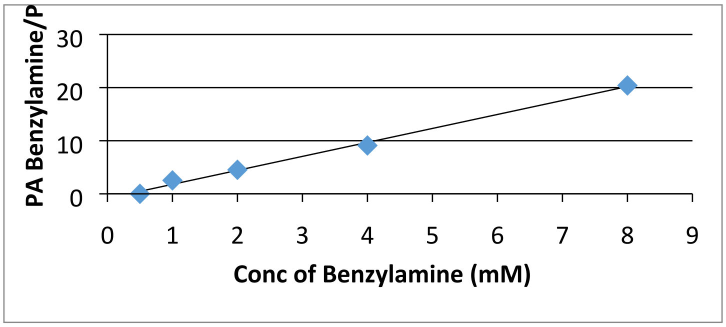

d) Calibration curve for *N*-Benzyl-1-benzylimine

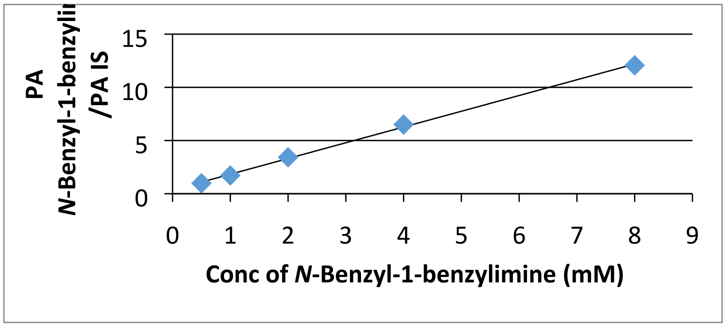

e) Calibration curve for 4-Tolualdehyde

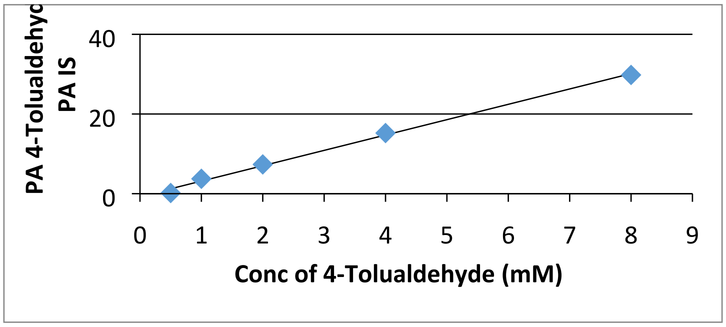

f) Calibration curve for Anisaldehyde

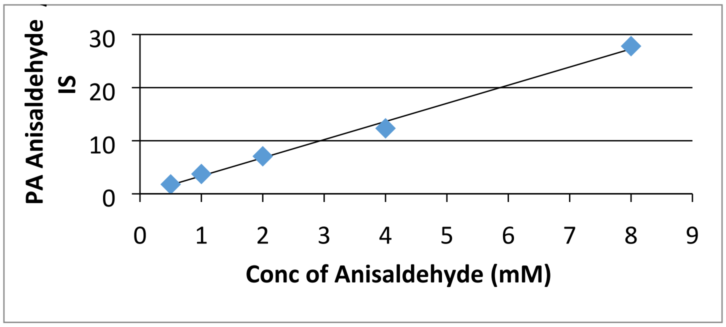

g) Calibration curve for 4-Nitrobenzaldehyde

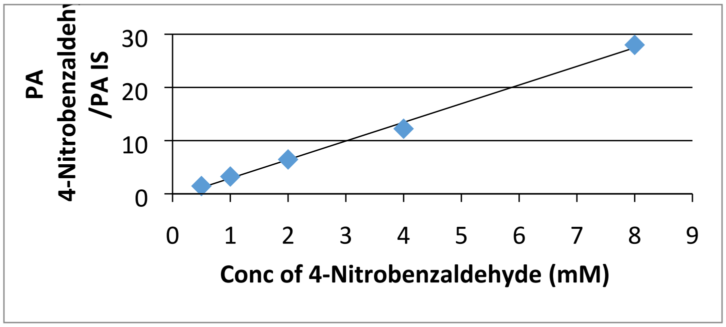

h) Calibration curve for 4-(Trifluoromethyl)benzaldehyde

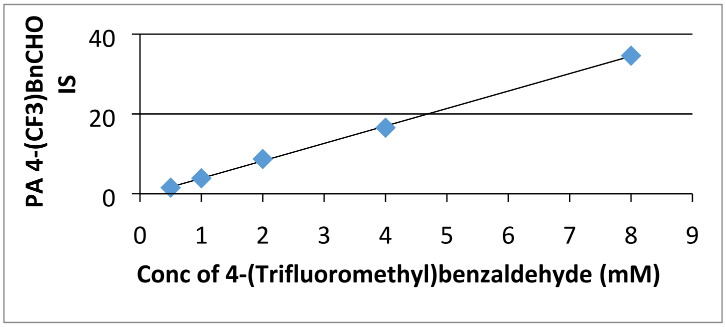

i) Calibration curve for 2-Tolualdehyde

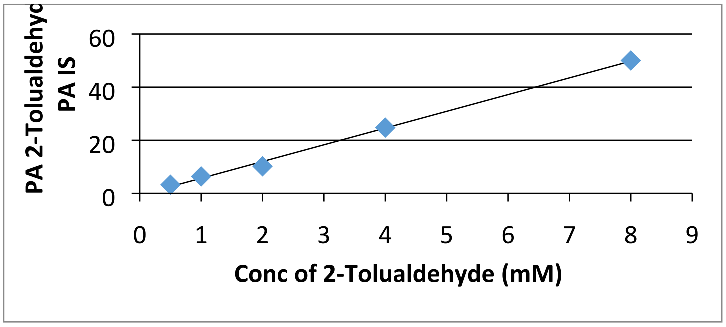

j) Calibration curve for 2-Fluorobenzaldehyde

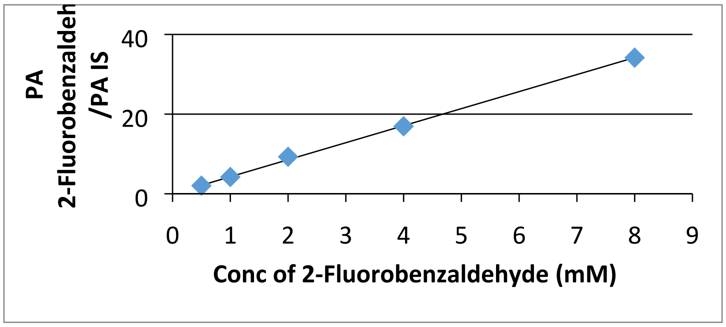

k) Calibration curve for 2-Nitrobenzaldehyde

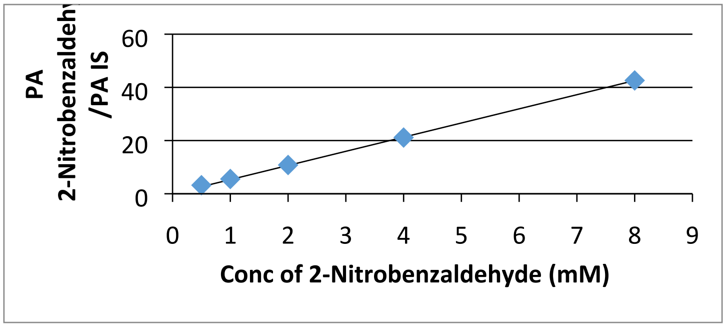

l) Calibration curve for 3-Nitrobenzaldehyde

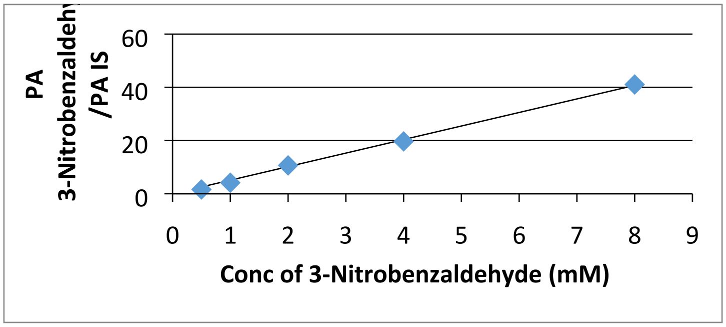

m) Calibration curve for 2,4-Difluorobenzaldehyde

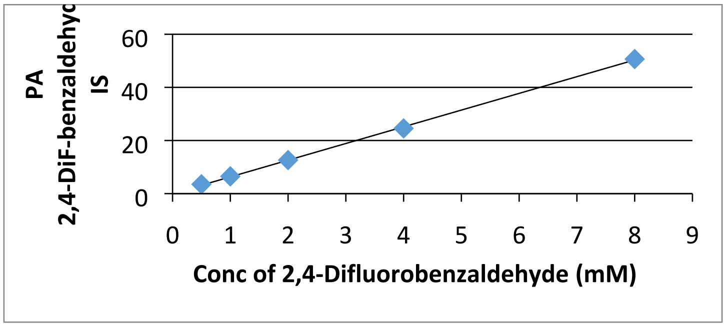

n) Calibration curve for 3,5-Dimethylbenzaldehyde

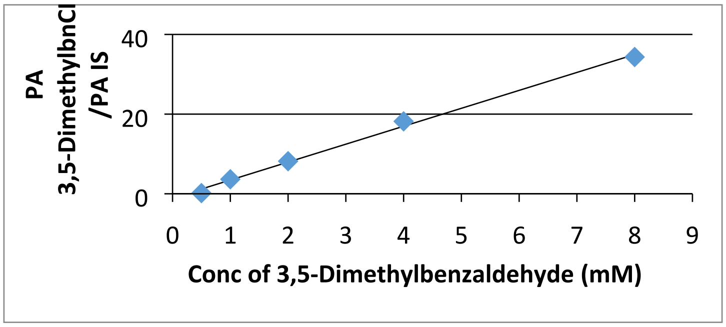

o) Calibration curve for 2,6-Dichlorobenzaldehyde

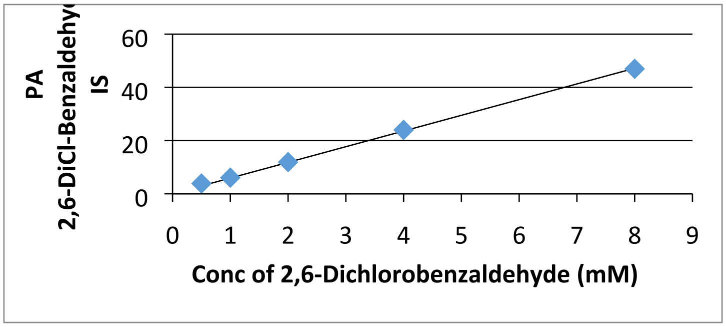

p) Calibration curve for Acetophenone

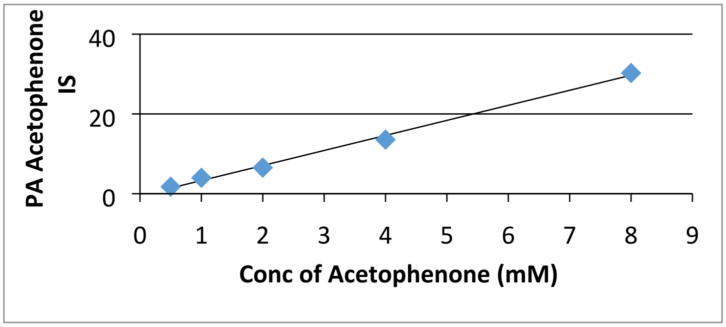

q) Calibration curve for 1-Naphthaldehyde

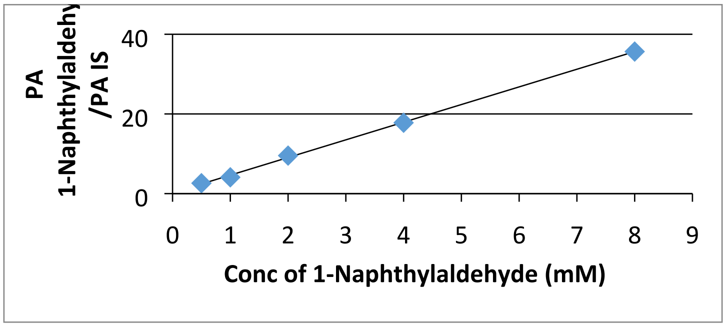

r) Calibration curve for Thiophene-2-carbaldehyde

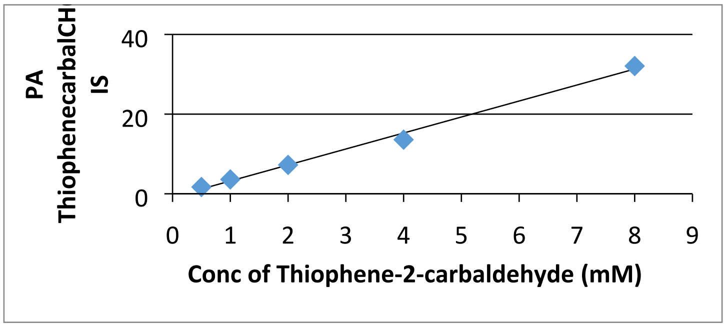

s) Calibration curve for Cinnamaldehyde

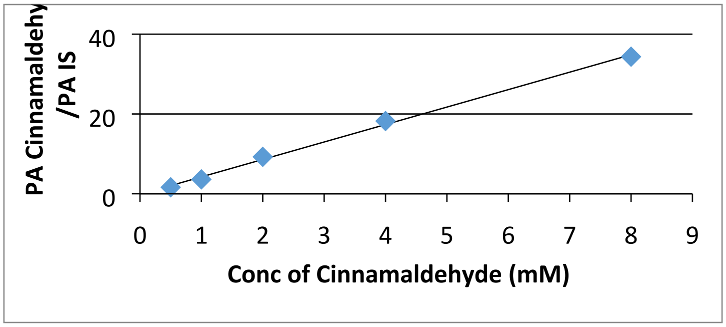

t) Calibration curve for 2-Phenylacetaldehyde

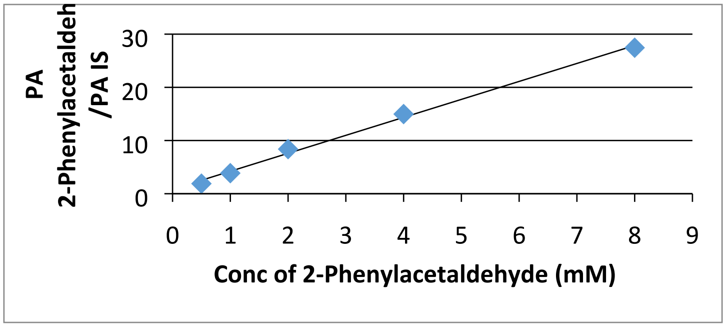

u) Calibration curve for Citral

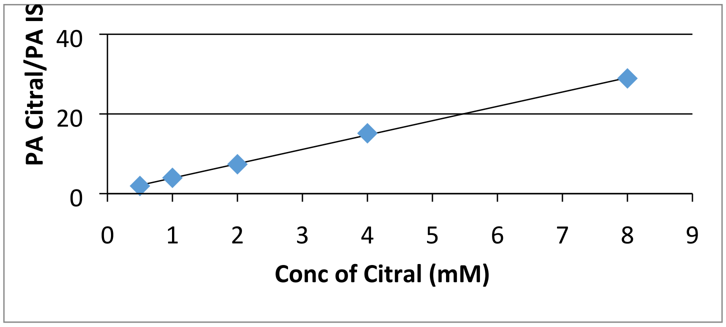

v) Calibration curve for 2,2,2-Trifluoro-1-phenylethan-1-one

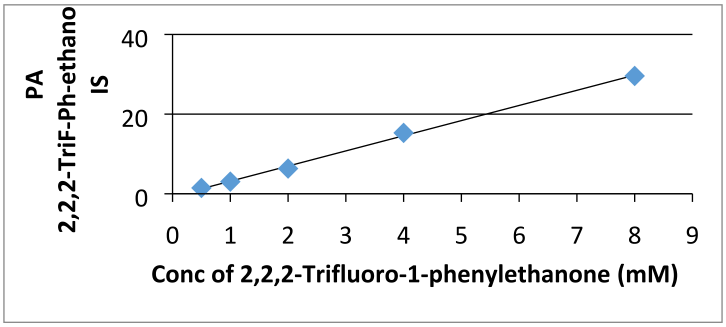

w) Calibration curve for Methyl 2-oxo-2-phenylacetate

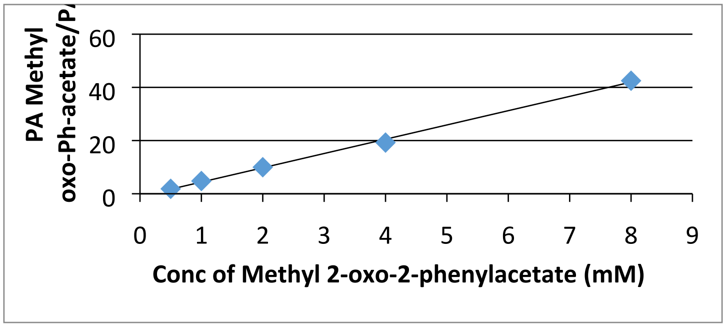

Figure S7. SDS-PAGE gel of purified Mb variants. The molecular weight of WT Mb is 18,408 Da.

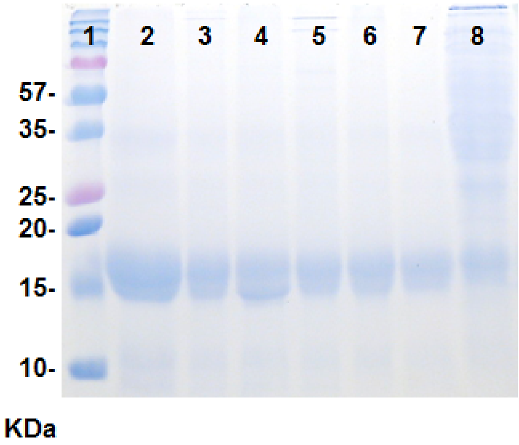

Lane 1: MW marker
Lane 2: WT Mb
Lane 3: Mb(L29A)
Lane 4: Mb(F43W)
Lane 5: Mb(F43V)
Lane 6: Mb(H64V)
Lane 7: Mb(V68A)
Lane 8: Mb(V68F)

Experimental Procedures:

Reagents and Analytical Methods. All the chemicals and reagents were purchased from commercial suppliers (Sigma-Aldrich, ACS Scientific, Acros, VWR Alfa Aesar) and used without any further purification, unless otherwise stated. ^{18}O-Water (Normalized, 97.4 atom %) for isotopic labeling experiments was purchased from Icon Isotopes. All dry reactions were carried out under argon atmosphere in oven-dried glassware with magnetic stirring using standard gas-light syringes, cannulae and septa. ^{1}H and ^{13}C NMR spectra were measured on Bruker DPX-400 (operating at 400 MHz for ^{1}H and 100 MHz for ^{13}C) or Bruker DPX-500 (operating at 500 MHz for ^{1}H and 125 MHz for ^{13}C). ^{19}F NMR spectra were measured on Bruker DPX-400 (operating at 376 MHz for ^{19}F). Tetramethylsilane (TMS) served as the internal standard (0 ppm) for ^{1}H NMR, $CDCl_3$ was used as the internal standard (77.0 ppm) for ^{13}C NMR and trichlorofluoromethane ($CFCl_3$) was used as the internal standard (0 ppm) for ^{19}F NMR. Silica gel chromatography purifications were carried out using AMD Silica Gel 60 230-400 mesh. Preparative thin layer chromatography was performed on TLC plates (Merck). Gas chromatography (GC) analyses were carried out using a Shimadzu GC-2010 gas chromatograph equipped with a FID detector and an Agilent J&W GC Chiral Cyclosil-B Column (30 m x 0.25 mm x 0.25 μm film). Separation method: 1 μL injection, injector temp.: 200 ºC, detector temp: 300 ºC. Gradient: column temperature set at 60 ºC for 0.10 min, then to 100 ºC at 60 ºC/min, to 200 ºC at 8 ºC/min, to 230 °C at 30 °C/min and then at 230 °C for 1.0 min. Total run time was 15.27 min. UV-Vis spectra were recorded on a Shimadzu UV-2401PC UV-VIS spectrophotometer, Wavelength Range (nm) from 700 to 300, Scan Speed: Fast, Sampling Interval (nm): 1.0, Scan Mode: Single.

Retention times (min):

Product	Retention time	Product	Retention time	Product	Retention time
**1**	5.621	**8a**	5.277	**15a**	13.511
**2a**	4.092	**8b**	3.625	**15b**	10.854
**2b**	4.723	**9a**	12.019	**16a**	6.127
**2c**	14.449	**9b**	10.367	**16b**	5.048
**3a**	6.795	**10a**	13.458	**17a**	3.998
**3b**	4.589	**10b**	10.979	**17b**	8.954
**4a**	9.434	**11a**	5.064	**18a**	7.564
**4b**	7.216	**11b**	3.225	**18b**	5.998
**5a**	13.831	**12a**	7.862	**19a**	7.435
**5b**	10.224	**12b**	5.126	**19b**	7.915
**6a**	6.236	**13a**	10.340	**20a**	5.756
**6b**	3.998	**13b**	9.274	**20b**	4.486
**7a**	6.742	**14a**	5.837	**21a**	8.581
**7b**	4.569	**14b**	5.170	**21b**	6.529

Cloning. The gene encoding for sperm whale myoglobin was amplified from plasmid pMYO (Addgene plasmid 34626) and cloned into the Nde I/Xho I cassette of plasmid pET22b (Novagen) to give pET22_MYO. The cloning of the Mb variants was described previously.[1]

Protein expression and purification. Wild-type Mb and the engineered Mb variants were expressed in *E. coli* BL21(DE3) cells as described previously.[1] Briefly, cells were grown in TB medium (ampicillin, 100 mg L^{-1}) at 37 °C (150 rpm) until OD_{600} reached 0.6. Cells were then induced with 0.25 mM β-d-1-thiogalactopyranoside (IPTG) and 0.3 mM δ-aminolevulinic acid (ALA). After induction, cultures were shaken at 150 rpm and 27 °C and harvested after 20 h by centrifugation at 4000 rpm at 4 °C. After cell lysis by sonication, the proteins were purified by Ni-affinity chromatography using the following buffers: loading buffer (50 mM Kpi, 800 mM NaCl, pH 7.0), wash buffer 1 (50 mM Kpi, 800 mM NaCl, pH 6.2), wash buffer 2 (50 mM Kpi, 800 mM NaCl, 250 mM glycine, pH 7.0) and elution buffer (50 mM Kpi, 800 mM NaCl, 300 mM L-histidine, pH 7.0). After buffer exchange (50 mM Kpi, pH 7.0), the proteins were stored at +4 °C. Protein concentration was determined using the CO-bound form of the hemoprotein and the extinction coefficient (ε_{422}) of 164 mM^{-1} cm^{-1}. Protein purity was confirmed by SDS-PAGE analysis under denaturing conditions (**Figure S7**).

Mb reactions. A typical reaction was carried out at a 400 μL scale in KPi buffer (50 mM) using 1 μM catalyst and and 10 mM sodium dithionite. A solution containing sodium dithionite (100 mM stock solution) in KPi buffer (50 mM, pH 7.0) was degassed by bubbling argon into the mixture for 3 min in a sealed vial. A separate vial containing the catalyst was carefully degassed in a similar manner. The solution was transferred to the catalyst-containing vial via cannulation. Reaction was initiated by addition of a 10 mM solution of the appropriate azide (200 mM stock solution in methanol, final percentage of methanol 5% v/v), with a syringe, and the reaction mixture was stirred for 24 h at 25 °C, under positive argon pressure.

Product analysis. The reactions were analyzed by adding 20 μL of internal standard (benzodioxole, 5 mM in methanol) to the reaction mixture, followed by extraction with 400 μL of dichloromethane and analyzed by GC-FID (see **Reagents and Analytical Methods** section for details on GC analyses). Calibration curves (see **Supplementary Figure S5**) for quantification of the different products were constructed using authentic standards produced synthetically as described in **Synthetic Procedures**, or purchased as described in **Reagents and Analytical Methods**. All measurements were performed at least in duplicate.

^{18}O labeling experiments. For the ^{18}O incorporation experiment, reactions were carried out at a 400 μL scale in KPi buffer (pH 7.0) containing 50% $H_2{}^{18}O$ using 1 μM Mb(H64V,V68A), 10 mM sodium dithionite, and 10 mM benzyl azide in methanol (5% v/v) under an argon atmosphere. The reactions were gently stirred 24 hours at 25 °C, then added of 20 μL of internal standard (benzodioxole, 5 mM in methanol). The products were extracted with 400 μL of dichloromethane and analyzed by GC-MS as described above.

Synthetic Procedures:

Synthesis of azides (*d_2*-1,3-21a). To a stirred solution of the appropriate benzyl bromide (5 mmol) in anhydrous DMF (10 mL) in argon atmosphere was added NaN_3 (7.5 mmol) at 25 °C. The reaction mixture was then stirred for 6h at 64 °C. After the reaction was complete, reaction mixture was quenched with water and extracted with Et_2O. Combined organic layers were concentrated *in vacuo* and the residue obtained was purified by flash chromatography on silica gel to obtain pure azides ***d_2*-1,3-21a** in variable yields.

Characterization Data:

(Azidomethyl-*d2*)benzene (*d_2*-1):

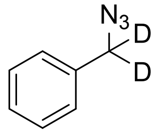

Following the standard procedure, yield (99%), R_f = 0.81 (in *n*-hexane); ^{1}H NMR (400 MHz, $CDCl_3$): δ 7.51-7.39(m, 5H); ^{13}C NMR (100 MHz, $CDCl_3$): δ 137.4, 128.8, 128.5, 128.2, 33.0.

(1-(Azidomethyl)-4-methylbenzene (3a):

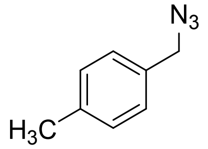

Following the standard procedure, yield 97%, R_f = 0.70 (in *n*-hexane); ^{1}H NMR (400 MHz, $CDCl_3$): δ 7.25-7.20 (m, 4H), 4.30 (s, 2H), 2.38 (s, 3H); ^{13}C NMR (100 MHz, $CDCl_3$): δ 138.1, 132.2, 129.4, 128.2, 54.7, 21.2.

1-(Azidomethyl)-4-methoxybenzene (4a):

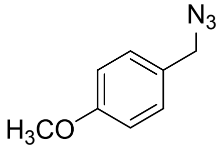

Following the standard procedure, yield 97%, R_f = 0.68 (in *n*-hexane); ^{1}H NMR (400 MHz, $CDCl_3$): δ 7.24 (d, 2H, *J* = 7.7 Hz), 6.91 (d, 2H, *J* = 7.8 Hz), 4.26 (s, 2H), 3.81 (s, 3H); ^{13}C NMR (100 MHz, $CDCl_3$): δ 159.5, 129.7, 127.3, 114.1, 55.2, 54.3.

1-(Azidomethyl)-4-nitrobenzene (5a):

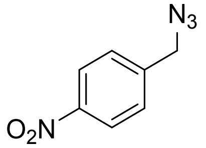

Following the standard procedure, yield 95%, R_f = 0.74 (in 20% EtOAc in *n*-hexane); ^{1}H NMR (400 MHz, $CDCl_3$): δ 8.14 (d, 2H, *J* = 7.9 Hz), 7.45 (d, 2H, *J* = 7.8 Hz), 4.47 (s, 2H); ^{13}C NMR (100 MHz, $CDCl_3$): δ 140.8, 128.7, 128.0, 126.3, 61.0, 31.5.

1-(Azidomethyl)-4-(trifluoromethyl)benzene (6a):

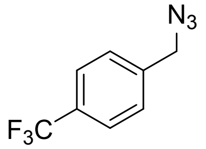

Following the standard procedure, yield 96.9%, R_f = 0.91 (in 10% EtOAc in *n*-hexane); ^{1}H NMR (500 MHz, $CDCl_3$): δ 7.65 (d, 2H, *J* = 7.5 Hz), 7.44 (d, 2H, J = 8 Hz), 4.43 (s, 2H); ^{13}C NMR (125 MHz, $CDCl_3$): δ 139.4, 128.2 (2C), 125.9, 125.8 (2C), 54.1; ^{19}F NMR (376 MHz, $CDCl_3$): δ -0.2.

1-(Azidomethyl)-2-methylbenzene (7a):

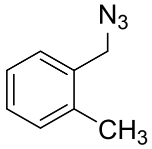

Following the standard procedure, yield 98%, R_f = 0.73 (in *n*-hexane); ^{1}H NMR (400 MHz, $CDCl_3$): δ 7.26-7.23 (m, 4H), 4.36 (s, 2H), 2.38 (s, 3H); ^{13}C NMR (100 MHz, $CDCl_3$): δ 136.8, 133.3, 130.6, 129.2, 128.6, 126.2, 52.9, 18.9.

1-(Azidomethyl)-2-fluorobenzene (8a):

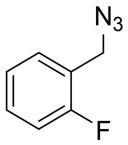

Following the standard procedure, yield 74.7%, R_f = 0.46 (in 10% EtOAc in *n*-hexane); ^{1}H NMR (500 MHz, $CDCl_3$): δ 7.36-7.32 (m, 2H), 7.17 (t, 1H, *J* = 7.5 Hz), 7.10 (t, 1H, *J* = 9.5 Hz), 4.41 (s, 2H); ^{13}C NMR (125 MHz, $CDCl_3$): δ 160.8 (d, *J* = 246.5 Hz), 130.4 (d, *J* = 3.7 Hz), 130.3 (d, *J* = 8.1 Hz), 124.4 (d, *J* = 3.6 Hz), 122.7 (d, J = 15.1 Hz), 115.6 (d, *J* = 21.0 Hz), 48.5 (d, *J* = 3.2 Hz); ^{19}F NMR (376 MHz, $CDCl_3$): δ -55.5;

1-(Azidomethyl)-2-nitrobenzene (9a):

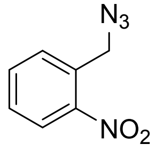

Following the standard procedure, yield 12.8%, R_f = 0.41 (in 10% EtOAc in *n*-hexane); ^{1}H NMR (500 MHz, $CDCl_3$): δ 8.12 (d, 1H, *J* = 8.0 Hz), 7.68-7.66 (m, 2H), 7.51 (t, 1H, *J* = 7.0 Hz), 4.85 (s, 2H); ^{13}C NMR (125 MHz, $CDCl_3$): δ 133.9, 131.6, 130.1, 129.0, 126.5, 51.9.

1-(Azidomethyl)-3-nitrobenzene (10a):

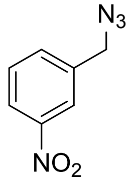

Following the standard procedure, yield 91.1%, R_f = 0.29 (in 10% EtOAc in *n*-hexane); ^{1}H NMR (500 MHz, $CDCl_3$): δ 8.20 (m, 2H), 7.67 (d, 1H, *J* = 7.5 Hz), 7.58 (t, 1H, *J* = 8.5 Hz), 4.50 (s, 2H); ^{13}C NMR (125 MHz, $CDCl_3$): δ 148.5, 137.6, 133.8, 129.9, 123.4, 122.8, 53.7.

1-(Azidomethyl)-2,4-difluorobenzene (11a):

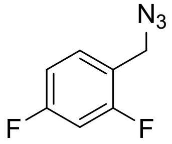

Following the standard procedure, yield 99%, R_f = 0.38 (in 10% EtOAc in *n*-hexane); ^{1}H NMR (500 MHz, $CDCl_3$): δ 7.34-7.29 (m, 1H) 6.92-6.85 (m, 2H), 4.37 (s, 2H); ^{13}C NMR (125 MHz, $CDCl_3$): δ 164.0 (d, *J* = 247.7 Hz), 161.0 (d, *J* = 248.6 Hz), 131.3 (d, *J* = 15.1 Hz), 118.7 (d, *J* = 15.5 Hz), 111.6 (d, *J* = 21.3 Hz), 104.2 (t, *J* = 25.1), 48.0 (d, J = 2.3 Hz); ^{19}F NMR (376 MHz, $CDCl_3$): δ -46.6, -51.0;

1-(Azidomethyl)-3,5-dimethylbenzene (12a):

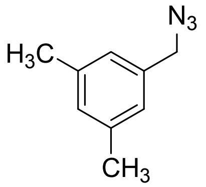

Following the standard procedure, yield 99%, R_f = 0.72 (in *n*-hexane); ^{1}H NMR (400 MHz, $CDCl_3$): δ 7.06 (s, 1H), 7.01 (s, 2H), 4.31 (s, 2H), 2.41(s, 3H); ^{13}C NMR (100 MHz, $CDCl_3$): δ 138.2, 129.1, 135.8, 129.9, 125.2, 54.6, 21.1.

2-(Azidomethyl)-1,3-dichlorobenzene (13a):

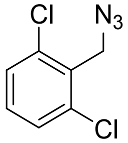

Following the standard procedure, yield 91.8%, R_f = 0.42 (in 10% EtOAc in *n*-hexane); ^{1}H NMR (500 MHz, $CDCl_3$): δ 7.37 (d, 2H, *J* = 8.0 Hz), 7.23 (t, 1H, *J* = 8 Hz), 4.68 (s, 2H); ^{13}C NMR (125 MHz, $CDCl_3$): δ 136.4 (2C), 131.5, 130.3, 128.5 (2C), 49.2.

(±)-(1-Azidoethyl)benzene (14a):

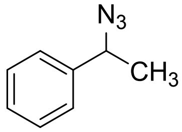

Following the standard procedure, yield 96%, R_f = 0.81 (in *n*-hexane); ^{1}H NMR (400 MHz, $CDCl_3$): δ 7.43-7.25 (m, 5H), 4.64 (q, 1H, *J* = 7.9 Hz), 1.57 (d, 3H, *J* = 8.2 Hz); ^{13}C NMR (100 MHz, $CDCl_3$): δ 140.8, 128.7, 128.0, 126.3, 61.0, 31.5.

1-(Azidomethyl)naphthalene (15a):

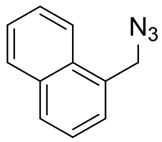

Following the standard procedure, yield 98%, R_f = 0.72 (in *n*-hexane); ^{1}H NMR (400 MHz, $CDCl_3$): δ 8.06-7.87 m, 3H), 7.61-7.47 (m, 4H), 4.77 (s, 2H); ^{13}C NMR (100 MHz, $CDCl_3$): δ 133.9, 131.3, 130.9, 129.4, 128.8, 127.2, 126.7, 126.1, 125.2, 123.4, 52.9.

2-(Azidomethyl)thiophene (16a):

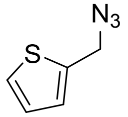

Following the standard procedure, yield 73%, R_f = 0.74 (in 15% EtOAc in *n*-hexane); ^{1}H NMR (400 MHz, $CDCl_3$): δ 7.32 (s, 1H), 7.04 (d, 2H, *J* = 7.8 Hz), 4.50 (s, 2H); ^{13}C NMR (100 MHz, $CDCl_3$): δ 137.1, 127.2, 127.0, 126.3, 48.9.

Cinnamyl azide (17a):

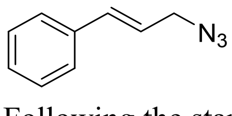

Following the standard procedure, yield 95.1%, R_f = 0.87 (in 10% EtOAc in *n*-hexane); ^{1}H NMR (500 MHz, $CDCl_3$): δ 7.41 (d, 2H, *J* = 7.5 Hz), 7.34 (t, 2H, *J* = 7.5 Hz), 7.27 (d, 1H, *J* = 10.5 Hz), 6.66 (d, 1H, *J* = 16 Hz), 6.26-6.23 (m, 1H), 3.95 (d, 2H, *J* = 6 Hz); ^{13}C NMR (125 MHz, $CDCl_3$): δ 136.0, 134.5, 128.6 (2C), 128.2, 126.6 (2C), 122.4, 53.0.

(2-Azidoethyl)benzene (18a):

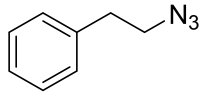

Following the standard procedure, yield 93%, R_f = 0.81(in 10% EtOAc in *n*-hexane); ^{1}H NMR (400 MHz, $CDCl_3$): δ 7.46-7.32 (m, 5H), 3.55 (t, 2H, *J* = 7.9 Hz), 2.97 (t, 2H, *J* = 7.8 Hz); ^{13}C NMR (100 MHz, $CDCl_3$): δ 137.7, 128.4, 128.2, 126.4, 51.9, 34.9.

Geranyl azide (19a):

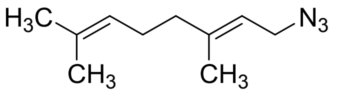

Following the standard procedure, yield 74.5%, R_f = 0.51 (in 100% *n*-hexane); ^{1}H NMR (500 MHz, $CDCl_3$): δ 5.33 (t, 1H, *J* = 7.5 Hz), 5.09 (t, 1H, *J* = 5.5 Hz), 3.76 (t, 2H, J = 9 Hz), 2.13-2.10 (m, 4H), 1.70 (s, 3H), 1.68 (s, 2H), 1.61 (s, 3H); ^{13}C NMR (125 MHz, $CDCl_3$): δ 143.1, 131.9, 123.5, 117.9, 48.0, 39.5, 26.3, 25.6, 17.7, 16.4.

(±)-(1-Azido-2,2,2-trifluoroethyl)benzene (20a):

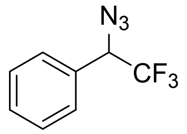

Following the standard procedure, yield 78.8%, R_f = 0.81 (in 10% EtOAc in *n*-hexane); ^{1}H NMR (500 MHz, $CDCl_3$): δ 7.40-7.32 (m, 5H), 4.64-4.60 (m, 1H), 1.54 (d, 3H, *J* = 7 Hz); ^{13}C NMR (125 MHz, $CDCl_3$): δ 140.8, 128.7 (2C), 128.1, 126.3 (2C), 61.1, 21.5.

(±)-Methyl 2-azido-2-phenylacetate (21a):

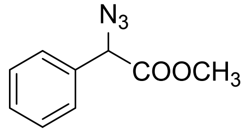

Following the standard procedure, yield 2.1%, R_f = 0.19 (in 33% DCM in *n*-hexane); ^{1}H NMR (500 MHz, $CDCl_3$): δ 8.01 (d, 2H, *J* = 8 Hz), 7.66 (t, 1H, *J* = 7.5 Hz), 7.51 (t, 2H, *J* = 8 Hz), 7.28-7.26 (m, 1H), 3.98 (s, 3H); ^{13}C NMR (125 MHz, $CDCl_3$): δ 164.0, 133.9, 129,2, 128.5, 127.1,126.9, 52.0.

Synthesis of Citral (22): to a stirred solution of pyridinium chlorocromate (419 mg, 1.9 mmol) in anhydrous dichlorometane (5 mL) under argon atmosphere at 25 °C was added a solution of commercially available geraniol (200 mg, 1.3 mmol) in anhydrous dichloromethane (5 mL) and the mixture was skept in agitation for 3h at 25 °C. After monitoring the consumption of starting material by TLC, the reaction mixture was directly evaporates under reduced pressure and purified by flash chromatography on silica gel (eluting mixture 10% EtOAc in *n*-Hexane) to obtain the desired product as colorless oil (yield 91%, 180 mg, 65/35 trans/cis mixture according by ^{1}H-NMR).

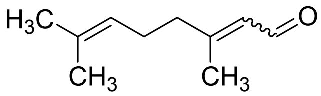

R_f = 0.84 (in 10% EtOAc in *n*-hexane); ^{1}H NMR (500 MHz, $CDCl_3$): δ 9.95 (d, 1H, *J* = 10.5 Hz, trans), 9.85 (d, 1H, *J* = 8.5 Hz, cis), 5.84 (d, 1H, *J* = 8.0 Hz), 5-06-5.03 (m, 1H, trans/cis), 2.20-2.15 (m, 4H), 2.15 (s, 3H), 1.64 (s, 3H), 1.57 (s, 3H, trans), 1.55 (s, 3H, cis); ^{13}C NMR (125 MHz, $CDCl_3$): δ 191.2 (trans), 190.7 (cis), 163.7 (trans/cis), 133.6 (cis), 132.8 (trans), 128.6 (cis), 127.3 (trans), 122.5 (trans), 122.2 (cis), 32.5 (trans/cis), 27.0 (trans/cis), 25.7 (cis), 25.6 (trans), 17.6 (trans/cis), 17.5 (trans/cis).

Synthesis of *N*-benzyl-1-phenylmethanimine (2c): to a stirred solution of commercially available benzaldehyde (509 μL, 4.7 mmol) and benzyl amine (472 μL, 4.7 mmol) in anhydrous methanol (10 mL) were added 3 drops of glacial acetic acid and the reaction mixture was stirred at 25 °C for 12h. After monitoring the total consumption of the starting material, the methanol was evaporated under reduced pressure and the crude product was purified by flash chromatography on silica gel (eluting mixture 100% dichloromethane) to obtain the desired product as a colorless oil (yield 92%, 838 mg).

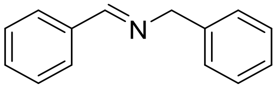

R_f = 0.84 (in 100% in DCM); ^{1}H NMR (500 MHz, $CDCl_3$): δ 8.40 (s, 1H), 7.80-7.78 (m, 2H), 7.43 (s, 3H), 7.36-7.35 (m, 4H), 7.27-7.26 (m, 1H), 4.83 (s, 2H); ^{13}C NMR (125 MHz, $CDCl_3$): δ 161.9, 130.7, 129.7, 129.0, 128.6 (2C), 128.5 (2C), 128.2 (2C), 127.9 (2C), 127.0, 65.0.

References:

[1] M. Bordeaux, V. Tyagi, R. Fasan, *Angew. Chem. Int. Ed.* 2015, *54*, 1744–1748.

[2] M. Bordeaux, R. Singh, R. Fasan, *Bioorg. Med. Chem.* 2014, *22*, 5697-5704.

Spectra:

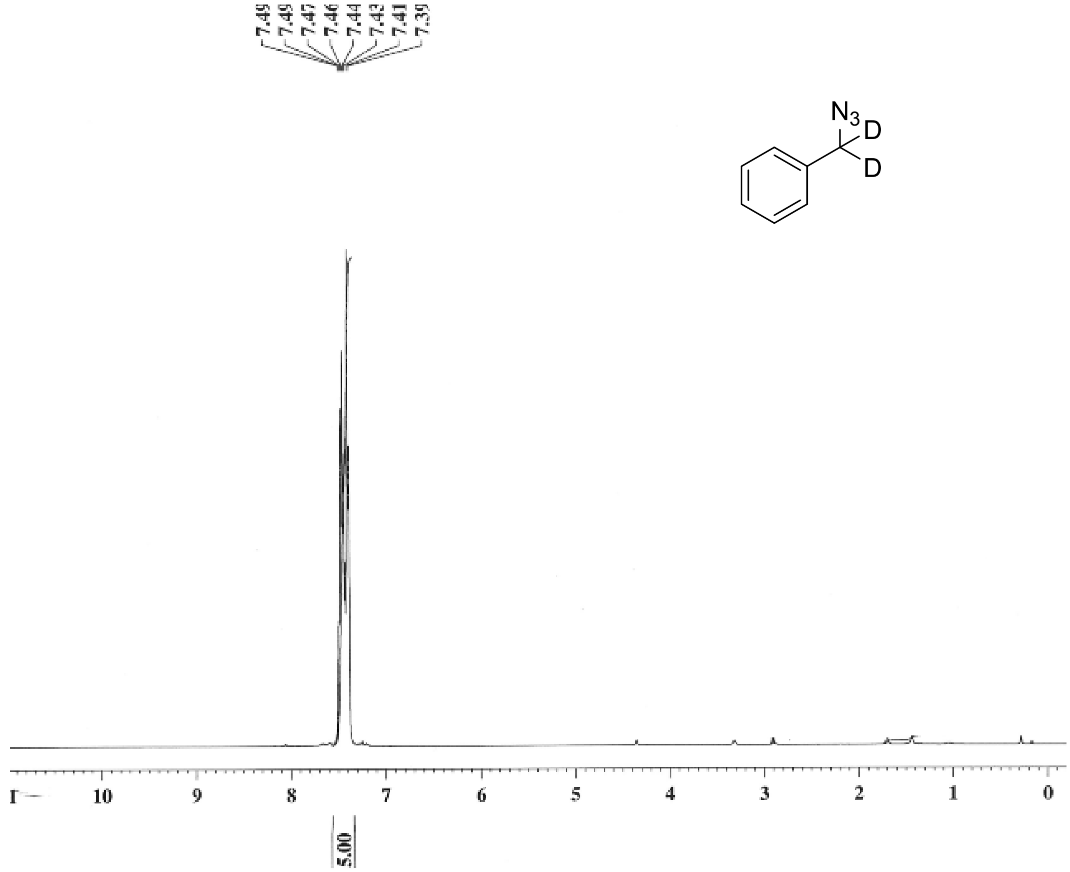
7.49
7.49
7.47
7.46
7.44
7.43
7.41
7.39
N3
D
D
5.00

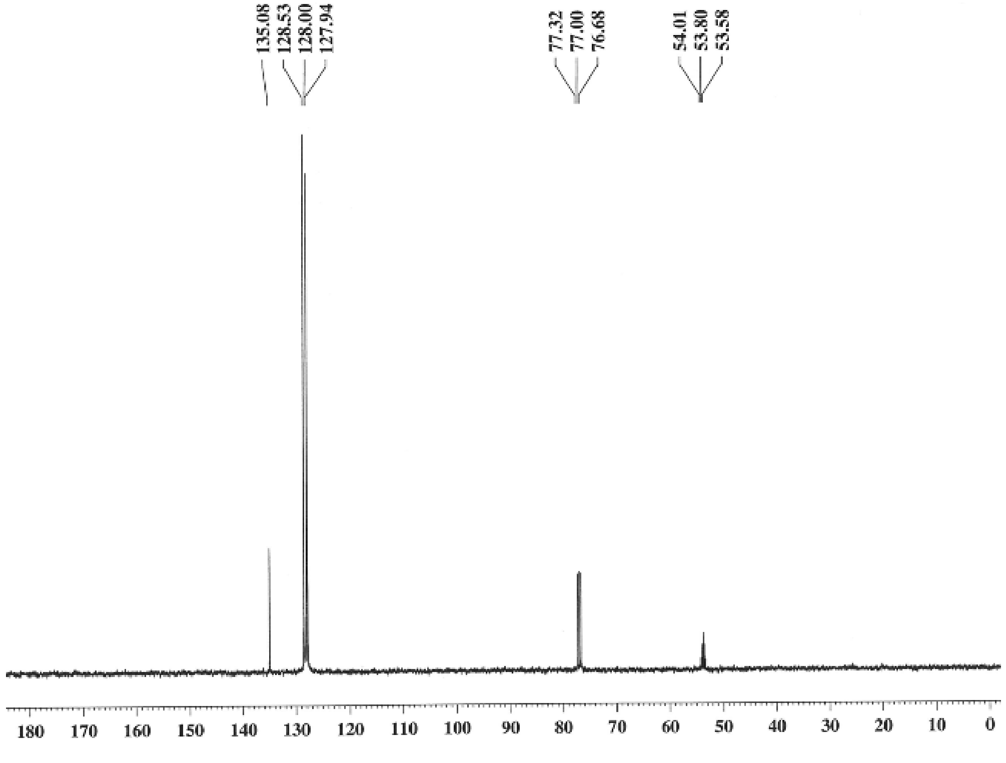
135.08
128.53
128.00
127.94
77.32
77.00
76.68
54.01
53.80
53.58

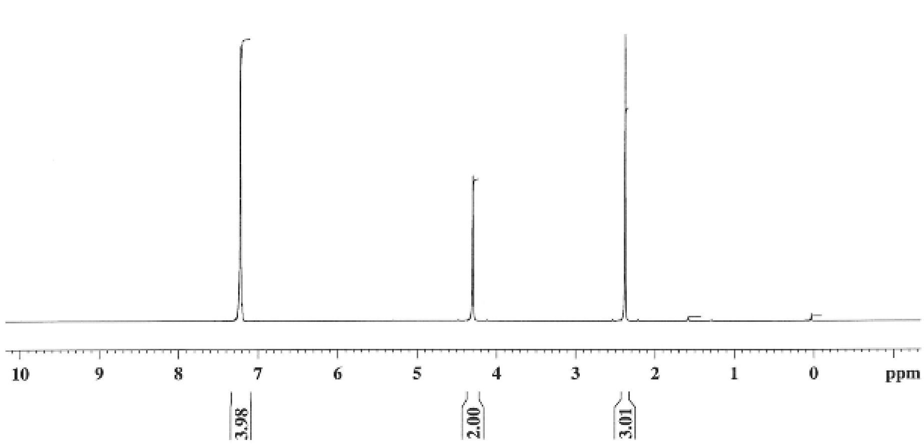
7.25
7.22
7.20
4.30
2.38
10
9
8
7
6
5
4
3
2
1
0
ppm
3.98
2.00
3.01

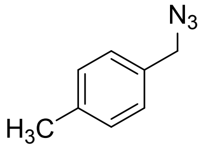
N3
H3C

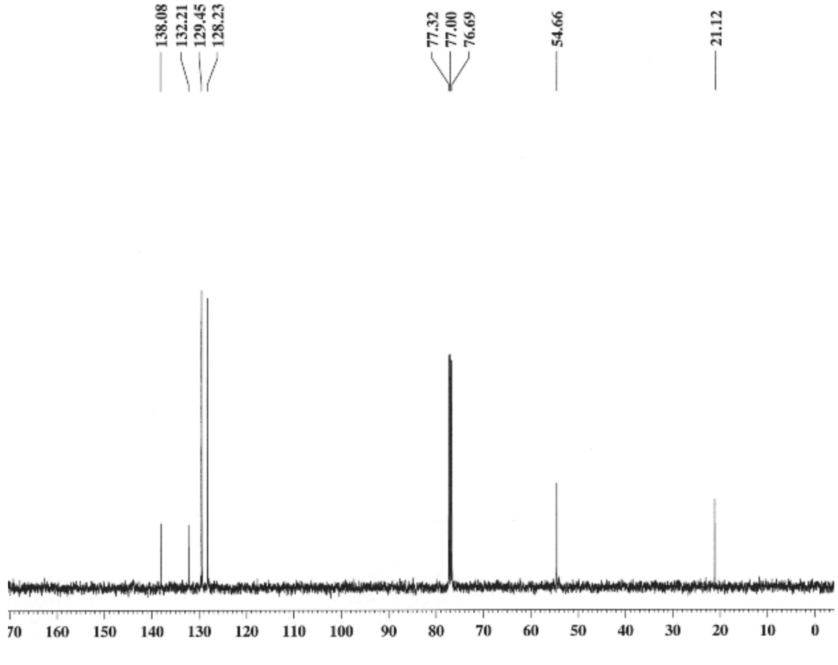
138.08
132.21
129.45
128.23
77.32
77.00
76.69
54.66
21.12
70
160
150
140
130
120
110
100
90
80
70
60
50
40
30
20
10
0

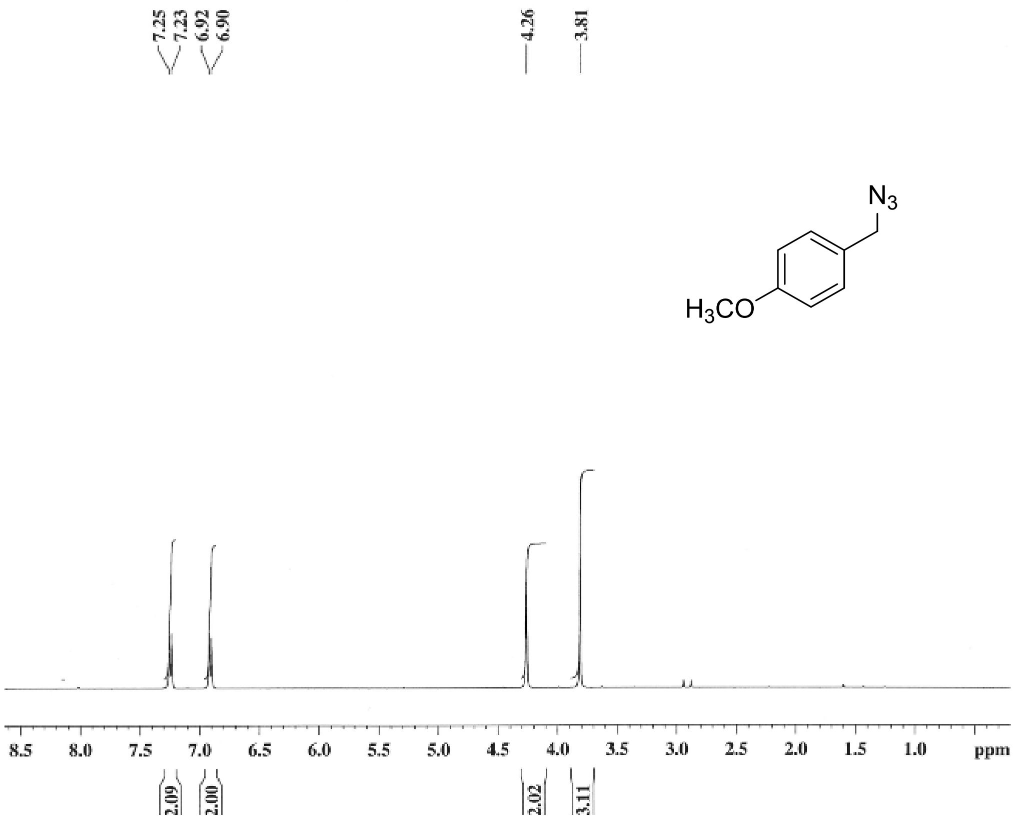
7.25
7.23
6.92
6.90
4.26
3.81
8.5 8.0 7.5 7.0 6.5 6.0 5.5 5.0 4.5 4.0 3.5 3.0 2.5 2.0 1.5 1.0 ppm
2.09
2.00
2.02
3.11
N3
H3CO

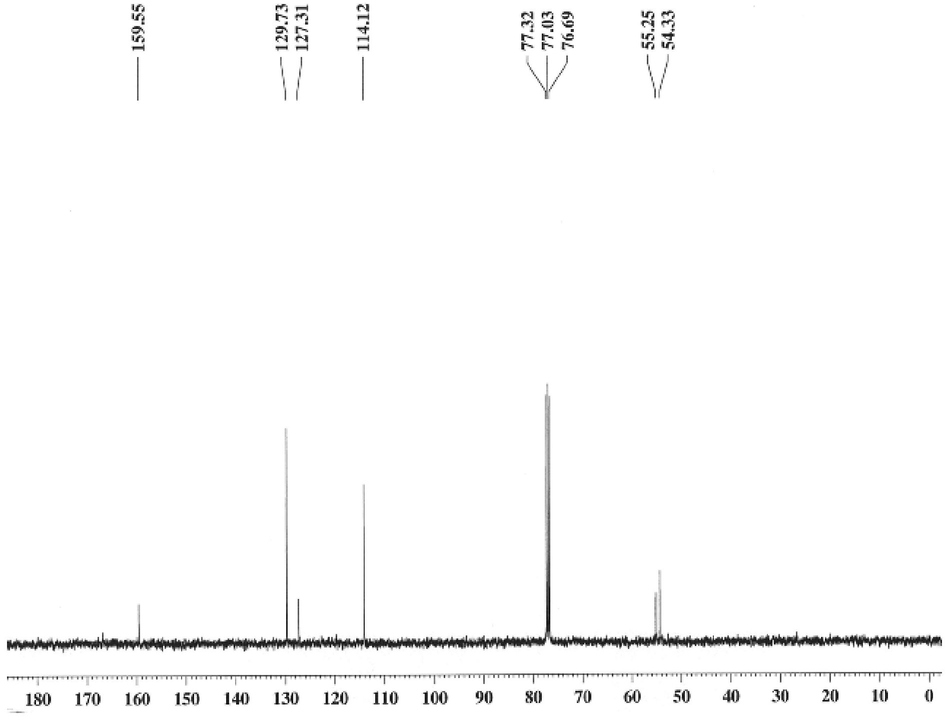
159.55
129.73
127.31
114.12
77.32
77.03
76.69
55.25
54.33
180 170 160 150 140 130 120 110 100 90 80 70 60 50 40 30 20 10 0

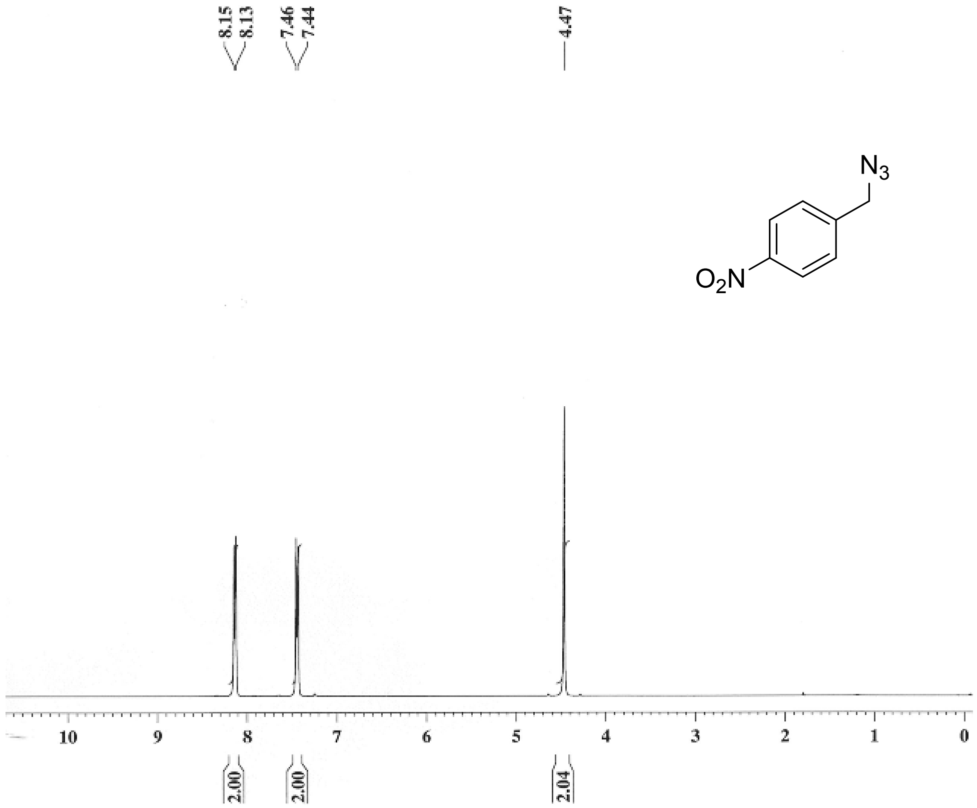
8.15
8.13
7.46
7.44
4.47
N_3
O_2N
10 9 8 7 6 5 4 3 2 1 0
2.00
2.00
2.04

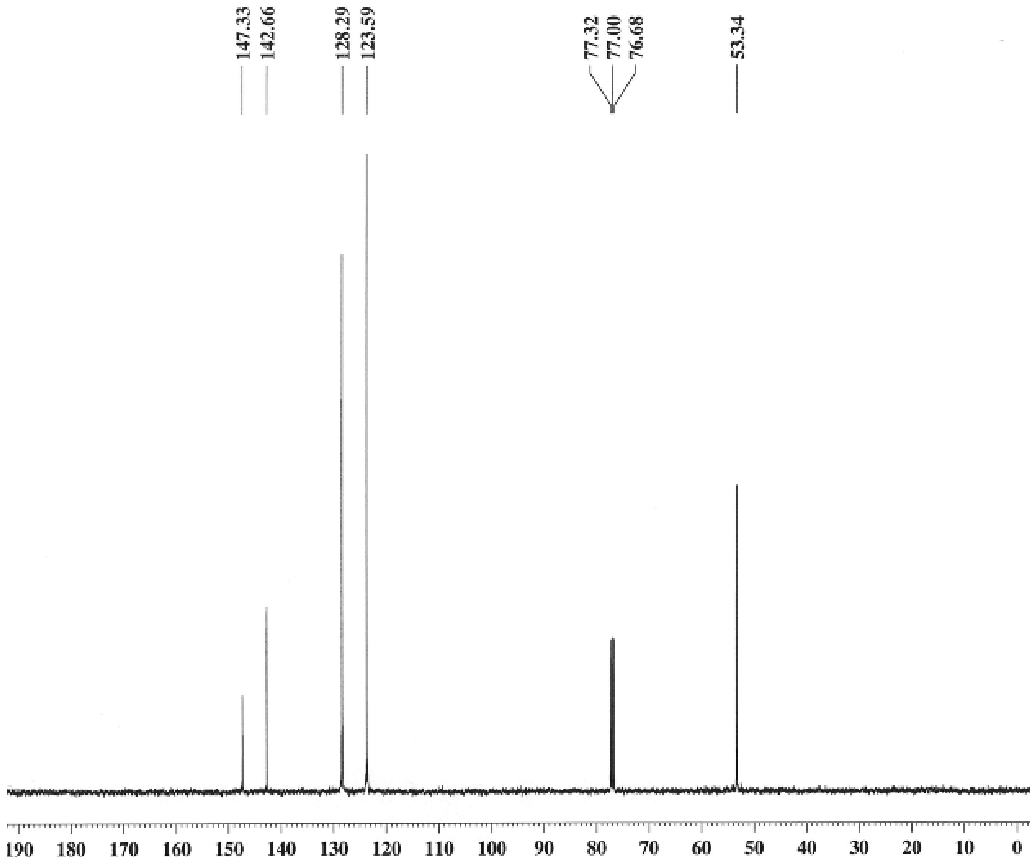
147.33
142.66
128.29
123.59
77.32
77.00
76.68
53.34
190 180 170 160 150 140 130 120 110 100 90 80 70 60 50 40 30 20 10 0

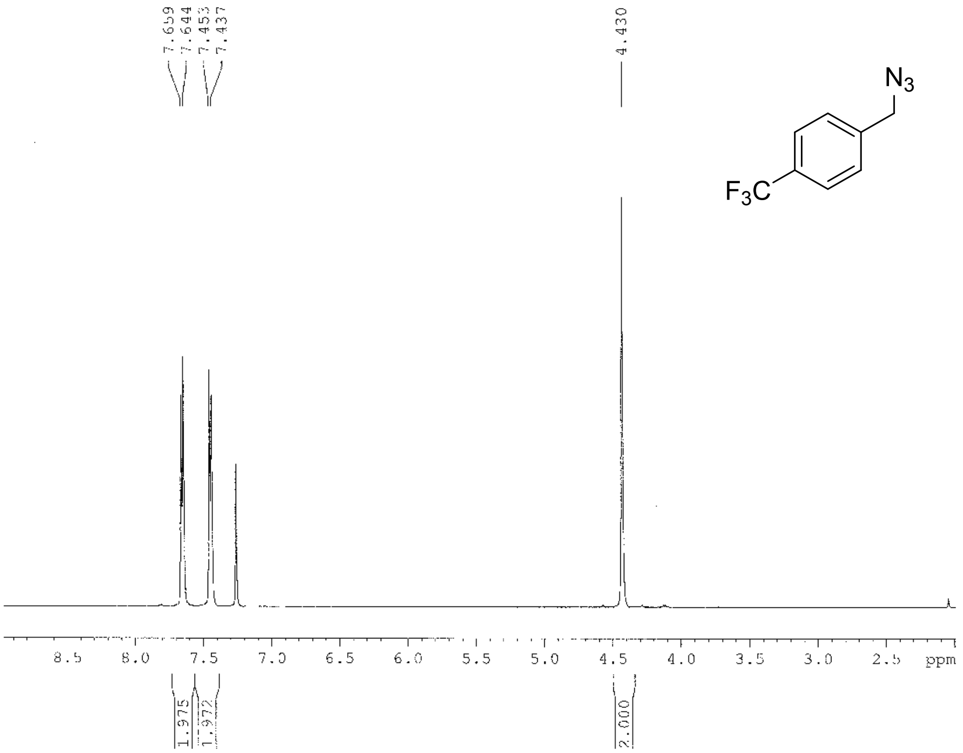
7.659
7.644
7.453
7.437
4.430
N3
F3C
8.5 8.0 7.5 7.0 6.5 6.0 5.5 5.0 4.5 4.0 3.5 3.0 2.5 ppm
1.975
1.972
2.000

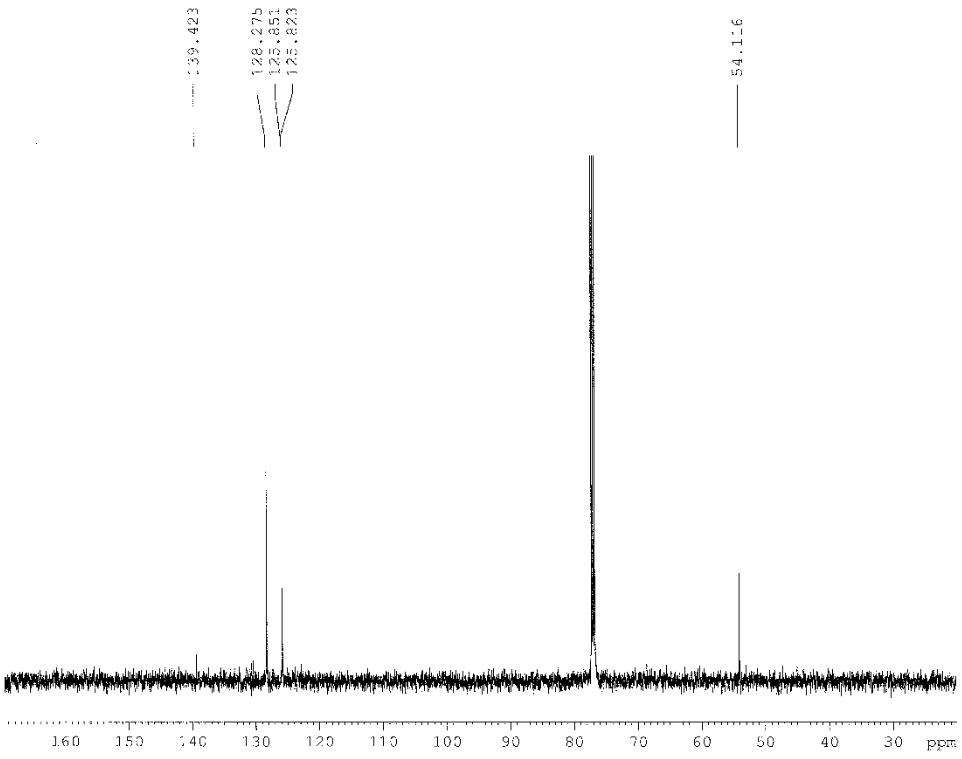
139.423
128.275
125.851
125.823
54.116
160 150 140 130 120 110 100 90 80 70 60 50 40 30 ppm

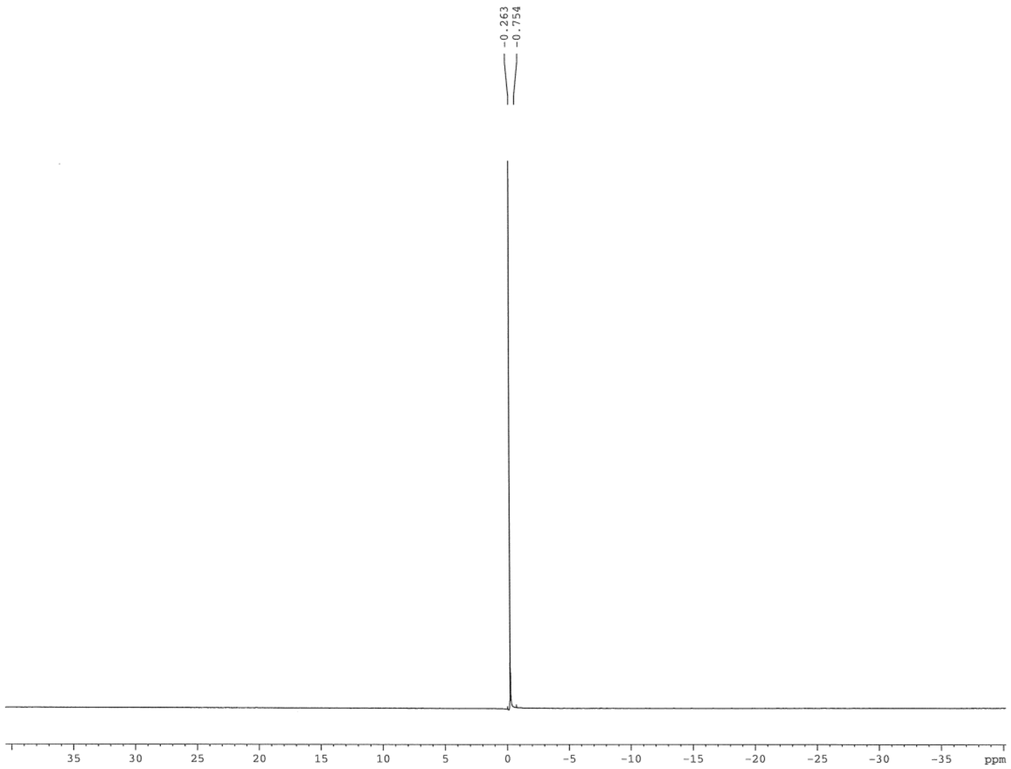
-0.263
-0.754
35 30 25 20 15 10 5 0 -5 -10 -15 -20 -25 -30 -35 ppm

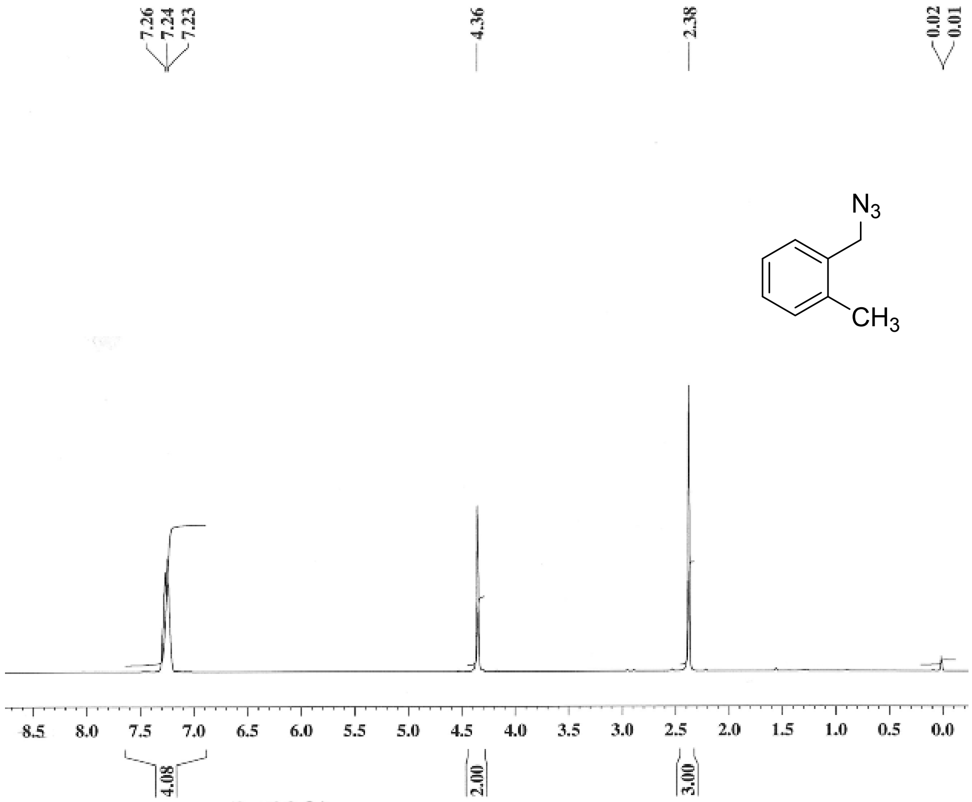
7.26
7.24
7.23
4.36
2.38
0.02
0.01
N3
CH3
8.5 8.0 7.5 7.0 6.5 6.0 5.5 5.0 4.5 4.0 3.5 3.0 2.5 2.0 1.5 1.0 0.5 0.0
4.08
2.00
3.00

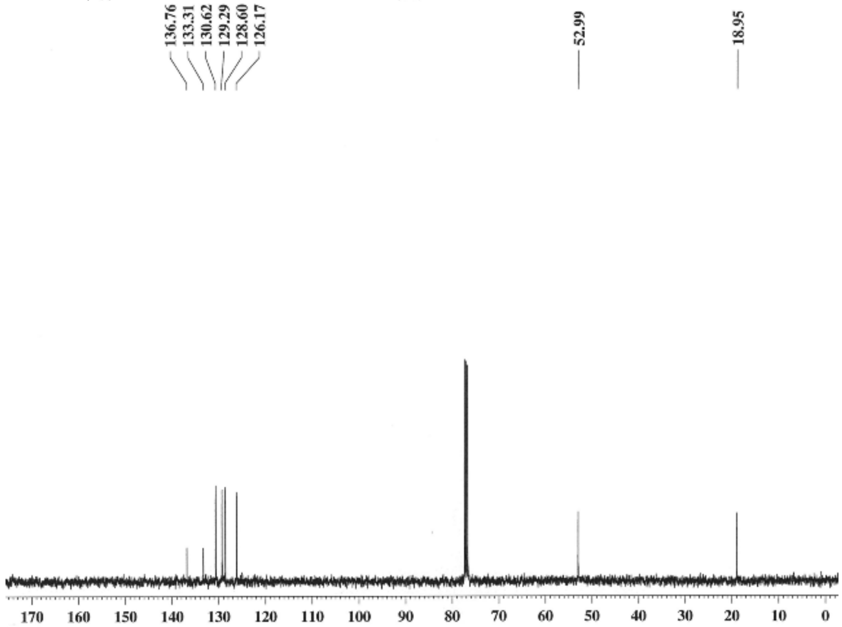
136.76
133.31
130.62
129.29
128.60
126.17
52.99
18.95
170 160 150 140 130 120 110 100 90 80 70 60 50 40 30 20 10 0

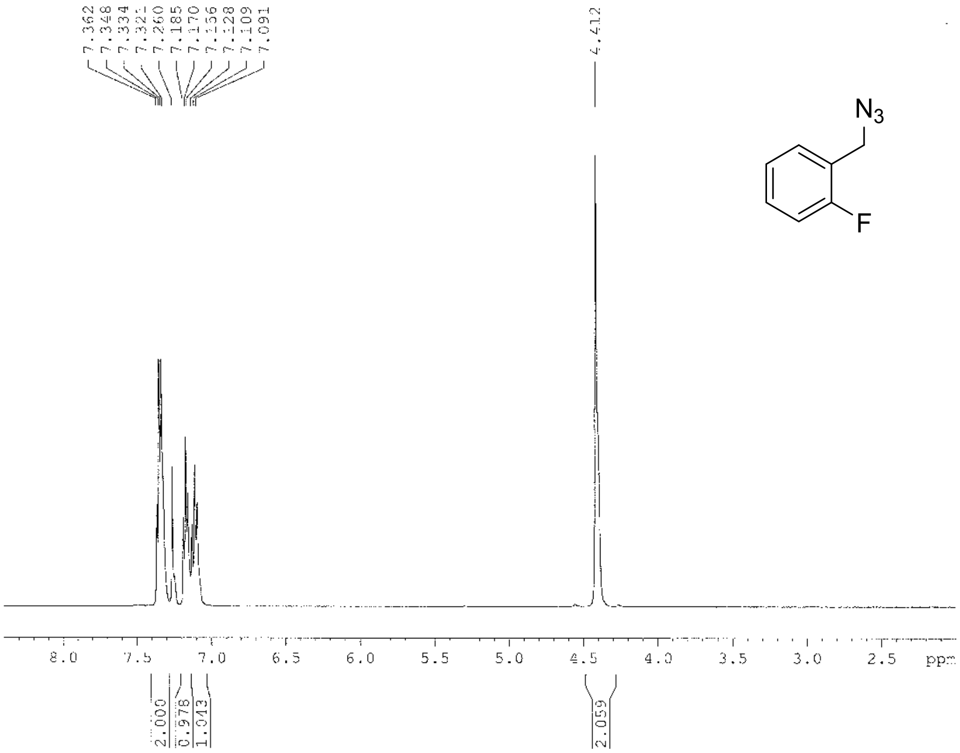
N3
F
7.362
7.348
7.334
7.260
7.185
7.170
7.156
7.128
7.109
7.091
4.412
8.0 7.5 7.0 6.5 6.0 5.5 5.0 4.5 4.0 3.5 3.0 2.5 ppm
2.000
0.978
1.043
2.059

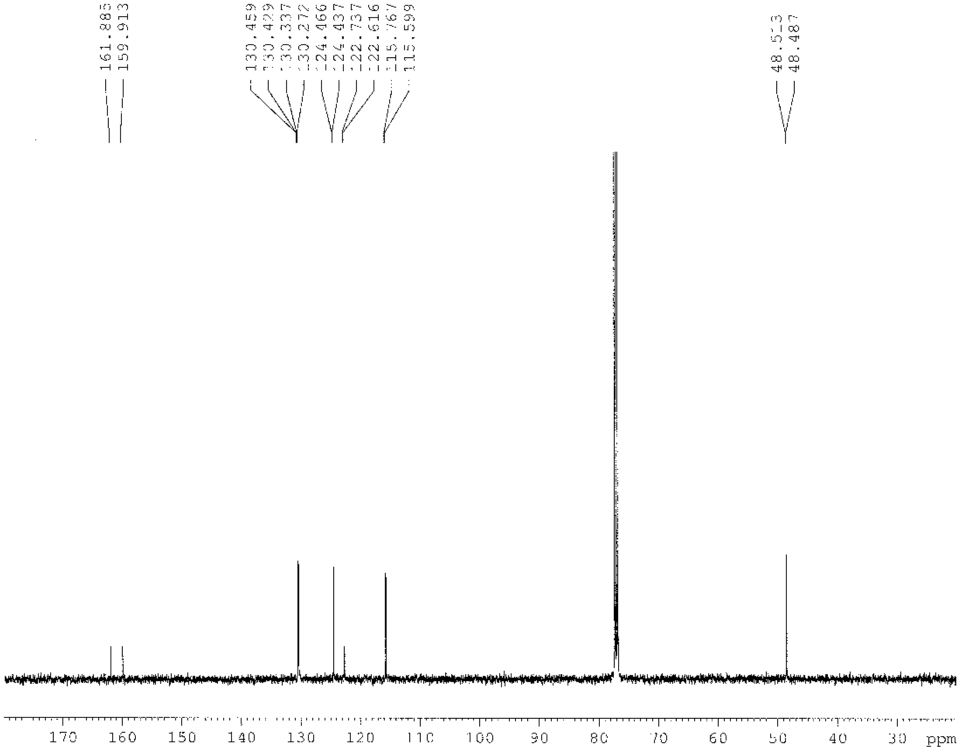
159.913
130.459
48.487
170 160 150 140 130 120 110 100 90 80 70 60 50 40 30 ppm

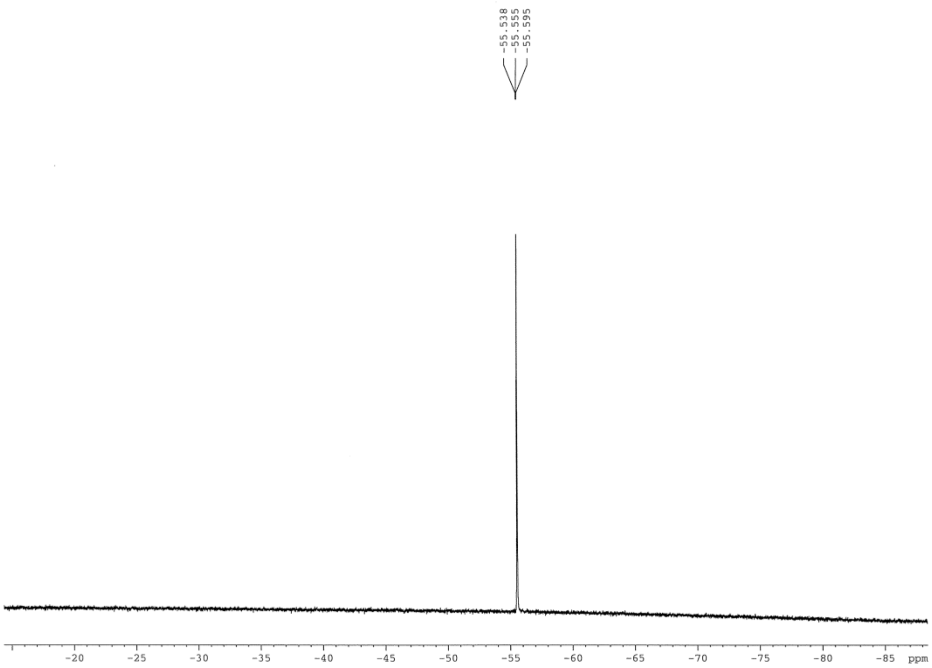
-55.538
-55.555
-55.595
-20
-25
-30
-35
-40
-45
-50
-55
-60
-65
-70
-75
-80
-85
ppm

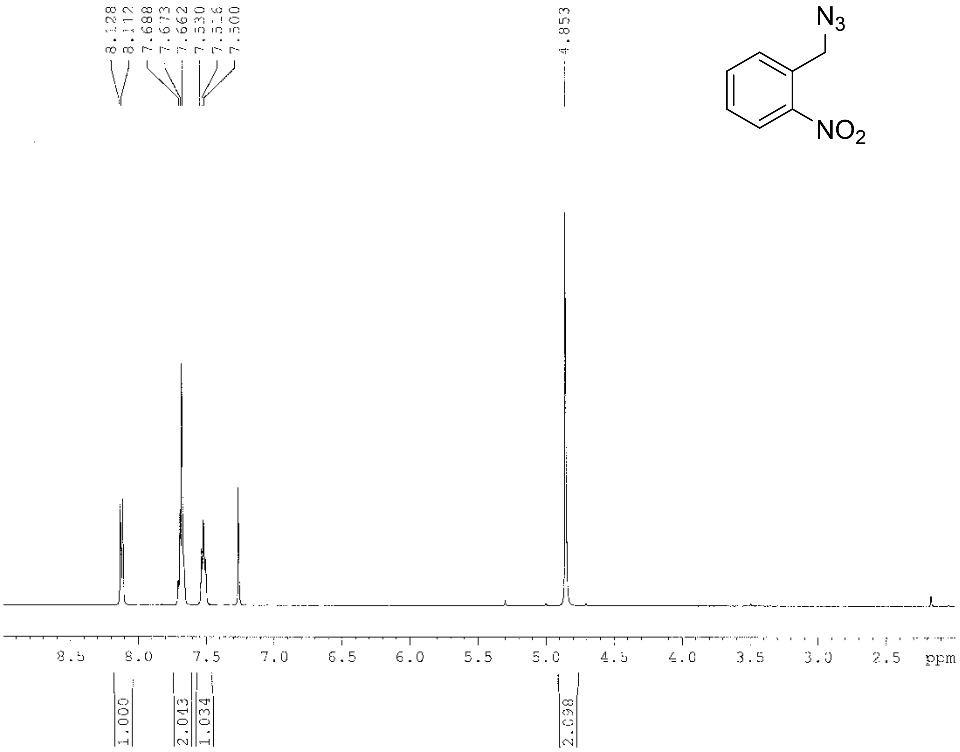
8.128
8.112
7.688
7.673
7.662
7.530
7.516
7.500
4.853
N_3
NO_2
8.5 8.0 7.5 7.0 6.5 6.0 5.5 5.0 4.5 4.0 3.5 3.0 2.5 ppm
1.000
2.043
1.034
2.098

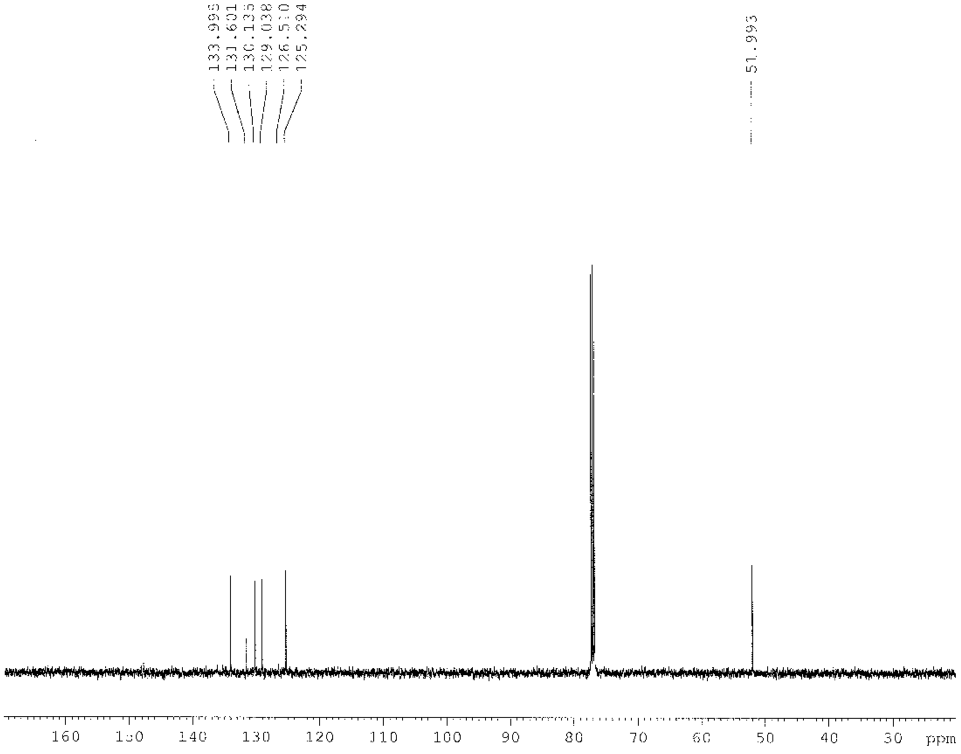
133.995
131.601
130.135
129.038
126.510
125.294
51.993
160 150 140 130 120 110 100 90 80 70 60 50 40 30 ppm

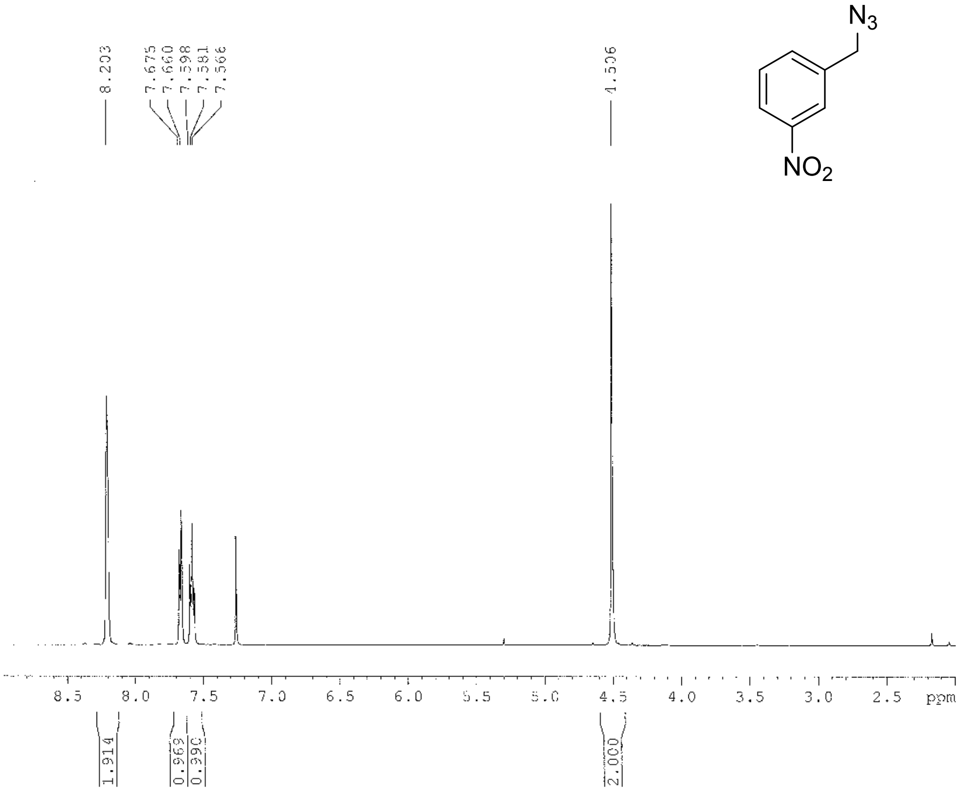
N3
NO2
8.203
7.675
7.660
7.598
7.581
7.566
4.506
8.5 8.0 7.5 7.0 6.5 6.0 5.5 5.0 4.5 4.0 3.5 3.0 2.5 ppm
1.914
0.969
0.390
2.000

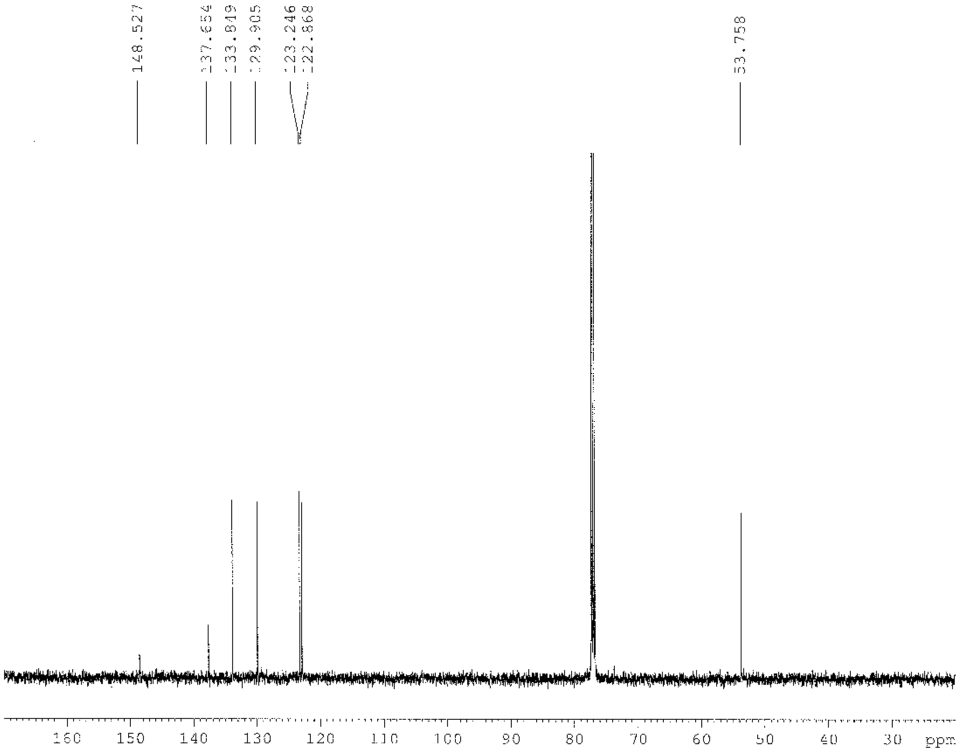
148.527
137.654
133.849
129.905
123.246
122.868
53.758
160 150 140 130 120 110 100 90 80 70 60 50 40 30 ppm

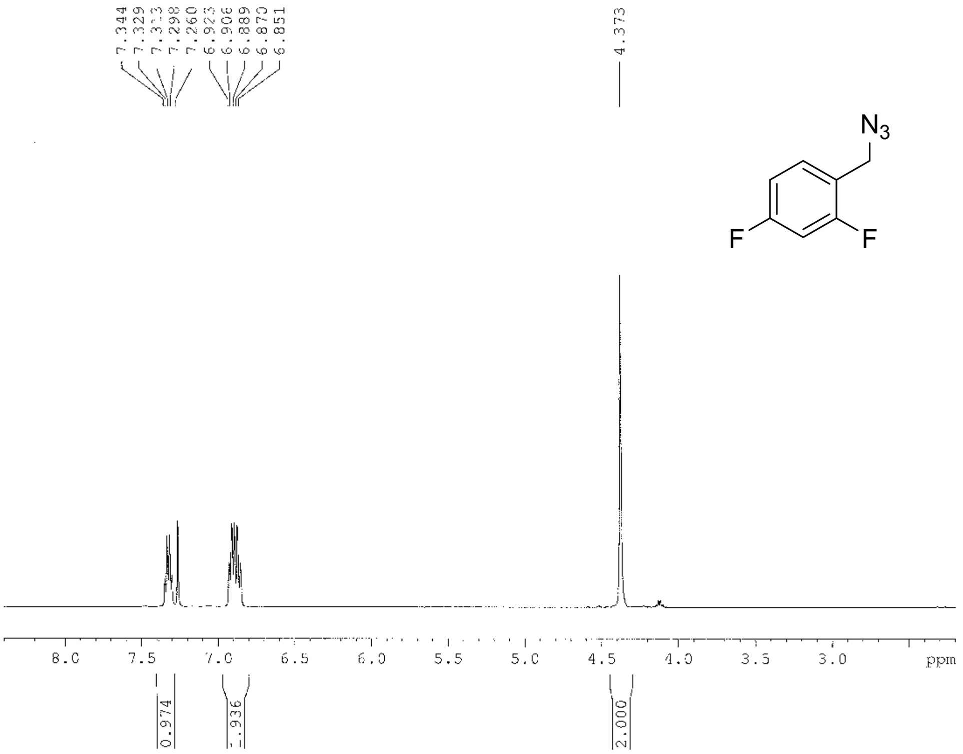
7.344
7.329
7.298
7.260
6.906
6.889
6.870
6.851
4.373
N3
F
F
ppm
2.000

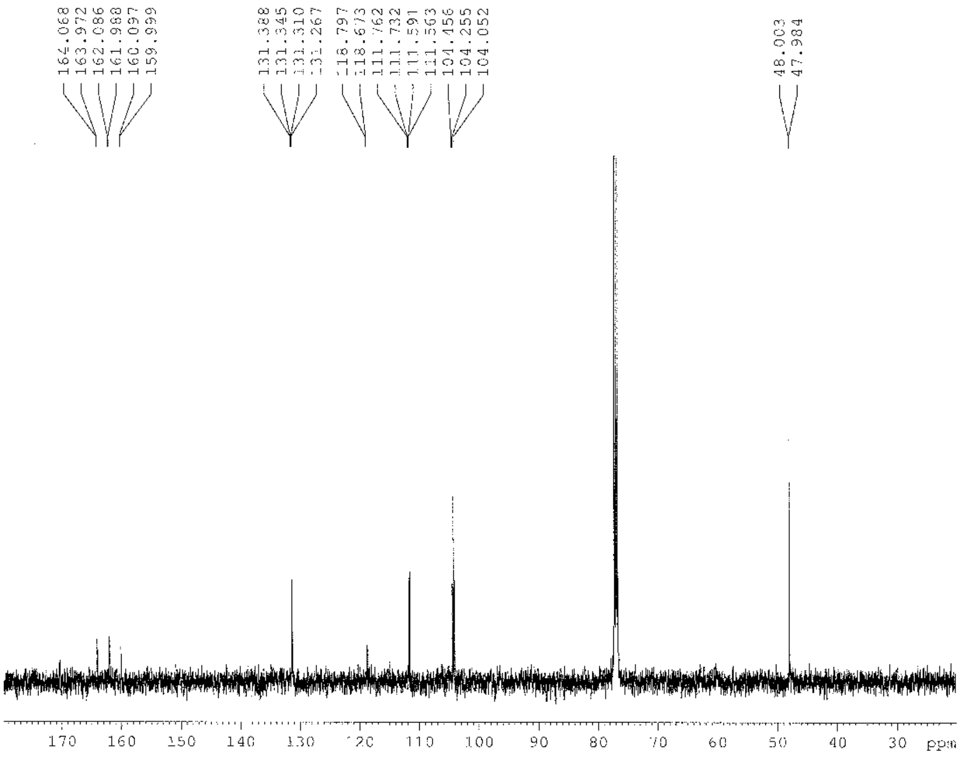
164.068
163.972
162.086
161.988
160.097
159.999
131.388
131.345
131.310
118.673
111.762
111.732
111.591
111.563
104.456
104.255
104.052
48.003
47.984
ppm

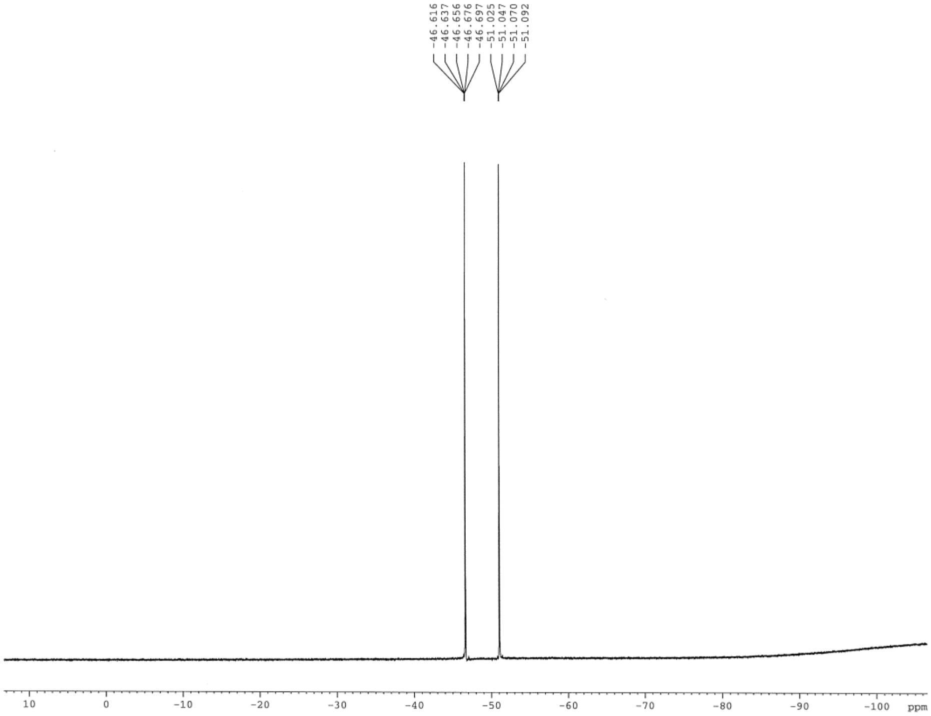

-46.616
-46.637
-46.656
-46.676
-46.697
-51.025
-51.047
-51.070
-51.092
10
0
-10
-20
-30
-40
-50
-60
-70
-80
-90
-100
ppm

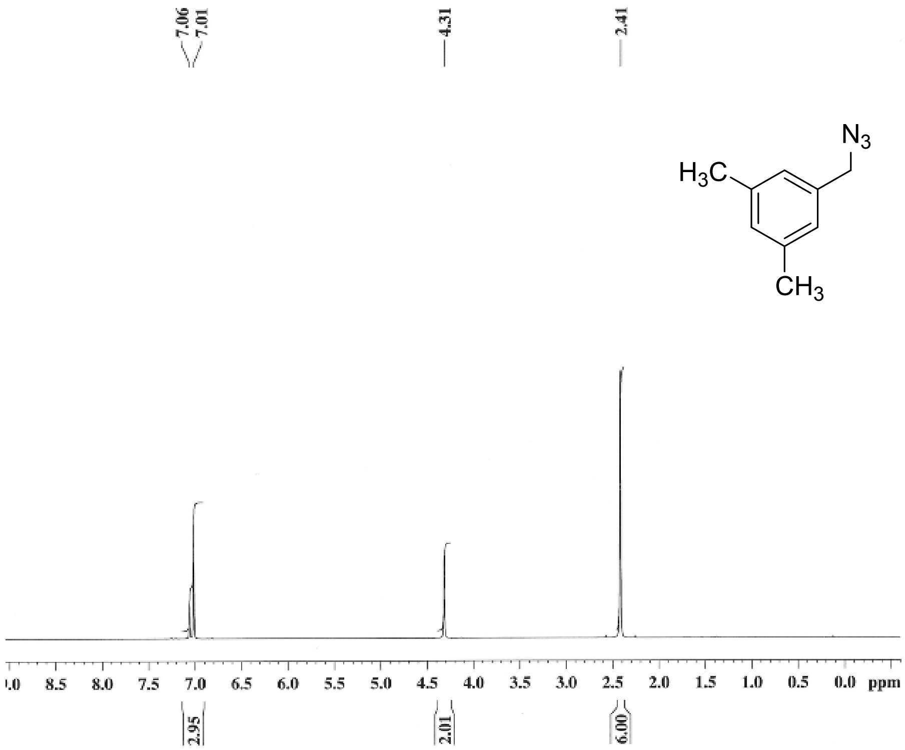
7.06
7.01
4.31
2.41
N3
H3C
CH3
ppm
2.95
2.01
6.00

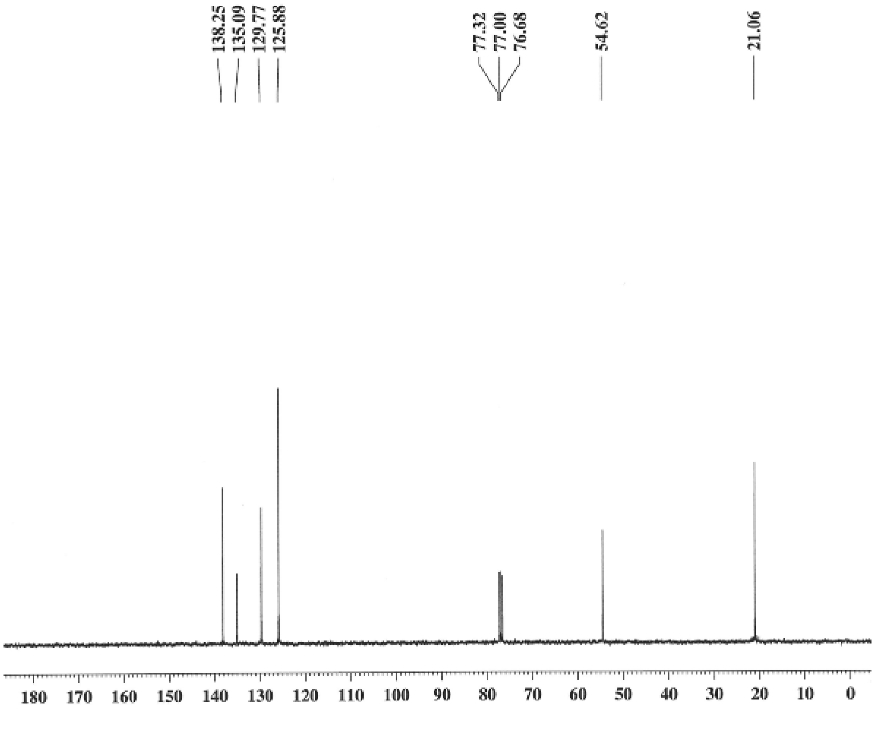
138.25
135.09
129.77
125.88
77.32
77.00
76.68
54.62
21.06

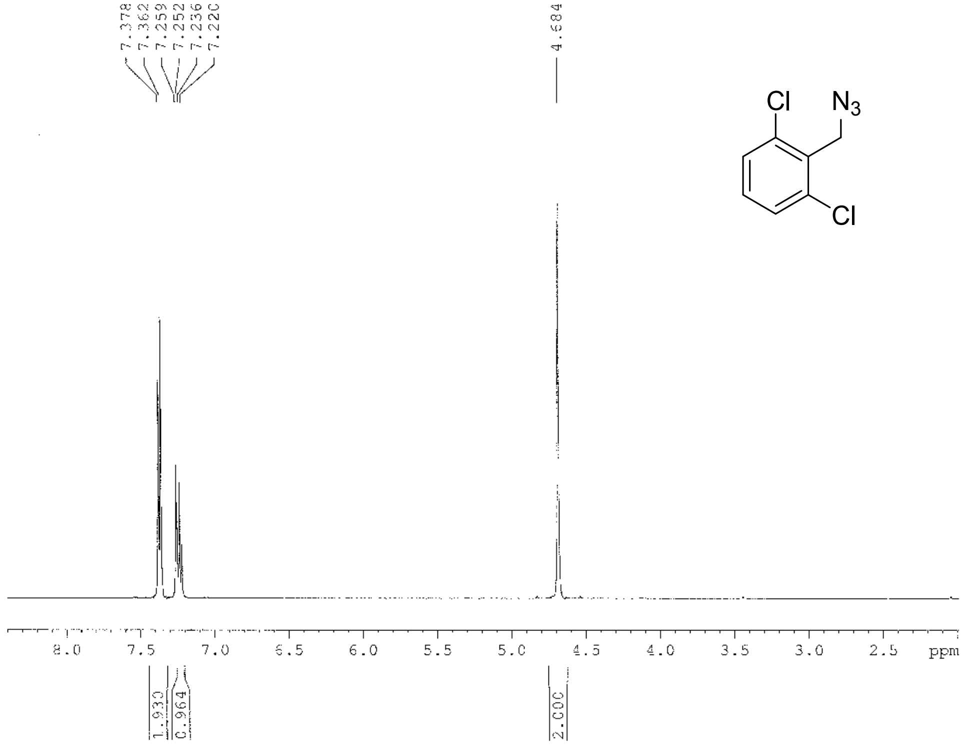
7.378
7.362
7.259
7.252
7.236
7.220
4.684
Cl
N3
Cl
8.0 7.5 7.0 6.5 6.0 5.5 5.0 4.5 4.0 3.5 3.0 2.5 ppm
1.930
0.964
2.000

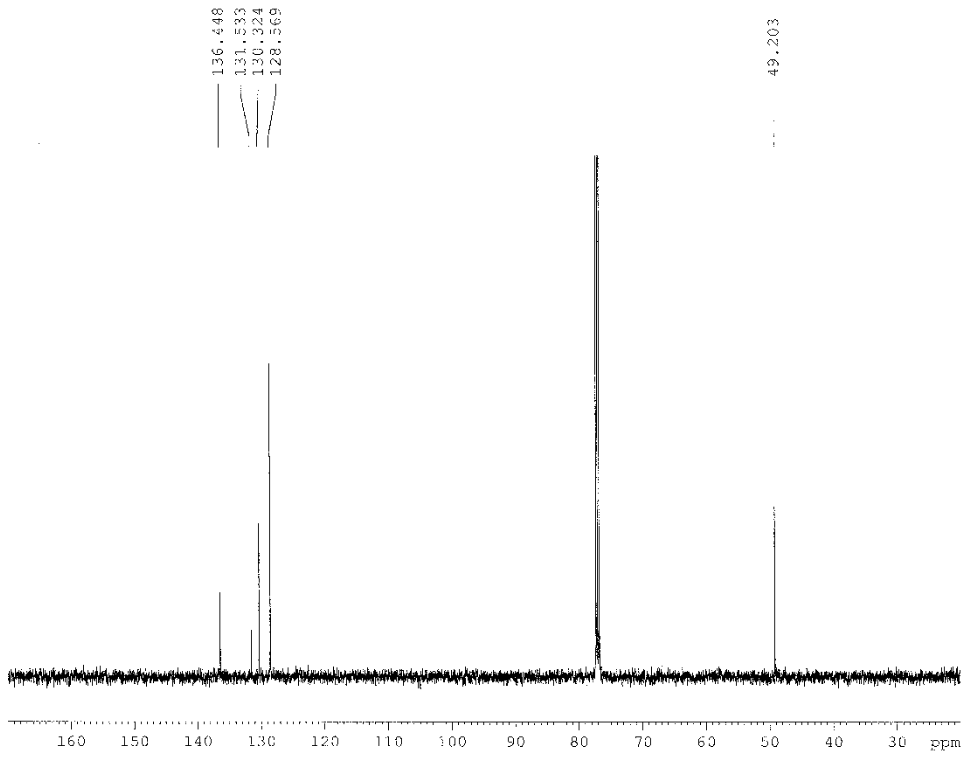
136.448
131.533
130.324
128.569
49.203
160 150 140 130 120 110 100 90 80 70 60 50 40 30 ppm

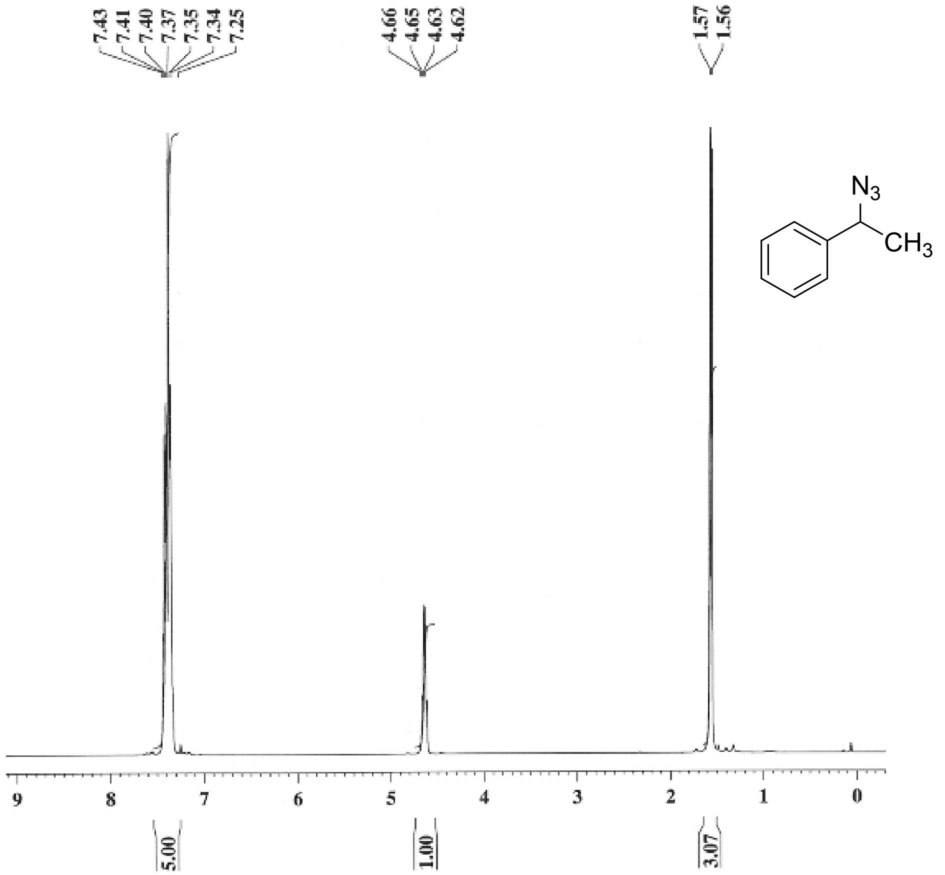
7.43
7.41
7.40
7.37
7.35
7.34
7.25
4.66
4.65
4.63
4.62
1.57
1.56
N3
CH3
5.00
1.00
3.07

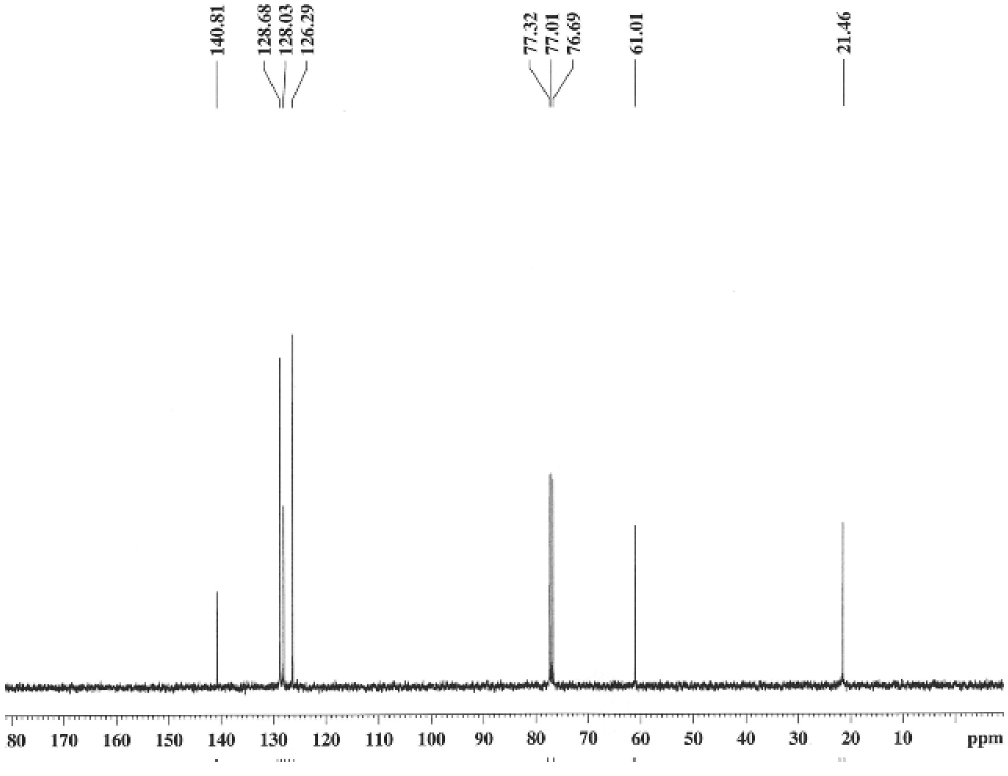
140.81
128.68
128.03
126.29
77.32
77.01
76.69
61.01
21.46
ppm

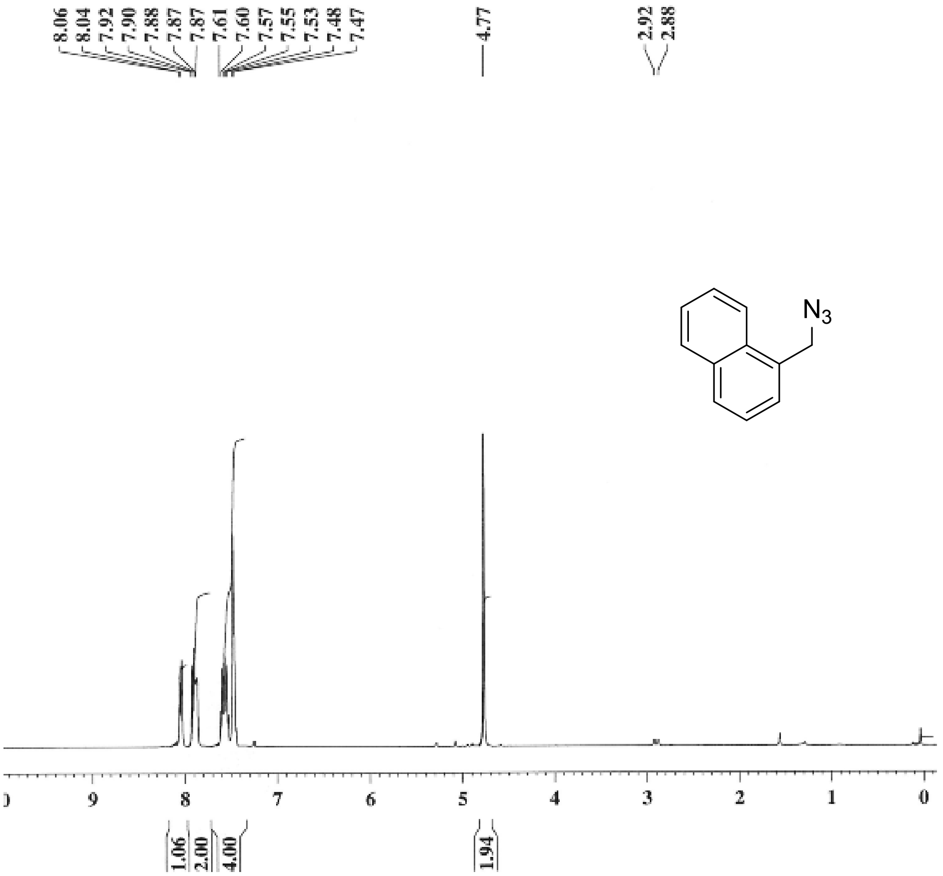
8.06
8.04
7.92
7.90
7.88
7.87
7.87
7.61
7.60
7.57
7.55
7.53
7.48
7.47
4.77
2.92
2.88
N3
9
8
7
6
5
4
3
2
1
0
1.06
2.00
4.00
1.94

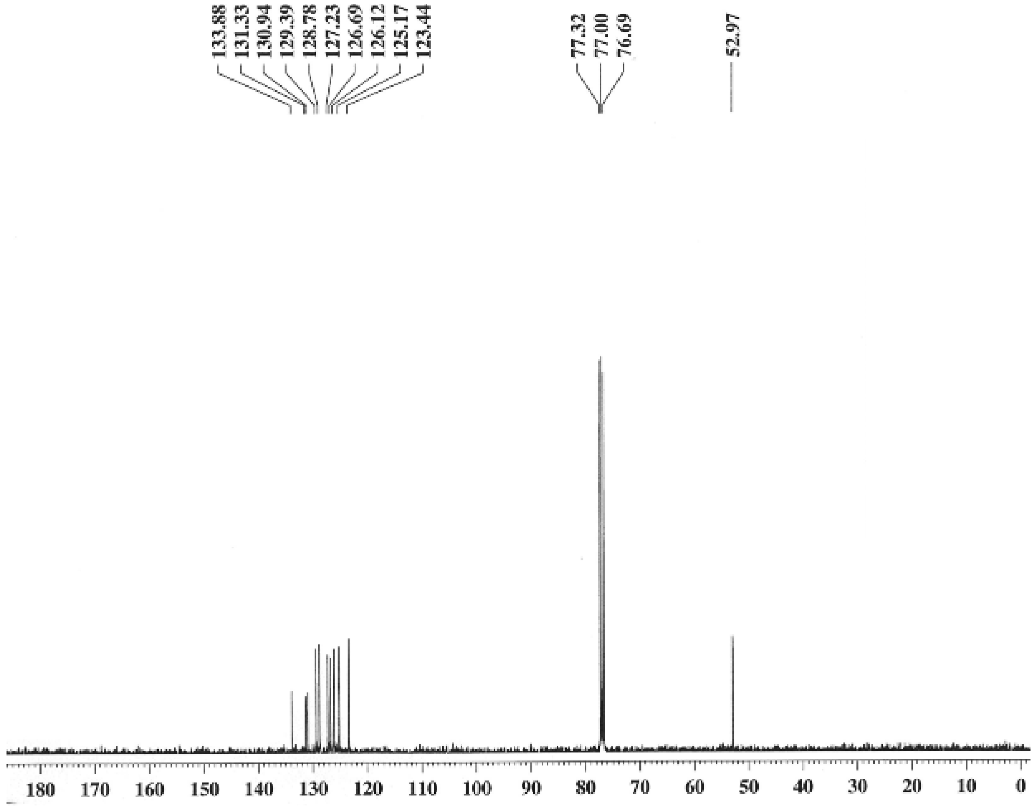
133.88
131.33
130.94
129.39
128.78
127.23
126.69
126.12
125.17
123.44
77.32
77.00
76.69
52.97
180
170
160
150
140
130
120
110
100
90
80
70
60
50
40
30
20
10
0

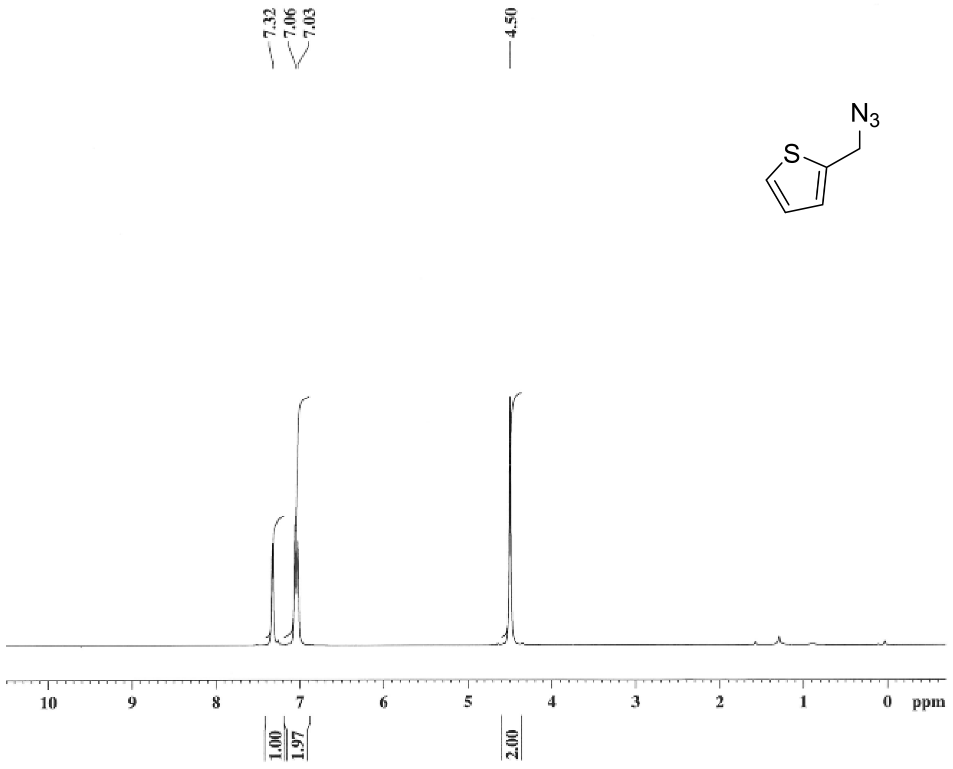
7.32
7.06
7.03
4.50
N3
S
10 9 8 7 6 5 4 3 2 1 0 ppm
1.00
1.97
2.00

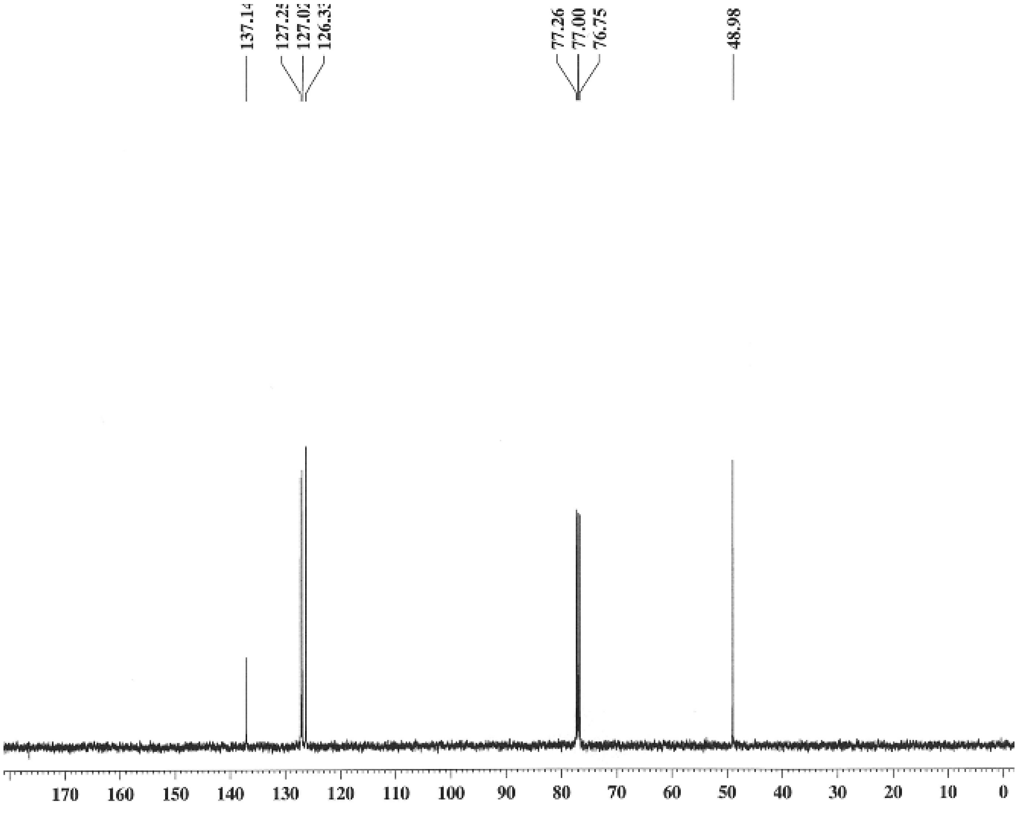
137.14
127.02
77.26
77.00
76.75
48.98
170 160 150 140 130 120 110 100 90 80 70 60 50 40 30 20 10 0

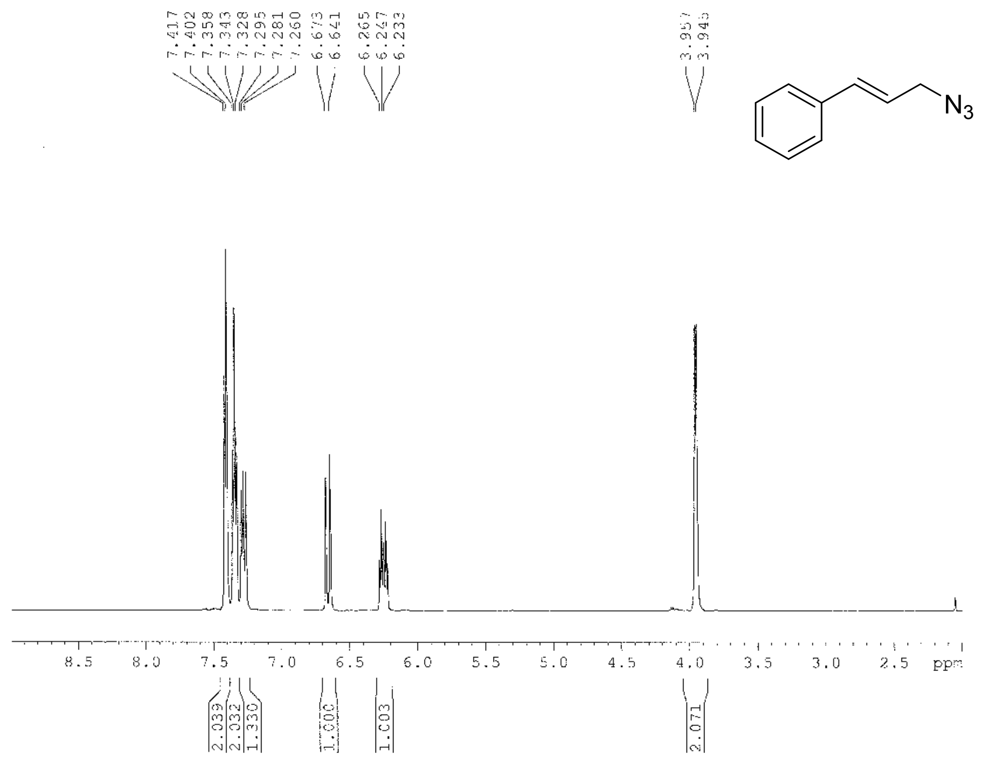
7.417
7.402
7.358
7.343
7.328
7.295
7.281
7.260
6.673
6.641
6.265
6.247
6.233
3.957
3.945
N3
8.5 8.0 7.5 7.0 6.5 6.0 5.5 5.0 4.5 4.0 3.5 3.0 2.5 ppm
2.039
2.032
1.330
1.000
1.003
2.071

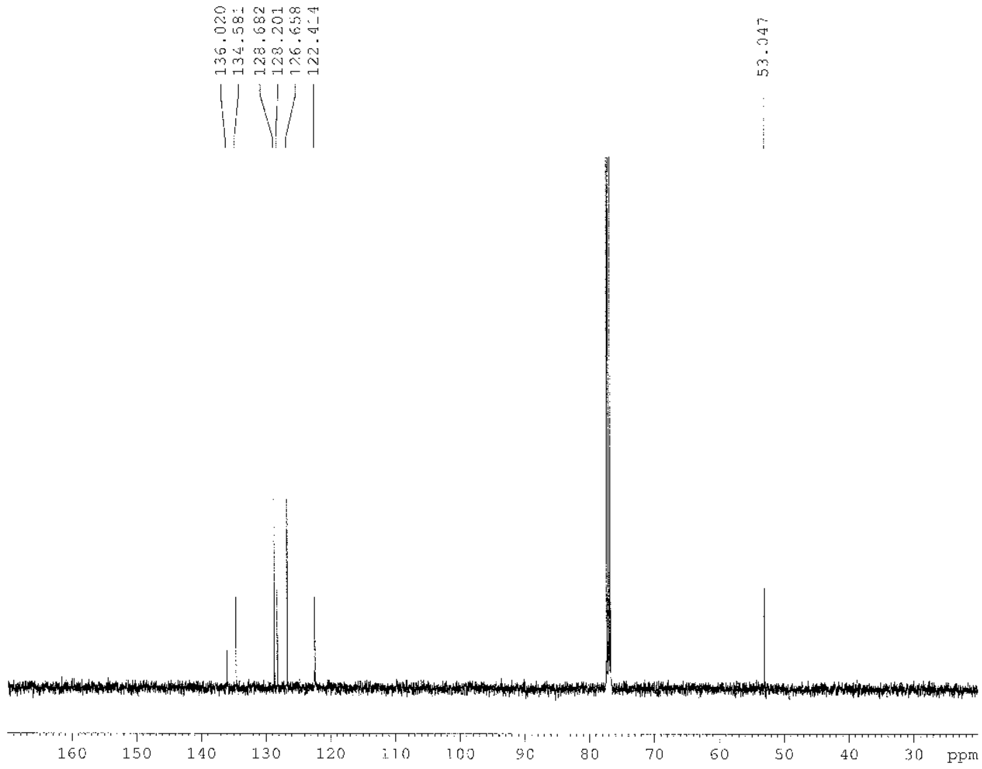
136.020
134.581
128.682
128.201
126.658
122.424
53.047
160 150 140 130 120 110 100 90 80 70 60 50 40 30 ppm

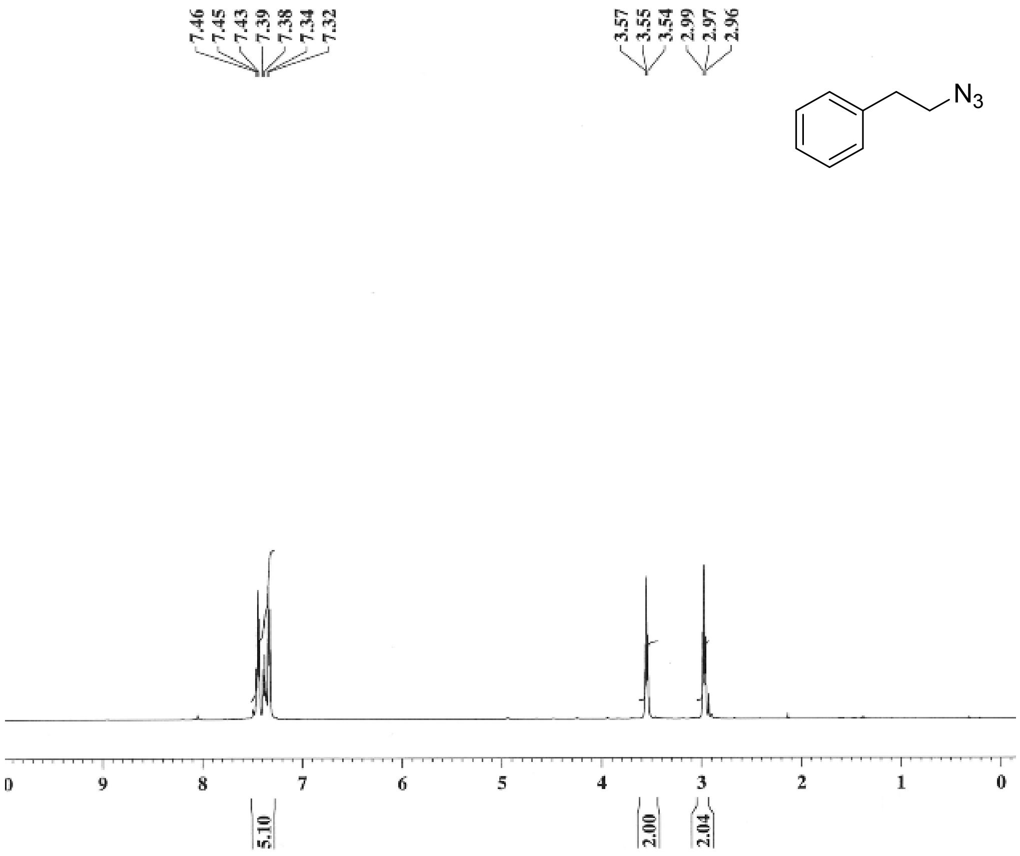
7.46
7.45
7.43
7.39
7.38
7.34
7.32
3.57
3.55
3.54
2.99
2.97
2.96
5.10
2.00
2.04
N3

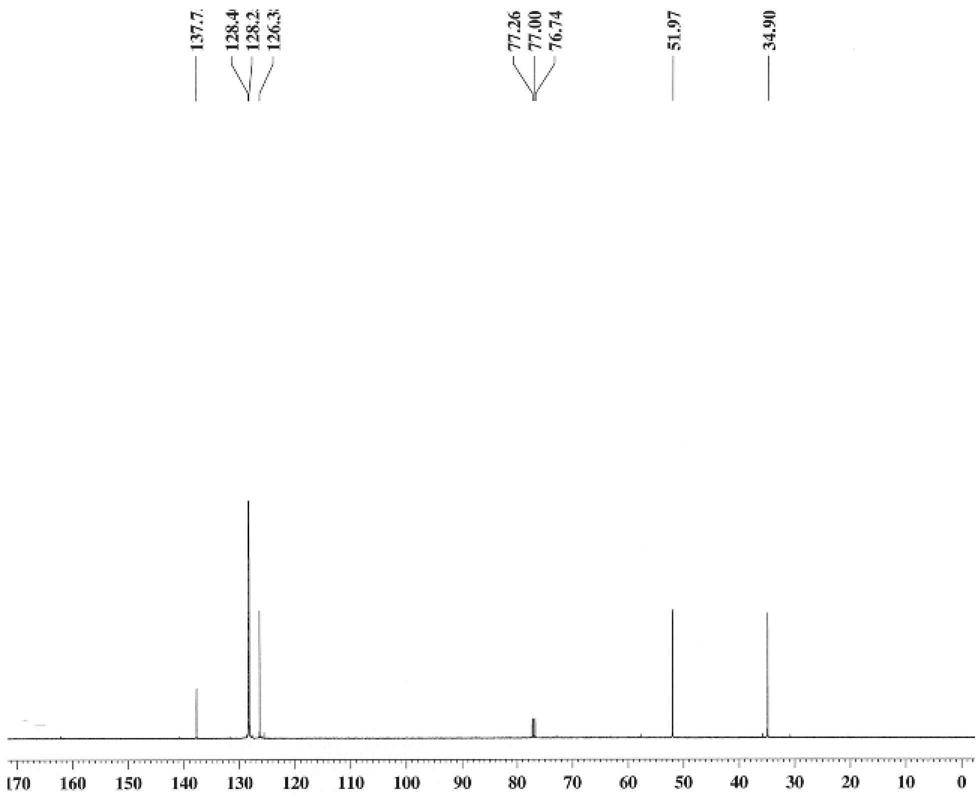
137.7
128.4
128.2
126.3
77.26
77.00
76.74
51.97
34.90

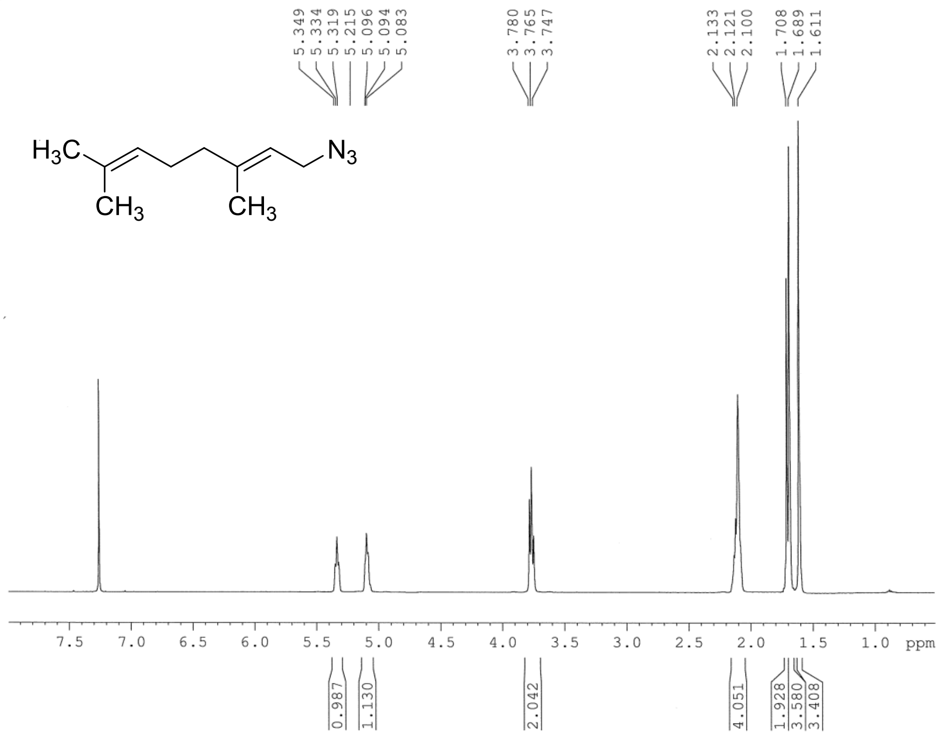
5.349
5.334
5.319
5.215
5.096
5.094
5.083
3.780
3.765
3.747
2.133
2.121
2.100
1.708
1.689
1.611
H3C
CH3
CH3
N3
7.5 7.0 6.5 6.0 5.5 5.0 4.5 4.0 3.5 3.0 2.5 2.0 1.5 1.0 ppm
0.987
1.130
2.042
4.051
1.928
3.580
3.408

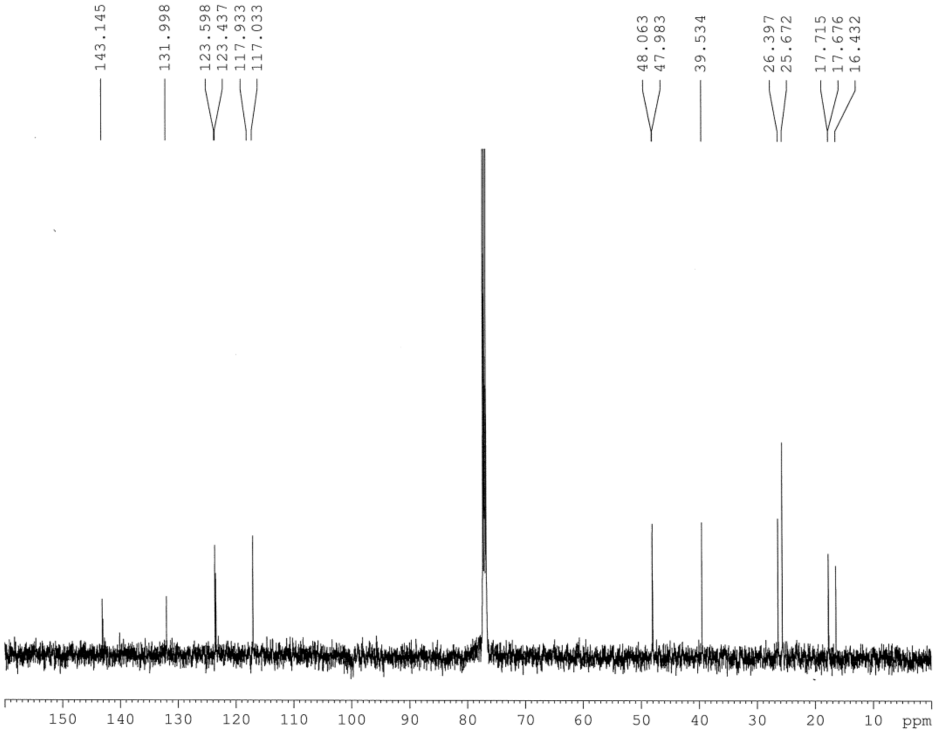
143.145
131.998
123.598
123.437
117.933
117.033
48.063
47.983
39.534
26.397
25.672
17.715
17.676
16.432
150 140 130 120 110 100 90 80 70 60 50 40 30 20 10 ppm

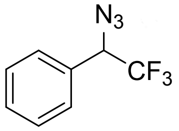
N3
CF3

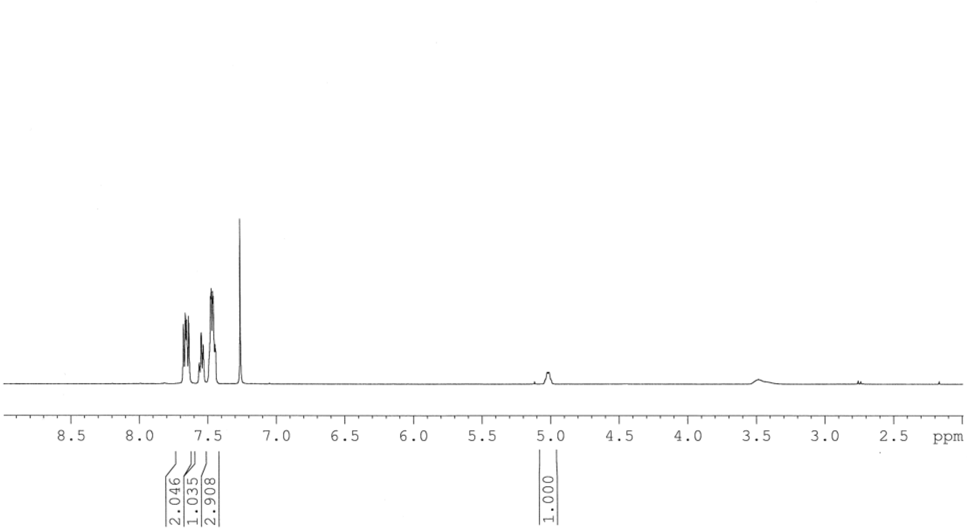
7.674
7.660
7.657
7.650
7.636
7.633
7.558
7.543
7.531
7.529
7.475
7.470
7.460
7.454
7.445
7.439
8.5 8.0 7.5 7.0 6.5 6.0 5.5 5.0 4.5 4.0 3.5 3.0 2.5 ppm
2.046
1.035
2.908
1.000

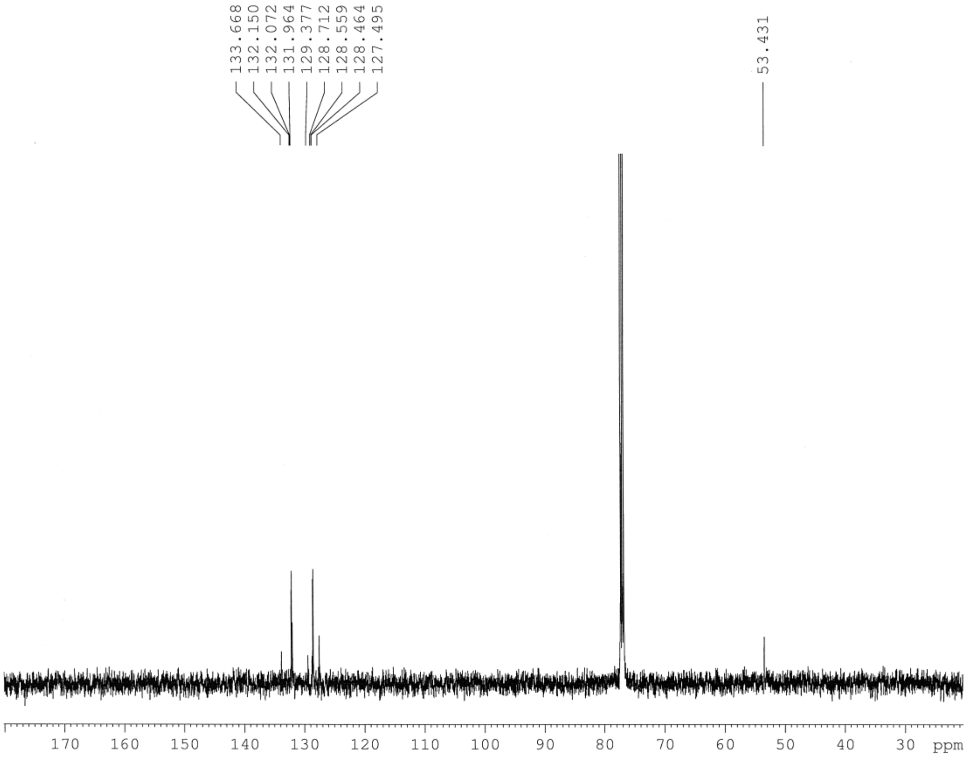
133.668
132.150
132.072
131.964
129.377
128.712
128.559
128.464
127.495
53.431
170 160 150 140 130 120 110 100 90 80 70 60 50 40 30 ppm

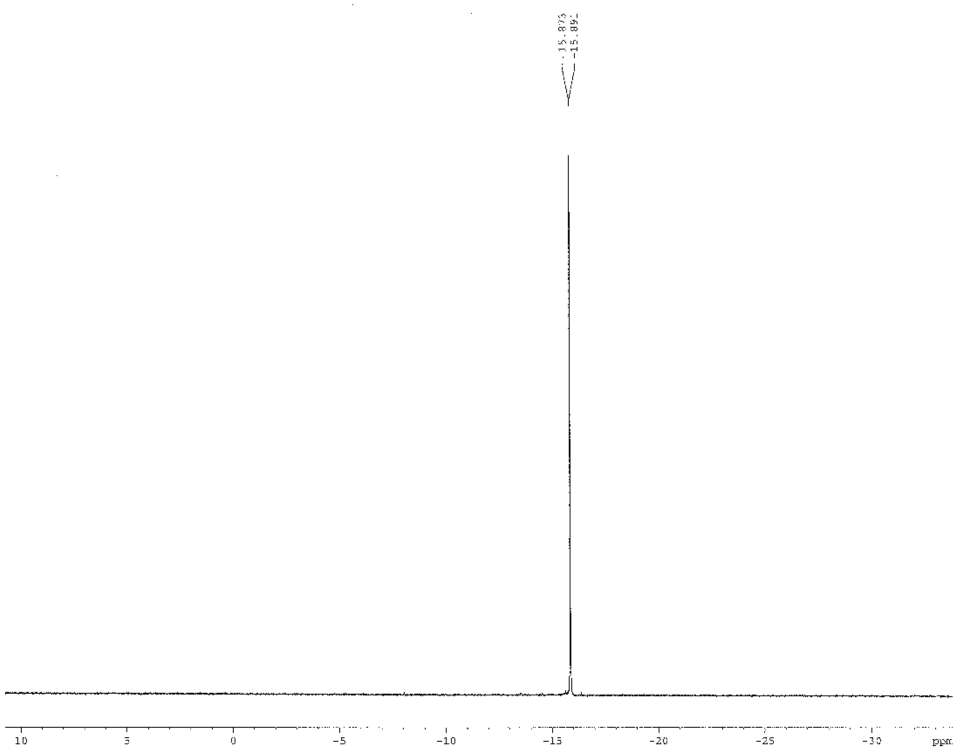
-15.873
-15.892
10
5
0
-5
-10
-15
-20
-25
-30
ppm

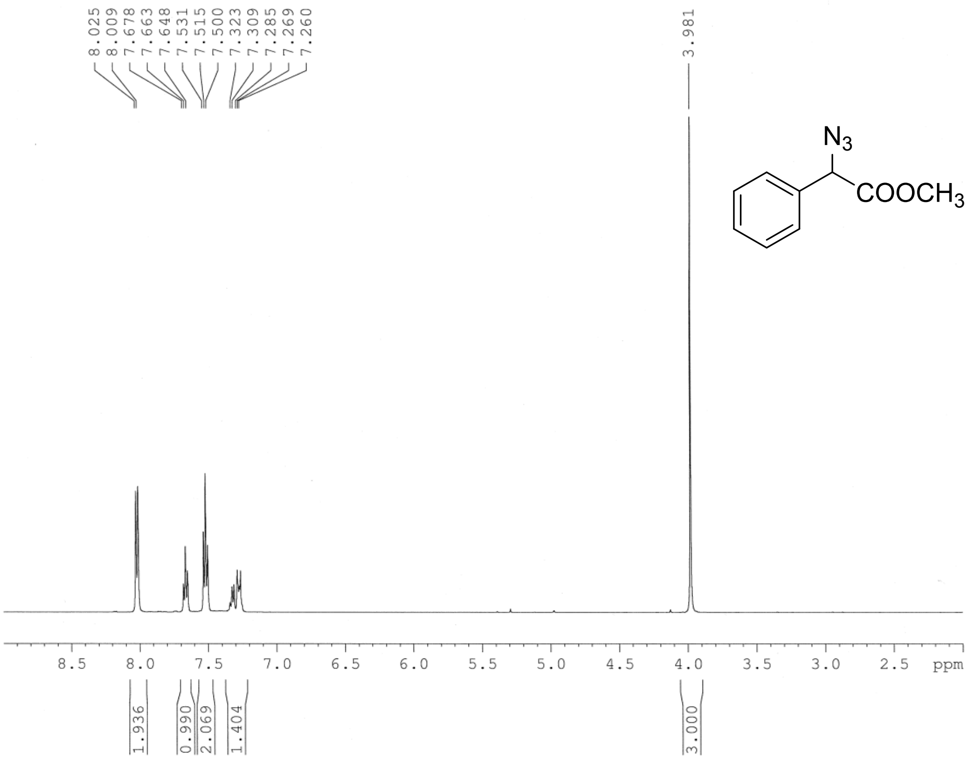
8.025
8.009
7.678
7.663
7.648
7.531
7.515
7.500
7.323
7.309
7.285
7.269
7.260
3.981
N_3
$COOCH_3$
8.5 8.0 7.5 7.0 6.5 6.0 5.5 5.0 4.5 4.0 3.5 3.0 2.5 ppm
1.936
0.990
2.069
1.404
3.000

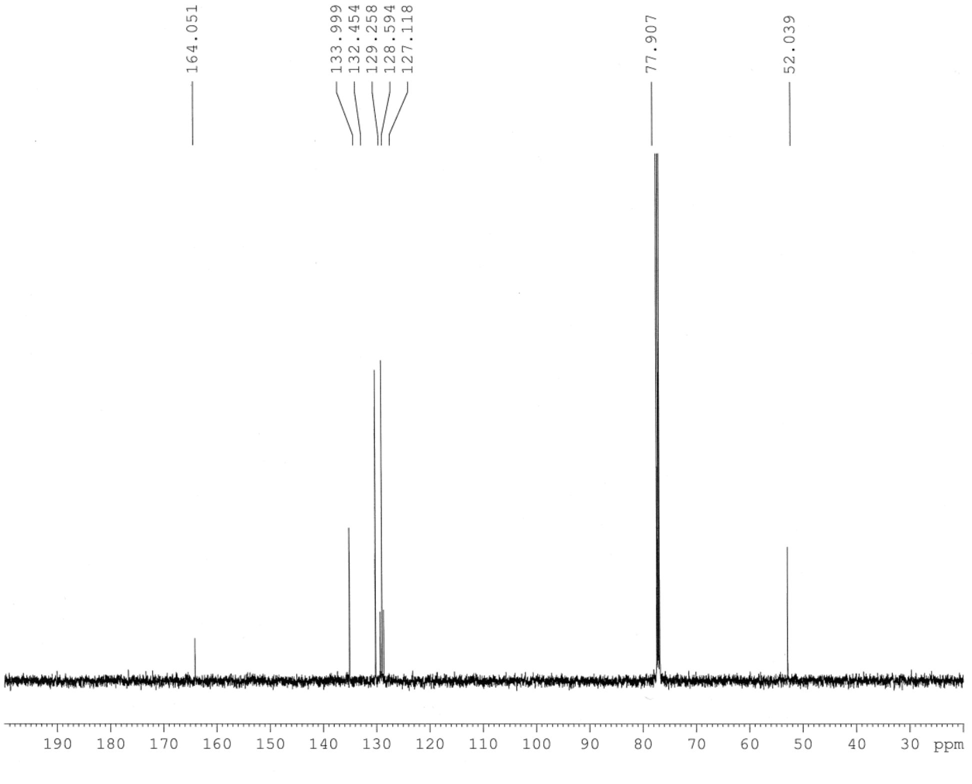
164.051
133.999
132.454
129.258
128.594
127.118
77.907
52.039
190 180 170 160 150 140 130 120 110 100 90 80 70 60 50 40 30 ppm

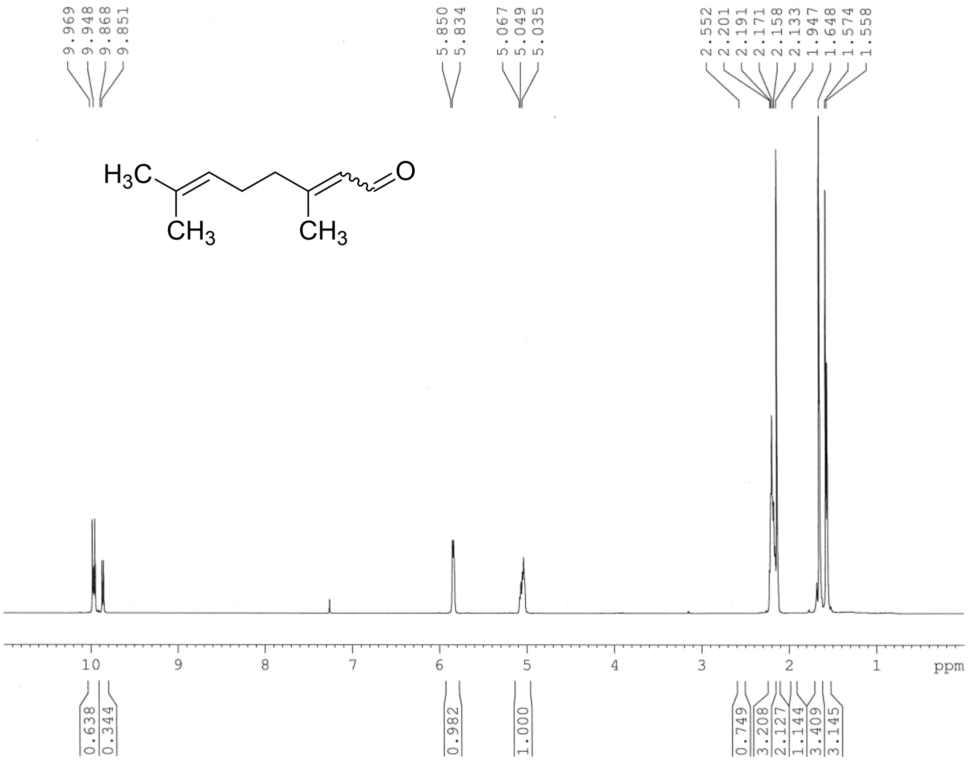

9.969
9.948
9.868
9.851
5.850
5.834
5.067
5.049
5.035
2.552
2.201
2.191
2.171
2.158
2.133
1.947
1.648
1.574
1.558
H_3C
CH_3
CH_3
O
10 9 8 7 6 5 4 3 2 1 ppm
0.638
0.344
0.982
1.000
0.749
3.208
2.127
1.144
3.409
3.145

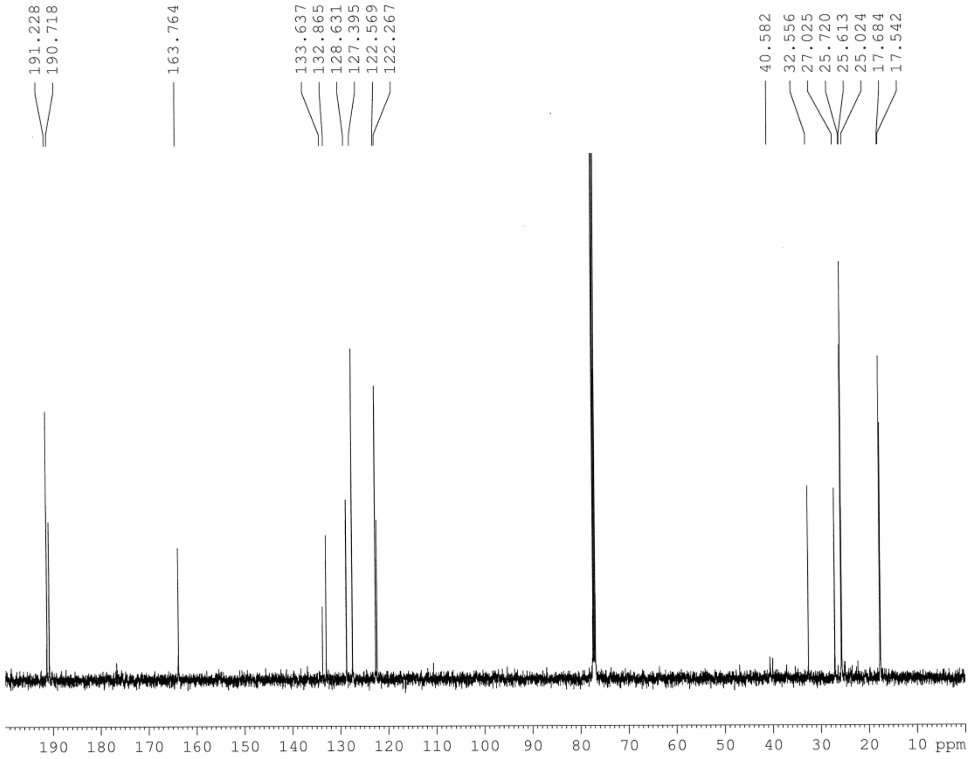

191.228
190.718
163.764
133.637
132.865
128.631
127.395
122.569
122.267
40.582
32.556
27.025
25.720
25.613
25.024
17.684
17.542
190 180 170 160 150 140 130 120 110 100 90 80 70 60 50 40 30 20 10 ppm

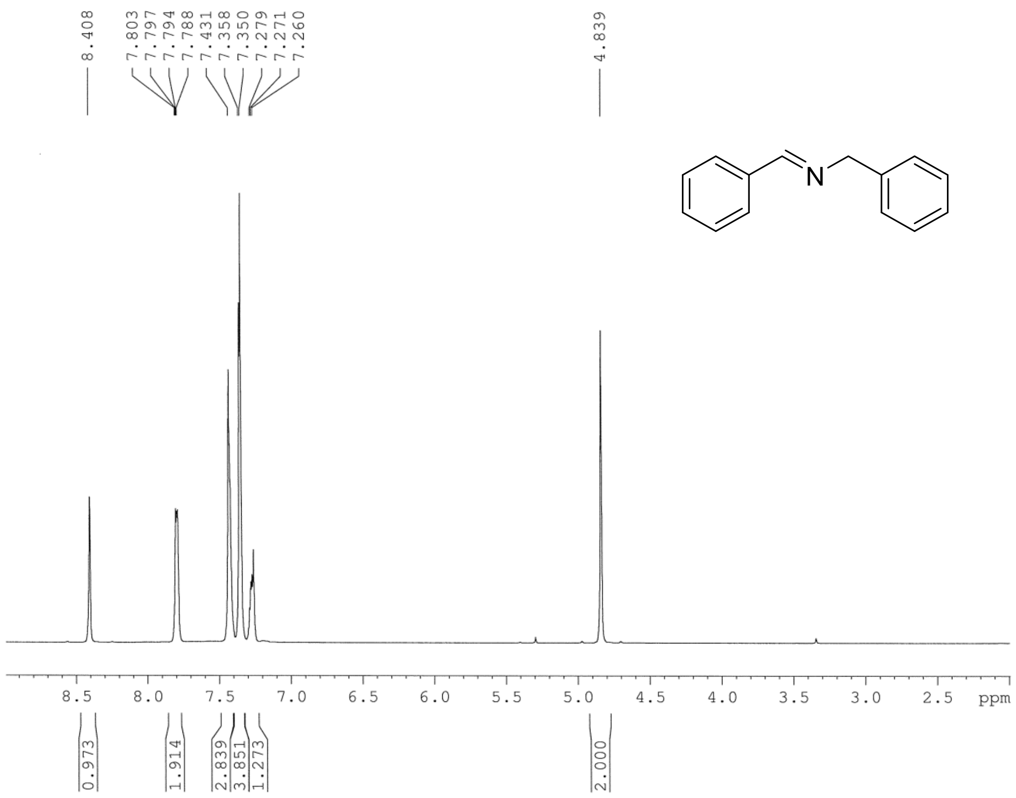
8.408
7.803
7.797
7.794
7.788
7.431
7.358
7.350
7.279
7.271
7.260
4.839
N
8.5 8.0 7.5 7.0 6.5 6.0 5.5 5.0 4.5 4.0 3.5 3.0 2.5 ppm
0.973
1.914
2.839
3.851
1.273
2.000

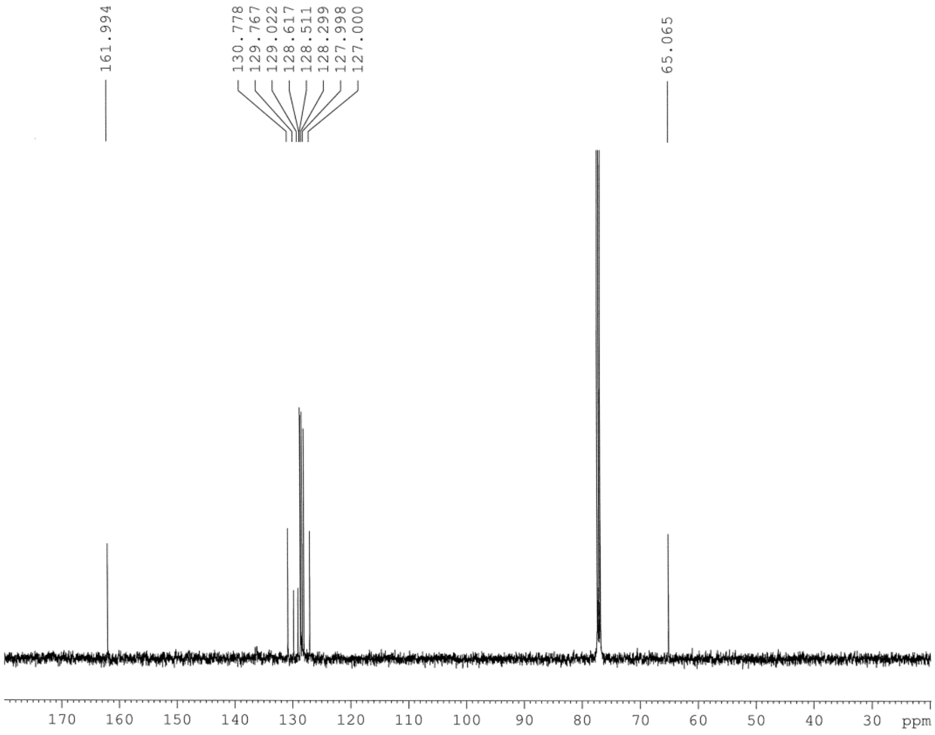
161.994
130.778
129.767
129.022
128.617
128.511
128.299
127.998
127.000
65.065
170 160 150 140 130 120 110 100 90 80 70 60 50 40 30 ppm
